# Palladium‐Doping‐Enabled Interface Synergy for Superb Ampere Level Ammonia Electrosynthesis From Nitrate

**DOI:** 10.1002/anie.2794664

**Published:** 2026-05-18

**Authors:** Qun He, Chuanqiang Wu, Zhangsheng Shi, Dongxue Yu, Wei Jiang, Li Song, Xin Wang

**Affiliations:** ^1^ Department of Chemistry City University of Hong Kong Kowloon China; ^2^ State Information Materials and Intelligent Sensing Laboratory of Anhui Province Key Laboratory of Structure and Functional Regulation of Hybrid Materials of Ministry of Education Institutes of Physical Science and Information Technology Anhui University Hefei China; ^3^ National Synchrotron Radiation Laboratory University of Science and Technology of China Hefei China

**Keywords:** Ab initio molecular dynamics, interface behavior, isolated palladium sites, nitrate reduction, reaction mechanism

## Abstract

The practical electrocatalytic nitrate‐to‐ammonia conversion suffers from insufficient performance at industrial current densities. Here, we report an electrochemically reconstructed Pd‐doped Co catalyst that achieves > 98.0% ammonia Faradaic efficiency at an ultrahigh partial current density of ‒1.43 A cm^−2^ and operates stably for over 170 h. Integrated in situ spectroscopy and theoretical simulations reveal a dual‐enhancement mechanism, in which Pd doping lowers the energy barrier of the rate‐determining *NO hydrogenation step and reconstructs the interfacial microenvironment. This restructuring promotes cation enrichment and establishes a dynamic hydrogen‐down‐oriented water network, facilitating proton transfer and intermediate stabilization under high‐flux conditions. Unlike its role in Cu‐based systems, Pd in the Co matrix distinctly optimizes the kinetics of surface intermediates, enhances the local interfacial electric field, and increases the flexibility of the hydrogen‐bond network. This work demonstrates the critical synergy between atomic‐site engineering and microenvironment control for achieving high‐rate electrocatalysis beyond conventional electronic modulation.

## Graphical abstract

Isolated Pd sites in a metallic Co matrix boost nitrate‐to‐ammonia conversion. Pd doping optimizes intermediate adsorption and reconstructs the interfacial microenvironment, promoting cation enrichment and a hydrogen‐down‐oriented water network. This dual modulation facilitates proton transfer and intermediate stabilization, enabling high ammonia selectivity at ampere‐level current densities with excellent stability.

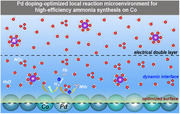

## 1 Introduction

The growing global concerns over widespread nitrate pollution in water bodies and the significant energy demands of the century‐old Haber‐Bosch process have intensified research into the electrochemical nitrate reduction reaction (NO_3_RR) [1, 2, 3, 4, 5]. NO_3_RR presents a dual benefit: it can simultaneously remediate nitrate‐contaminated water and produce valuable ammonia—a key chemical fertilizer and potential carbon‐free energy carrier—under ambient conditions powered by renewable electricity [6, 7, 8, 9, 10]. Despite these promising prospects, the practical implementation of NO_3_RR technology faces intrinsic catalytic challenges. The reaction involves a complex network of eight‐electron/nine‐proton transfer steps, generating various nitrogenous intermediates, which often results in poor selectivity for the desired ammonia product [11, 12, 13]. Although recent advancements have demonstrated catalysts with high Faradaic efficiency (FE) for ammonia (> 90%), maintaining their performance at industrially relevant currents (> ‒0.5 A cm^−2^) over extended periods remains challenging [14, 15, 16, 17, 18, 19, 20]. Under such demanding conditions, characterized by high overpotentials and substantial reactant flux, mass transport limitations and the competing hydrogen evolution reaction (HER) become dominant, creating a critical performance bottleneck.

Cobalt (Co)‐based materials have emerged as promising NO_3_RR catalysts due to their favorable affinity for nitrate adsorption and moderate hydrogen binding strength, which can potentially suppress HER [21, 22]. However, monometallic Co sites often present a compromised adsorption landscape that cannot optimally stabilize the diverse range of reaction intermediates along the pathway to ammonia, leading to insufficient activity and selectivity [23, 24, 25, 26]. Introducing a second metal to create bimetallic systems has proven effective in tailoring the electronic structure and optimizing the adsorption energies of key species [27, 28, 29]. Beyond the intrinsic activity of the active sites, the local reaction microenvironment at the catalyst‐electrolyte interface is increasingly recognized as a decisive, yet underexplored, factor influencing catalytic outcomes [30, 31, 32]. Our recent work has highlighted the importance of optimizing catalytic surfaces and strategically organizing the interface environment to improve ammonia selectivity in NO_3_RR [33]. In essence, the structure, orientation, and hydrogen‐bond network of interfacial water molecules, coupled with the distribution and solvation state of charge‐balancing cations, collectively dictate proton transfer kinetics and intermediate stabilization—factors that become critically limiting under high‐current operation. A fundamental understanding and deliberate engineering of this dynamic interfacial environment are therefore essential for overcoming the ubiquitous activity‐selectivity trade‐off in high‐rate electrocatalysis.

In this study, we introduce a palladium‐doped cobalt disulfide pre‐catalyst (Pd‐CoS_2_) that, upon electrochemical activation, transforms into a catalyst with isolated Pd atoms embedded in a metallic Co matrix (activated Pd‐CoS_2_). This reconstructed catalyst delivers exceptional NO_3_RR performance, achieving an ammonia FE exceeding 98.0% at a partial current density surpassing ‒1.4 A cm^−2^ and maintaining high stability for over 170 h. We systematically demonstrate that this benchmark performance arises from a synergistic dual modulation mechanism: (i) atomic‐level Pd doping regulates the electronic structure of surface sites, optimizing key intermediate adsorption and reducing the kinetic barriers of the key elementary steps. (ii) More critically, the incorporated Pd atoms induce a comprehensive reconstruction of the interfacial microenvironment, promoting the formation of a dynamic water network and facilitating cation enrichment, which collectively ensure efficient proton supply and intermediate stabilization under high‐flux conditions, as evidenced by in situ Raman spectroscopy and ab initio molecular dynamics (AIMD) simulations. This study provides a paradigm for designing high‐performance electrocatalysts through the cooperative regulation of atomic sites and the interfacial microenvironment, offering generalizable insights for other multi‐step electrocatalytic transformations.

## 2 Results and Discussion

### 2.1 Structural Evolution During Electrochemical Processes

**FIGURE 1:**
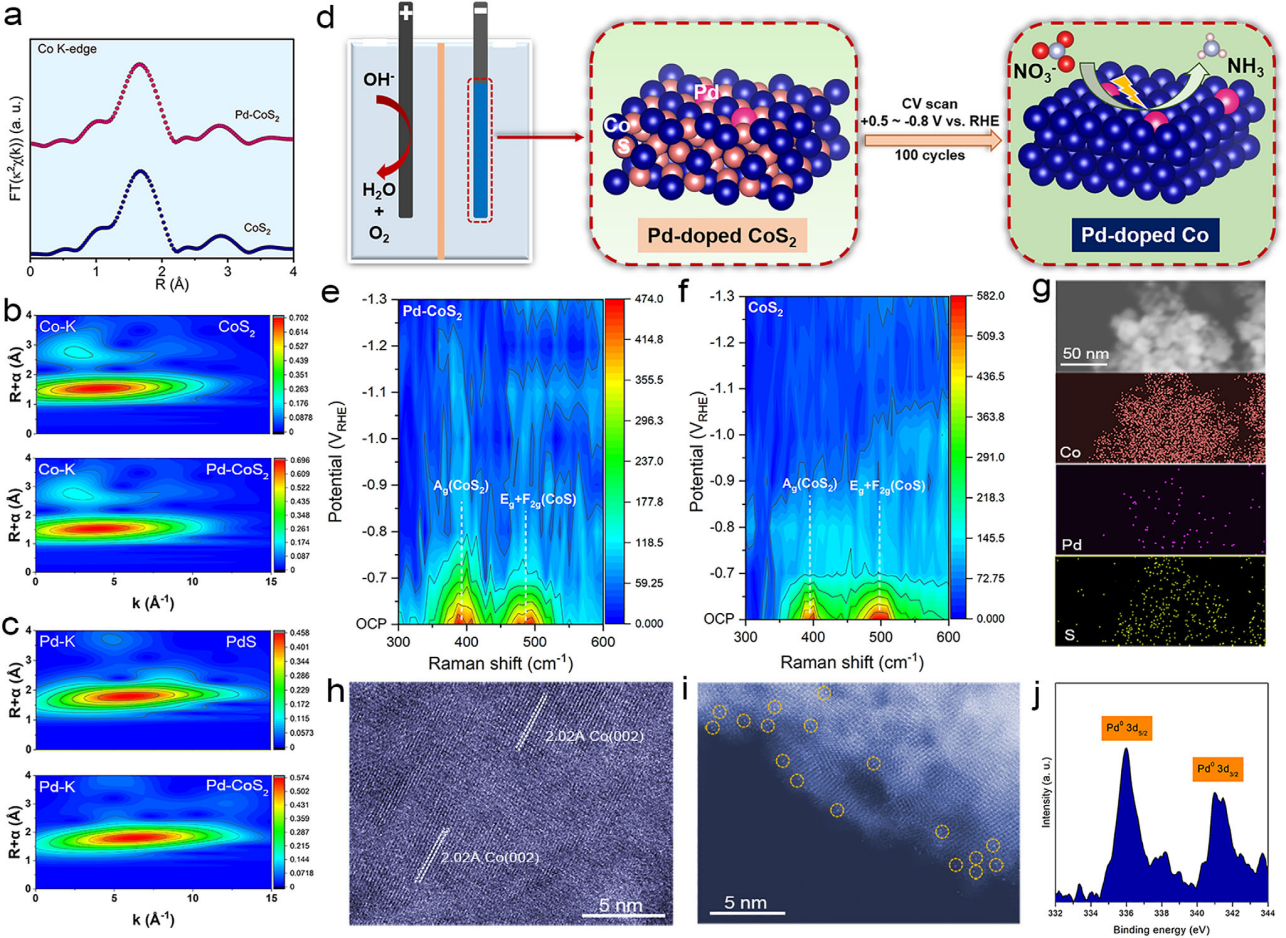
(a) Co K‐edge FT‐EXAFS spectra of Pd‐CoS_2_ and CoS_2_. Wavelet‐transform EXAFS analysis of (b) CoS_2_ and Pd‐CoS_2_ at the Co K‐edge and (c) PdS and Pd‐CoS_2_ at the Pd K‐edge. (d) Schematic illustration of the transformation from Pd‐CoS_2_ to isolated Pd‐doped metallic Co under electrochemical conditioning. (e, f) In situ Raman spectra of (e) Pd‐CoS_2_ and (f) pure CoS_2_ under applied potentials. (g) Elemental mapping of Co, S, and Pd in activated Pd‐CoS_2_. (h) HRTEM image of post‐reaction Pd‐CoS_2_. (i) Atomic‐resolution HAADF‐STEM image confirming the presence of isolated Pd sites in post‐reaction Pd‐CoS_2_. (j) High‐resolution Pd 3d XPS spectrum of the post‐reaction Pd‐CoS_2_.

The catalyst precursors (Co_2_(OH)_3_Cl and Co_2_(OH)_3_Cl‐Pd) were prepared via a one‐step wet‐chemistry method (Figures S1 and S2, details in Experimental Section). Pd‐CoS_2_ pre‐catalyst was synthesized using a high‐temperature vulcanization method (details provided in the Experimental Section). Comprehensive characterization through various analytical techniques confirmed the successful formation of pyrite‐type CoS_2_ and the incorporation of isolated Pd atoms (Figures S3–S5). Elemental analysis via inductively coupled plasma‐atomic emission spectrometry (ICP‐AES) determined the Pd/Co molar ratio to be 0.75% in Pd‐CoS_2_ (Table S1). X‐ray photoelectron spectroscopy (XPS) revealed significant modifications in the electronic structure due to Pd doping. The Pd‐CoS_2_ exhibited a notably higher proportion of Co^3+^ species compared to CoS_2_, indicating successful Pd integration into the CoS_2_ lattice (Figures S6 and S7) [34]. This electronic alteration was further supported by Co L_2,3_‐edge X‐ray absorption near‐edge structure (XANES) spectroscopy, which displayed distinct spectral changes (Figure S8). Co and Pd K‐edge XANES spectra confirmed the retention of the primary CoS_2_ phase while verifying successful Pd incorporation. Fourier‐transform extended X‐ray absorption fine structure (FT‐EXAFS) and wavelet‐transform EXAFS (WT‐EXAFS) analyses showed that the incorporated Pd atoms preferentially formed Pd‐S coordination bonds, confirming the atomic dispersion of Pd dopants within the CoS_2_ matrix, consistent with high‐angle annular dark‐field scanning transmission electron microscopy (HAADF‐STEM) observations (Figure 1a–c, Figures S9 and S10).

The activated Pd‐CoS_2_ catalyst was generated through electrochemical activation via cyclic voltammetry (CV) over a wide potential window under reaction conditions (Figure 1d). The sulfide phase gradually transforms into sulfite and/or sulfate during the CV cycling (Figure S11). The isolated Pd‐modified Co serves as the active structure for nitrate‐to‐ammonia electrocatalysis. First, the XRD pattern (Figure S12a) of the catalyst after the long‐term stability test shows no diffraction peaks corresponding to CoS_2_, indicating a significant phase transformation during electrochemical NO_3_RR. Instead, diffraction peaks of metallic Co appear, suggesting deep reduction of the catalyst to a metallic phase. Some signals attributable to β‐Co(OH)_2_ are also observed, likely due to rapid phase conversion of Co upon removal of the applied potential. The selected area electron diffraction (SAED) pattern shows the same phase composition as that of XRD (Figure S12b). Second, in situ Raman spectroscopy was used to monitor the structural evolution of Pd‐CoS_2_ and CoS_2_. Upon applying a working potential of −0.7 V versus the reversible hydrogen electrode (V vs. RHE), the characteristic CoS_2_ phonon mode at ∼397 cm^−1^ rapidly diminished and eventually disappeared at more negative potentials for Pd‐CoS_2_ (Figure 1e). The absence of new vibrational peaks supports that β‐Co(OH)_2_ forms after potential removal. These results suggest that maintaining the true active metallic phase requires protection under negative working potentials. A process similarly observed for undoped CoS_2_ (Figure 1f). Elemental mapping analysis revealed a distinct Co‐rich matrix with a sparse distribution of both S and Pd (Figure 1g), further indicating substantial reconstruction of the original sulfide structure during CV activation. Notably, high‐resolution transmission electron microscopy (HRTEM) and atomic‐resolution HAADF‐STEM provided direct evidence for the formation of metallic Co and the preservation of isolated Pd sites in the activated Pd‐CoS_2_ catalyst (Figure 1h,i). This isolated Pd‐modified metallic Co configuration was identified as the active phase for NO_3_RR electrocatalysis. XPS of the post‐reaction samples showed almost undetectable S species, confirming the thorough breakdown of the original Co‐S lattice framework (Figures S13 and S14) [35]. In addition, Pd predominantly exists in the metallic state, while Co and O mainly exhibit characteristic signals of β‐Co(OH)_2_, along with a minor Co^0^ signal, consistent with the XRD result (Figure 1j and Figure S13) [36]. Collectively, these analytical results provide compelling evidence that metallic Co, derived from sulfide pre‐catalysts through an electrochemical reconstruction process, serves as the true active phase for NO_3_RR, regardless of the presence of isolated Pd sites. The preserved isolated Pd sites likely play a crucial role in enhancing the catalytic performance of the Co matrix, as will be discussed in subsequent sections.

### 2.2 Electrochemical Nitrate Reduction Performance

**FIGURE 2:**
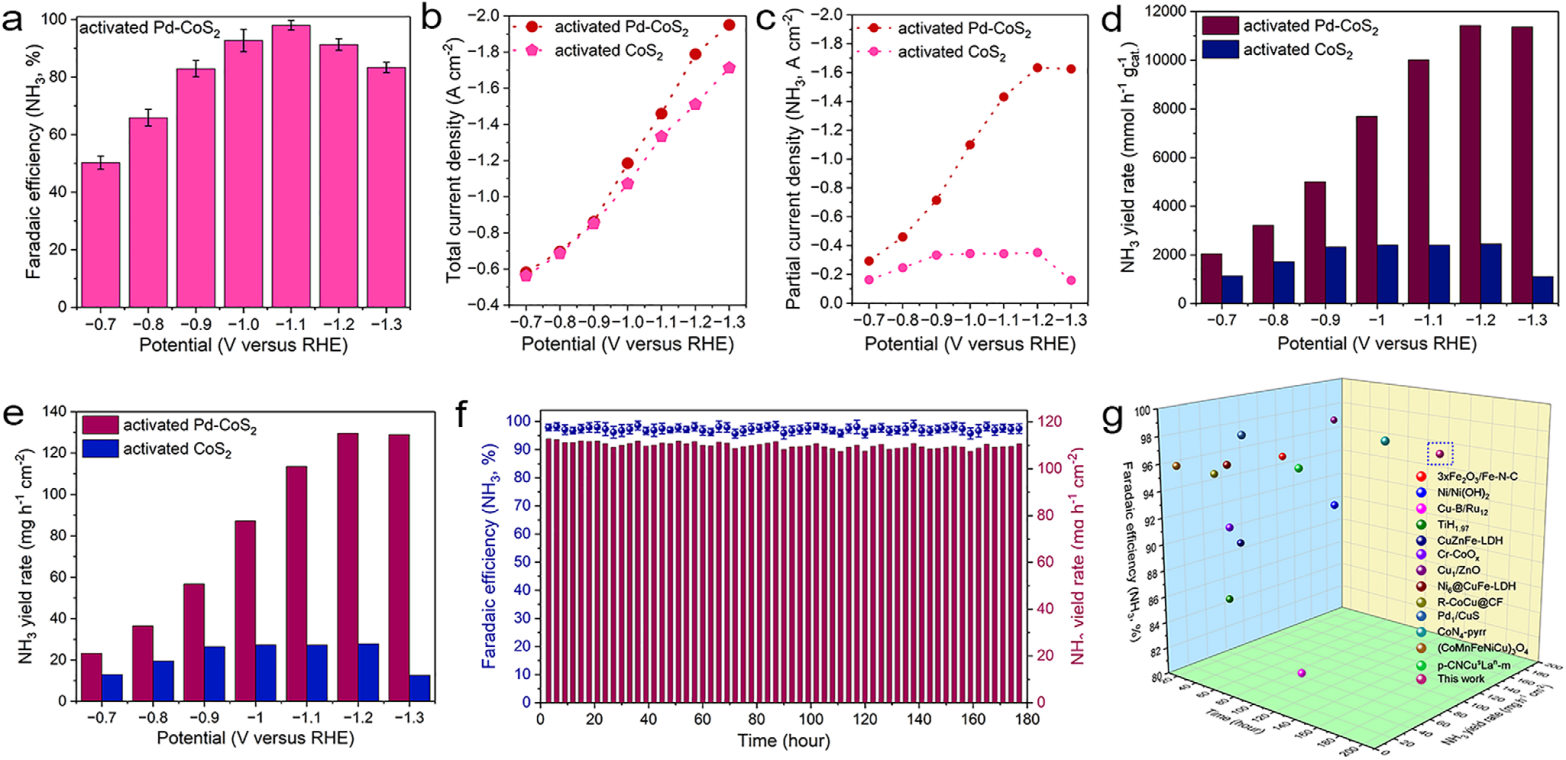
(a) Ammonia Faradaic efficiency of activated Pd‐CoS_2_ across applied potentials. Comparison of total current densities (b) and ammonia partial current densities (c) for activated Pd‐CoS_2_ and CoS_2_. (d) Mass‐normalized and (e) geometric area‐normalized ammonia yield rates. (f) Long‐term stability test at ‒1.1 V versus RHE. (g) Performance comparison of our main catalyst with recently reported NO_3_RR catalysts in the literature.

The electrochemical performances of the activated Pd‐CoS_2_ catalyst for NO_3_RR were assessed in a 1.0 M KOH aqueous electrolyte containing 0.5 M KNO_3_ as the nitrate source (Figures S15 and S16). The catalyst demonstrated ammonia FE exceeding 50.2% across the entire potential range, peaking at over 98.0% at ‒1.1 V versus RHE (Figure 2a) [33]. In contrast, activated CoS_2_ showed significantly lower selectivity (< 40.0%) under identical conditions (Figure S17). The incorporation of Pd consistently enhanced ammonia selectivity, highlighting its crucial role in the nitrate‐to‐ammonia conversion process (Figure S18). Even in an electrolyte with lower nitrate concentration, our catalyst maintained substantial NO_3_RR performance (Figure S19). Regarding the applied potentials, the more negative potential used in our work is attributed to the intrinsic properties of monometallic Co. As reported by Carvalho et al., Co shows strong potential‐dependent selectivity: HER dominates at less negative potentials, while NO_3_RR becomes predominant only under sufficiently negative conditions [37]. This mechanistic behavior accounts for the potential discrepancy compared to Cu‐based or other bimetallic systems, which achieve high selectivity at lower potentials. The electrocatalytic origin of the NH_4_^+^ product was conclusively confirmed through ^15^N isotope‐labeling experiments. The characteristic doublet signal of ^15^NH_4_^+^ in ^1^H nuclear magnetic resonance (NMR) spectra was exclusively observed when using ^15^NO_3_^−^ as the reactant, verifying the catalytic conversion pathway (Figure S20).

Beyond selectivity, activated Pd‐CoS_2_ demonstrated outstanding catalytic activity, as evidenced by significantly elevated current densities. The NO_3_RR partial current density increased continuously with applied potential up to ‒1.2 V versus RHE, reaching an impressive ‒1.63 A cm^−2^ (Figure 2b,c). In comparison, activated CoS_2_ exhibited limited performance, with a maximum current density of only ‒0.35 A cm^−2^ and clear saturation beyond ‒0.9 V versus RHE. This pronounced activity enhancement underscores the efficacy of Pd incorporation in promoting high‐current NO_3_RR operation.

Quantitative analysis further revealed an exceptional ammonia yield rate of 11429 mmol h^−1^ g^−1^ at ‒1.2 V versus RHE for activated Pd‐CoS_2_—4.66 times higher than that of activated CoS_2_ (2455 mmol h^−1^ g^−1^) (Figure 2d). A similar enhancement was observed in geometric area‐normalized yield rates, with a 4.17‐fold increase at ‒1.1 V versus RHE (Figure 2e). Critically, the catalyst exhibited remarkable stability under high‐current conditions, maintaining an average FE of > 97.2% at industrial‐level current densities (>‒1.4 A cm^−2^) over 170 h, outperforming most reported NO_3_RR catalysts (Figure 2f,g) [14, 19, 33, 38, 39, 40, 41, 42, 43, 44, 45, 46, 47]. Post HAADF‐STEM, XPS, and elemental mapping analyses demonstrated the structural maintenance of our catalyst (Figures S21–S23). The absence of significant sulfate interference further corroborates the structural and catalytic robustness (Figure S24). Actually, in situ Raman spectra show that no significant SO_x_^2−^ species are present during the reaction, further suggesting that the residual SO_x_^2−^ species do not contribute to the catalytic performance (Figure S25).

Collectively, these results underscore the superior activity, high selectivity, and exceptional stability—especially under high current densities—of activated Pd‐CoS_2_, establishing it as a highly efficient and industrially relevant electrocatalyst for nitrate‐to‐ammonia conversion.

### 2.3 Mechanistic Insights Into Pathway and Interface

**FIGURE 3:**
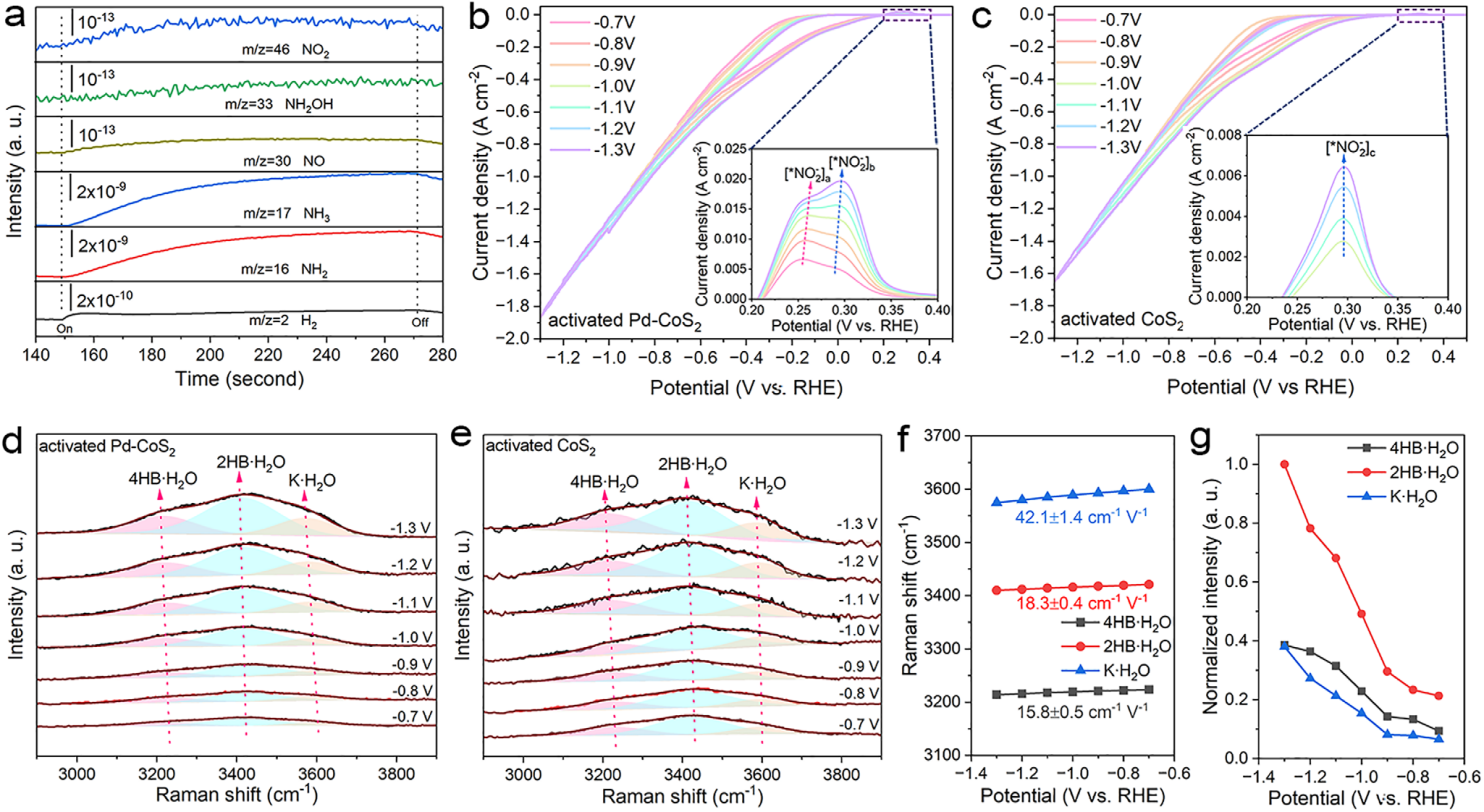
(a) Online DEMS analysis of activated Pd‐CoS_2_ revealing key intermediates during NO_3_RR. (b, c) CV curves of activated (b) Pd‐CoS_2_ and (c) CoS_2_ in nitrate‐containing electrolyte under different cathodic limits. (d, e) In situ Raman spectra and deconvolution of the O‐H stretching region for activated (d) Pd‐CoS_2_ and (e) CoS_2_. (f) Potential‐dependent frequency shifts of interfacial water species on activated Pd‐CoS_2_. (g) Normalized intensity of H_2_O configurations for activated Pd‐CoS_2_.

Through comprehensive electrochemical and spectroscopic analyses, we elucidate the mechanistic origins of the enhanced NO_3_RR performance of the activated Pd‐CoS_2_ catalyst, establishing a direct link to its high performance. Online differential electrochemical mass spectrometry (DEMS) identified key reaction intermediates (*NH_2_, *NH_3_, *NO, *NH_2_OH, and *NO_2_), supporting the proposed reaction pathway: *NO_3_^−^ → *NO_2_^−^ → *NO → *NHO → *NH_2_OH → *NH_3_ (Figure 3a and Figure S26). This mechanistic framework provides fundamental insights into the catalytic cycle essential for achieving high ammonia selectivity under industrially relevant current densities.

The CV characterization revealed that activated Pd‐CoS_2_ exhibited well‐defined NO_2_^−^ oxidation peaks within a narrow potential window (+0.5 to −0.7 V vs. RHE), whereas activated CoS_2_ required a wider window (+0.5 to −1.0 V vs. RHE) to manifest detectable signals (Figure 3b,c). This demonstrates that Pd incorporation substantially enhances NO_3_^−^‐to‐NO_2_^−^ conversion efficiency while suppressing competing hydrogen evolution. The control CV experiments conducted in the absence of KNO_3_ show no oxidation peaks detected under these conditions, strongly supporting the peak assignments (Figure S27). Notably, the emergence of an additional oxidation peak corresponding to weakly adsorbed NO_2_^−^ ([*NO_2_^−^]_a_) on activated Pd‐CoS_2_ suggests modified adsorption configurations with optimized binding energetics. Moreover, the stronger oxidation peaks of activated Pd‐CoS_2_ compared to activated CoS_2_ suggest the formation of NO_2_^−^ adsorption‐dominant sites rather than other adsorbents, such as hydroxyl (*OH), improving the coverage of *NO_2_^−^. However, more negative oxidation potentials indicate that Pd incorporation alters the surface electronic structure, weakening the adsorption for NO_2_^−^ while regulating the energy barriers of other elementary steps. Remarkably, both *NO_2_^−^ oxidation peaks on activated Pd‐CoS_2_ showed positive potential shifts upon cathodic window expansion, indicating possible intermediate coverage‐induced repulsion effects, demonstrating the high‐efficiency generation of key intermediates on the activated Pd‐CoS_2_ surface with heterogeneous sites. The accumulation of NO_3_RR‐involving intermediates is crucial for achieving high partial current density of ammonia production. In stark contrast, activated CoS_2_ demonstrated poor *NO_2_^−^ conversion efficiency and exhibited negligible potential‐dependent peak shifts, reflecting its electrochemical inflexibility. This unchanged peak position also implies that the catalytic sites are mutually independent with no strong interactions between sites in activated CoS_2_. From another perspective, it can be understood that the surface‐adsorbed *OH from water dissociation is relatively more abundant than *NO_2_^−^; thereby, these oxidation peaks are mainly determined by *NO_2_^−^ coverage on activated CoS_2_. Dominant *OH occupation implies that interfacial water molecules are strongly adsorbed, and the diffusion of intermediates is limited due to a poorly dynamic interface layer. However, the surface state of activated Pd‐CoS_2_ has been significantly altered. These findings collectively highlight the crucial role of Pd incorporation in site and electrochemical interface regulations for efficient nitrate reduction.

We also observed that maximum ammonia selectivity occurs when the ratio of weakly ([*NO_2_^−^]_a_) to strongly ([*NO_2_^−^]_b_) adsorbed NO_2_^−^ approaches unity ([*NO_2_^−^]_a_/[*NO_2_^−^]_b_ close to 1, Figure S28). This optimal balance operates through a synergistic mechanism: strongly adsorbed sites mainly participate in sequential hydrogenation of NO_3_RR intermediates, while weakly adsorbed sites mainly facilitate the supply of protons, collectively enabling efficient nitrate reduction. This cooperative adsorption modulation fundamentally explains the maintained high selectivity even at elevated current densities.

The multi‐proton/electron transfer nature of NO_3_RR necessitates understanding interfacial properties. H/D kinetic isotope effect (KIE) studies showed that replacing H_2_O with D_2_O led to a decrease in the NO_3_RR rates for both catalysts, underscoring the essential role of *H in the reaction process (Figure S29). However, the KIE values of both catalysts were very small, and the observed value for activated Pd‐CoS_2_ was even lower. This indicates that both catalysts have high water activation and dissociation capabilities, and the hydrogen transfer kinetics on the activated Pd‐CoS_2_ are accelerated. This enhanced proton supply capability is crucial for sustaining high reaction rates under large current conditions.

In situ Raman spectroscopy further revealed distinct interfacial water structures (Figure 3d,e). A broad O‐H stretching band (3000–3800 cm^−1^) exhibited progressive intensity enhancement with decreasing potentials, revealing potential‐mediated interfacial reconstruction. Spectral deconvolution resolved three distinct water configurations: K^+^ hydrated water (K·H_2_O, ∼3601 cm^−1^), 2‐coordinated hydrogen‐bonded water (2HB·H_2_O, ∼3421 cm^−1^), and 4‐coordinated hydrogen‐bonded water (4HB·H_2_O, ∼3224 cm^−1^).

Vibrational Stark effect analysis revealed contrasting interfacial behaviors: activated CoS_2_ exhibited a very small slope value of approximately 11.3 cm^−1^ V^−1^, suggesting that K^+^ ions with relatively complete and dynamic hydrated layers mainly locate at the outer Helmholtz layer and show weak interactions with the electrode surface (Figure S30). Additionally, the slope of 2HB·H_2_O is also small (approximately 13.3 cm^−1^ V^−1^), suggesting these interfacial water molecules are solidly stabilized on the electrode surface, forming an interface water layer composed of structurally rigid and dynamically poor water molecules, which are restricted by orientation and dynamics. The slope of 4HB·H_2_O (approximately 27.4 cm^−1^ V^−1^) is also lower than that reported in the literature, implying the interface electric field is not strong [48]. The above analysis indicates that on the activated CoS_2_, a weakly interacting hydration layer and a rigid and dynamic‐poor interface water layer have formed. This environment is not conducive to the efficient transfer of protons and the stabilization of reaction intermediates that require cations to stabilize charges. The reaction may be more inclined toward simple 2‐electron processes such as HER caused by competing with the rigid interface water, thereby reducing the selectivity of ammonia production.

In striking contrast, activated Pd‐CoS_2_ exhibited dominant potential‐dependent shifts of approximately 42.1 cm^−1^ V^−1^ for K·H_2_O, demonstrating the significant change of the cation hydration layer (Figure 3f). One reason is that Pd incorporation alters the electronic structure of the catalyst, strengthening the interactions between K^+^ and the electrode surface, thereby concentrating hydrated K^+^ near the electrode surface. The slope of 2HB·H_2_O also rises (approximately 18.3 cm^−1^ V^−1^), suggesting the orientation and motion of these adsorbed interfacial water molecules are more flexible, which might also originate from the regulated surface electronic structure and the enhanced interfacial electric field. As for 4HB·H_2_O, its slope is significantly reduced to 15.8 cm^−1^ V^−1^, indicating the response of this water layer close to the bulk water side to the electric field is weaker. This is probably due to the effective shielding and consumption of the electric field by the innermost K+ and the interfacial 2HB·H_2_O.

The above analyses demonstrated the significant interface difference between activated Pd‐CoS_2_ and CoS_2_. Pd incorporation has positively regulated the interfacial microenvironment of the catalyst for high‐performance ammonia production. The key points can be summarized as follows: (i) the strong local electric field and innermost K^+^ accelerate water dissociation (Volmer step: * + H_2_O + e^−^→ *H + OH^−^), ensuring abundant *H supply; (ii) the dynamic interfacial water layer (2HB·H_2_O) enables rapid proton transfer and exchange, which can also be revealed by the even steeper increase of 2HB·H_2_O for activated Pd‐CoS_2_ (Figures S31 and S32); (iii) the innermost and positively charged hydrated K^+^ ions stabilize negatively charged nitrogen‐containing intermediates (such as NO_2_^−^, NO) through non‐covalent interactions (ion‐dipole interactions), facilitating multi‐electron reduction steps. Additionally, the innermost K^+^ can also stabilize the generated proton through electrostatic forces, preventing them from combining prematurely to form H_2_ and escaping, thereby allowing more protons to be used in the hydrogenation step of NO_3_RR intermediates [49].

In summary, Pd incorporation not only creates additional active sites but also steers both surface adsorption properties and the interfacial microenvironment to simultaneously optimize intermediate stabilization and suppress the rapid recombination of protons. Our experimental results provide a clear picture for explaining the superior performance achieved.

Density functional theory (DFT) calculations reveal that the equilibrium distance between K^+^ and the surface is 3.20 Å on Pd‐doped Co, which is slightly shorter than that on Co (3.25 Å) (Figure S33). This suggests that the introduction of Pd enhances the electrostatic stabilization of K^+^ at the electrode interface, potentially influencing the local electric field and cation distribution. Notably, Pd is well known for its strong H adsorption affinity, which may contribute to the modulation of interfacial water configuration. However, considering the low Pd content, the extent of direct Pd‐H interactions is expected to be limited. Nevertheless, the significant alterations in interfacial water configuration suggest that the primary influence may arise from the modification of the local electrostatic environment, specifically, the enhanced stabilization of K^+^ at the electrode interface, rather than from direct H‐mediated effects alone.

We acknowledge that Pd‐H interactions may still play a secondary role, particularly given Pd's strong H adsorption capability. The observed enhancement in hydrogen bonding near the Pd‐doped Co surface could reflect the combined effects of both mechanisms.

Given the notable difference in in situ Raman spectra between Co‐based catalysts and our recently reported Cu‐based catalysts, we sought to gain further insight into the influence of Pd incorporation on the interfacial behavior of different catalysts. For Cu‐based catalysts, the Stark shifts observed for three distinct types of water molecules are substantially greater than those for Co‐based catalysts, indicating a significantly stronger overall interfacial electric field on Cu‐based surfaces (Figure S34) [33]. This interpretation is further corroborated by the broader adsorption peak of NO_3_^−^ on Cu‐based catalysts compared to Co‐based ones (Figure S35). On activated bare‐Cu catalyst, the intense local electric field induces the largest Stark shift (∼75.7 cm^−1^ V^−1^) for K·H_2_O, reflecting its strong confinement near the electrode surface. As a result, strongly adsorbed intermediates such as *NO_3_ dominate the surface and undergo limited conversion to downstream species. Moreover, the interfacial hydrogen‐bond network is weakened, slowing proton transfer to NO_3_RR intermediates. These adverse effects collectively constrain the performance of bare‐Cu catalyst at a relatively low level.

Upon incorporation of Pd, the local electric field is weakened, as evidenced by the reduced Stark shift of K·H_2_O to 53.0 cm^−1^ V^−1^. This attenuation of the electric field enhances the connectivity of the interfacial hydrogen‐bond network, thereby facilitating proton transfer and modulating the adsorption of key intermediates. Post‐reaction XPS results show an increased ratio of NO_2_^−^ to NO_3_^−^ peaks, suggesting more facile formation and weaker adsorption of NO_2_^−^ (Figure S36). Theoretical analyses indicate that water dissociation and hydrogen evolution processes are modulated in a manner beneficial to NO_3_RR, whereas the energy barriers of key NO_3_RR steps remain largely unchanged [33].

In contrast, Co‐based catalysts exhibit markedly different interfacial behaviors. The bare‐Co catalyst features a weak electric field, exhibiting relatively strong interactions only with 4HB·H_2_O, as indicated by a Stark shift of 27.4 cm^−1^ V^−1^, while shifts for 2HB·H_2_O (13.3 cm^−1^ V^−1^) and K·H_2_O (11.3 cm^−1^ V^−1^) are minimal. This weak electric field is unfavorable for *NO_3_ adsorption. Additionally, interfacial water molecules form a rigid hydrogen‐bond network that hinders efficient proton transfer.

With Pd incorporation, the interfacial structure of Co undergoes a significant reorganization. The response of K·H_2_O to the electric field becomes more pronounced (42.1 cm^−1^ V^−1^), indicating the emergence of a strong local electric field, which also enhances the response of 2HB·H_2_O. The diminished response of bulk‐like 4HB·H_2_O may result from shielding by inner interfacial water and hydrated K^+^. These inner K^+^ ions not only stabilize reaction intermediates but also reduce the rigidity of the interfacial hydrogen‐bond network, promoting a more dynamic microenvironment conducive to proton transfer. In situ Raman results confirm the stabilized adsorption of NO_3_^−^ on Pd‐incorporated Co catalyst, and theoretical analyses reveal that Pd incorporation enhances both water dissociation and *NO hydrogenation. The enhanced rate‐determining *NO hydrogenation process is believed to be one key factor that supports the achievement of high‐rate ammonia conversion.

The beneficial roles of Pd incorporation in CuS and CoS_2_ catalysts are summarized schematically (Figures S37 and S38). Our combined experimental and theoretical results unequivocally demonstrate that Pd doping enhances the NO_3_RR performance of both systems. However, the underlying regulation mechanisms are highly dependent on the intrinsic interfacial properties of the host catalyst. Consequently, the modified surfaces impart distinct functions to key reaction steps, highlighting the critical role of optimizing surface elementary processes—particularly for achieving high‐rate performance. Collectively, these findings underscore the importance of systematic, mechanism‐driven studies to rationalize the performance of electrocatalytic systems.

### 2.4 Theoretical Calculations and Simulations

**FIGURE 4:**
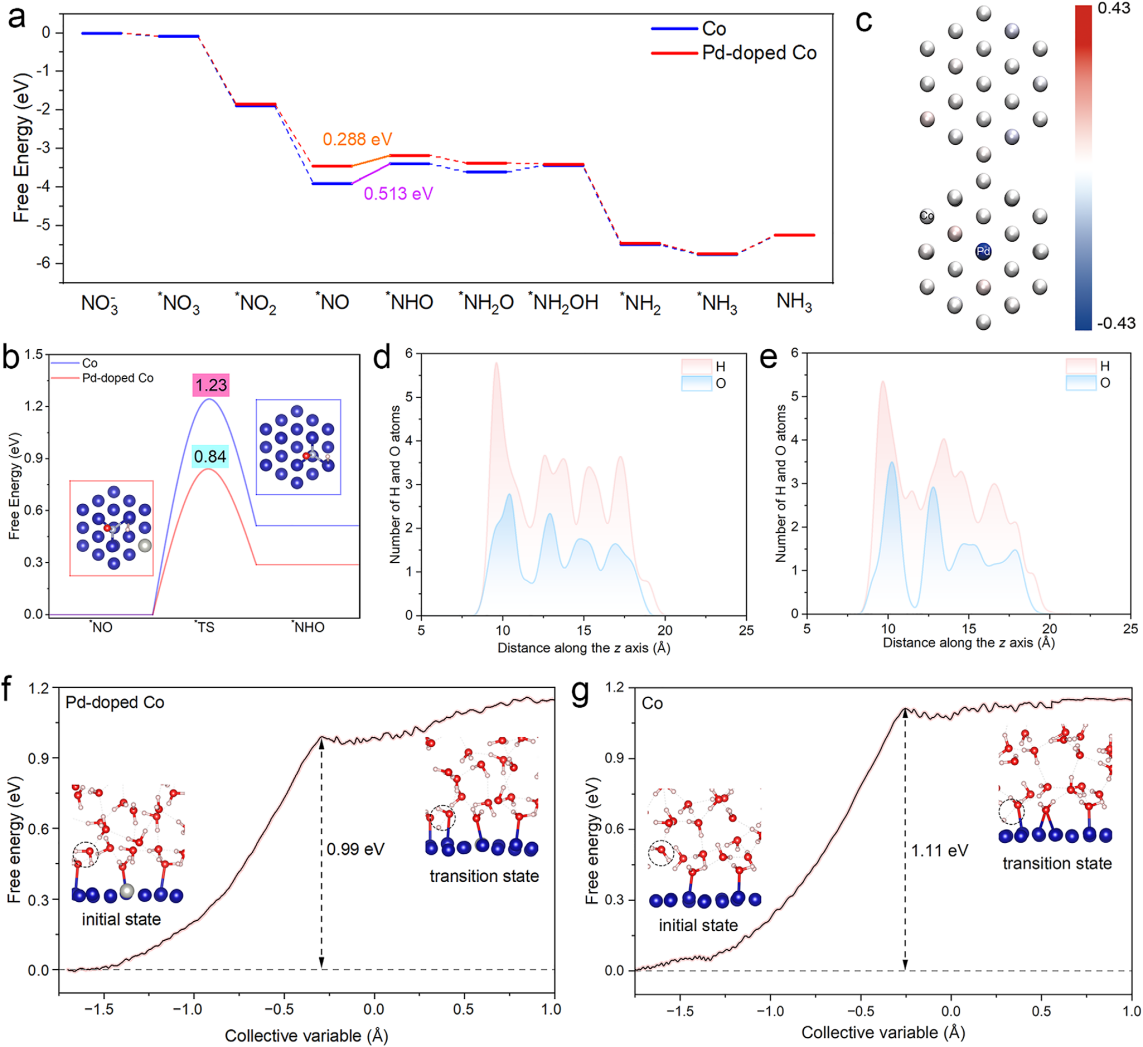
(a) Proposed reaction pathway and corresponding free energy diagrams for NO_3_RR on Co and Pd‐doped Co surfaces. (b) Energy profiles and optimized structures of the transition states for the *NO hydrogenation step on Co and Pd‐doped Co. (c) Bader charge analysis of Co and Pd atoms. (d, e) The number of O and H atoms in water molecules along the z direction normal to the catalyst surface for Pd‐doped Co and Co, respectively, derived from AIMD simulations. (f, g) Energy profiles and molecular trajectories for water dissociation on Pd‐doped Co and Co surfaces, respectively.

Our findings indicate that under operational conditions, the pristine sulfide precatalysts undergo in situ transformation into metallic Co and isolated Pd‐doped Co catalysts. Accordingly, the atomic models (Figures S39 and S40) were constructed to explore the role of Pd in enhancing NO_3_RR performance via DFT calculations. The nitrate conversion into ammonia involving the intermediates of *NO_3_, *NO_2_, *NO, *NHO, *NH_2_O, *NH_2_OH, *NH_2_, and NH_3_, was thoroughly investigated (Figure 4a, Figures S41 and S42). The hydrogenation of *NO was identified as the rate‐determining step (RDS) for both Co and Pd‐doped Co catalysts. As shown in Figure 4a, the thermodynamic barrier for the RDS over the Co surface (0.51 eV) is reduced to 0.29 eV with the introduction of Pd atoms. Meanwhile, the kinetic barriers of NO hydrogenation were also calculated (Figure 4b) and found to be significantly lower on the Pd‐doped Co surface (0.84 eV) compared to the pristine surface (1.23 eV). This reduction in both thermodynamic and kinetic barriers underscores the intrinsic excellence of the isolated Pd‐doped Co catalyst toward NO_3_RR performance.

Besides intrinsically enhanced NO_3_RR performance, the impact of Pd atoms on the oriented transfer of protons in H_2_O was also investigated using ab initio molecular dynamics (AIMD) simulations (Figure S43). The difference in charge density analysis revealed delocalized electron distributions forming metal bonds involving both Co and Pd atoms (Figure S44). Bader charge analysis identified Pd atoms in a negatively charged state (Pd^δ−^), having acquired 0.43 e^−^ from their surroundings (Figure 4c). This charge redistribution critically influences water orientation. The Coulomb interaction between Pd^δ−^ and H^δ+^ in water molecules promotes H‐down orientation on Pd‐doped Co surfaces, as evidenced by the increased separation between the first peaks of H and O atoms in spatial distribution functions (Figure 4d,e) [32]. The optimized water orientation guided by Pd atoms was more prone to drop the protons onto the surface for the hydrogenation of intermediates. This could be confirmed by the relatively improved interfacial hydrogen bond number and the promoted H_2_O dissociation process, in which the kinetic barrier on Pd‐doped Co was reduced to 0.99 eV compared to the pure Co catalyst (1.11 eV) (Figure 4f,g, and Figure S45). The guided interfacial water structure and enhanced proton transfer kinetics are particularly crucial for maintaining high Faradaic efficiency and stability under high current operation, where rapid proton supply becomes limiting.

Collectively, Pd incorporation simultaneously enhances NO_3_RR performance through multiple mechanisms: it thermodynamically and kinetically reduces energy barriers for intermediate hydrogenation, optimizes interfacial water orientation, and facilitates proton transfer. These integrated effects collectively enable the outstanding performance of the activated Pd‐CoS_2_ catalyst even at the ampere level.

## 3 Conclusion

Our study demonstrates that the in situ electrochemical reconstruction of a Pd‐CoS_2_ pre‐catalyst creates an isolated Pd‐doped metallic Co architecture, which serves as a superlative electrocatalyst for nitrate‐to‐ammonia conversion. Its ability to maintain over 98.0% ammonia selectivity at an industrial‐scale partial current density exceeding ‒1.4 A cm^−2^, coupled with exceptional long‐term stability, surpassing most reported NO_3_RR catalysts. The exceptional performance originates from a synergistic dual modulation mechanism, as uncovered by our integrated experimental and theoretical analyses. First, at the atomic level, Pd doping effectively tailors the electronic structure of Co sites, which optimizes the adsorption energetics of key nitrogenous intermediates and significantly reduces the kinetic barrier of the rate‐determining *NO hydrogenation step. Second, and more critically, the incorporated Pd atoms induce a comprehensive reconstruction of the interfacial microenvironment. In situ Raman spectroscopy reveals that Pd incorporation strengthens the local electric field and enriches hydrated K^+^ ions near the electrode surface. These cations, in closer proximity to the electrode surface, help stabilize negatively charged intermediates. Concurrently, a dynamic, hydrogen‐down‐oriented water network is established, as evidenced by AIMD simulations, which facilitates rapid proton transfer—a crucial factor for sustaining high reaction rates under large current conditions.

This mechanistic picture stands in instructive contrast to the role of Pd in our previously reported Cu‐based system. While Pd incorporation universally enhances NO_3_RR performance in both hosts by modulating the interfacial microenvironment, the specific manifestations and outcomes are host‐dependent. In the Cu matrix, Pd primarily serves to attenuate an overly strong intrinsic electric field, which otherwise leads to the excessive stabilization of *NO_3_ and a rigid interfacial water structure. The weakening of the field by Pd enhances hydrogen‐bond network connectivity, thereby facilitating proton transfer. In stark contrast, the Co matrix features a relatively weak intrinsic electric field and a dynamically sluggish interface. Here, Pd incorporation amplifies the local field, which drives cation enrichment and transforms the interfacial water into a more mobile and responsive state. This comparison underscores that Pd's function is not monolithic but is exquisitely tailored by the intrinsic properties of the host catalyst, enabling a precise correction of its specific interfacial deficiencies.

This work highlights that surpassing the activity‐selectivity‐stability trade‐off in high‐rate electrocatalysis requires moving beyond conventional electronic structure engineering. The critical synergy between atomic‐site optimization and deliberate interfacial microenvironment modulation emerges as a powerful and generalizable design principle. We anticipate that this dual‐regulation strategy will prove instrumental in advancing other multi‐step electrocatalytic transformations, such as CO_2_ and N_2_ reduction, where proton‐coupled electron transfer and intermediate stabilization at high fluxes are similarly paramount.

## Author Contributions

**Qun He**: conceptualization, investigation, validation, writing – original draft. **Chuanqiang Wu**: data curation, investigation. **Zhangsheng Shi**: formal analysis, investigation. **Dongxue Yu**: investigation. **Wei Jiang**: investigation. **Li Song**: validation, resources, investigation. **Xin Wang**: conceptualization, writing – review and editing, project administration, resources, supervision, formal analysis.

## Conflicts of Interest

The authors declare no conflicts of interest.

## Supporting information




**Supporting File**: anie72729‐sup‐0001‐SuppMat.docx.

## Data Availability

The data that support the findings of this study are available from the corresponding author upon reasonable request.

## References

[1] M. L. Sun , H. Y. Wang , Y. Feng , J. T. Ren , L. Wang , and Z. Y. Yuan , “Electrodegradation of Nitrogenous Pollutants in Sewage: From Reaction Fundamentals to Energy Valorization Applications,” Chemical Society Reviews 53 (2024): 11908–11966, 10.1039/D4CS00517A. 39498737

[2] R. Leones , “From Nitrate to Ammonia Using High‐Entropy Single‐Atom Nanocages,” Nature Reviews Materials 9 (2024): 676, 10.1038/s41578-024-00724-8.

[3] E. Abascal , L. Gómez‐Coma , I. Ortiz , and A. Ortiz , “Global Diagnosis of Nitrate Pollution in Groundwater and Review of Removal Technologies,” Science of the Total Environment 810 (2022): 152233, 10.1016/j.scitotenv.2021.152233. 34896495

[4] H. Xu , Y. Ma , J. Chen , W. Zhang , and J. Yang , “Electrocatalytic Reduction of Nitrate—A Step Towards a Sustainable Nitrogen Cycle,” Chemical Society Reviews 51 (2022): 2710–2758, 10.1039/D1CS00857A. 35274646

[5] V. Kyriakou , I. Garagounis , A. Vourros , E. Vasileiou , and M. Stoukides , “An Electrochemical Haber‐Bosch Process,” Joule 4 (2020): 142–158, 10.1016/j.joule.2019.10.006.

[6] Z. Wu , Y. Song , Y. Liu , W. Luo , W. Li , and J. Yang , “Electrocatalytic Nitrate Reduction: Selectivity at the Crossroads Between Ammonia and Nitrogen,” Chem Catalysis 3 (2023): 100786, 10.1016/j.checat.2023.100786.

[7] K. Zhang , Y. Liu , Z. Pan , et al., “Cu‐Based Catalysts for Electrocatalytic Nitrate Reduction to Ammonia: Fundamentals and Recent Advances,” EES Catalysis 2 (2024): 727–752, 10.1039/D4EY00002A.

[8] Y. Xiong , Y. Wang , J. Zhou , F. Liu , F. Hao , and Z. Fan , “Electrochemical Nitrate Reduction: Ammonia Synthesis and the Beyond,” Advanced Materials 36 (2024): 2304021, 10.1002/adma.202304021. 37294062

[9] X. Fan , D. Zhao , Z. Deng , et al., “Constructing Co@TiO_2_ Nanoarray Heterostructure With Schottky Contact for Selective Electrocatalytic Nitrate Reduction to Ammonia,” Small 19 (2023): 2208036, 10.1002/smll.202208036. 36717274

[10] X. Fan , C. Ma , D. Zhao , et al., “Unveiling Selective Nitrate Reduction to Ammonia With Co_3_O_4_ Nanosheets/TiO_2_ Nanobelt Heterostructure Catalyst,” Journal of Colloid & Interface Science 630 (2023): 714–720, 10.1016/j.jcis.2022.10.050. 36274406

[11] W. Chen , X. Yang , Z. Chen , et al., “Emerging Applications, Developments, Prospects, and Challenges of Electrochemical Nitrate‐to‐Ammonia Conversion,” Advanced Functional Materials 33 (2023): 2300512, 10.1002/adfm.202300512.

[12] Y. Qian , J. Lv , X. Liu , Z. Qi , and A. Wu , “Recent Advances in Electrocatalytic Conversion of Nitrates Into High‐Value Products,” Journal of Energy Chemistry 99 (2024): 50–65, 10.1016/j.jechem.2024.07.033.

[13] S. Zhang , R. Zhang , Y. Guo , and C. Zhi , “Ammonia Synthesis From Nitrate Reduction by the Modulation of a Built‐In Electric Field and External Stimuli,” EES Catalysis 3 (2025): 235–253, 10.1039/D4EY00245H.

[14] X. Gu , J. Zhang , S. Guo , et al., “Tiara Ni Clusters for Electrocatalytic Nitrate Reduction to Ammonia With 97% Faradaic Efficiency,” Journal of the American Chemical Society 147 (2025): 22785–22795, 10.1021/jacs.5c04950. 40538360 PMC12232310

[15] Q. Hu , C. Shang , X. Chen , et al., “Subnanometric Nickel Phosphide Heteroclusters With Highly Active Ni^δ+^ –P^δ−^ Pairs for Nitrate Reduction Toward Ammonia,” Journal of the American Chemical Society 147 (2025): 12228–12238, 10.1021/jacs.5c01455. 40138702

[16] W. Ye , Y. Yao , X. Wei , et al., “Continuous Intermediates Spillover Boosts Electrochemical Nitrate Conversion to Ammonia Over Dual Single‐Atom Alloy,” Angewandte Chemie International Edition 64 (2025): e202509303, 10.1002/anie.202509303. 40474588

[17] J. Wang , J. Cai , K. X. Ren , et al., “Stepwise Structural Evolution Toward Robust Carboranealkynyl‐Protected Copper Nanocluster Catalysts for Nitrate Electroreduction,” Science Advances 10 (2024): eadn7556, 10.1126/sciadv.adn7556. 38691609 PMC11062576

[18] Z. Shen , G. Chen , X. Cheng , et al., “Self‐Enhanced Localized Alkalinity at the Encapsulated Cu Catalyst for Superb Electrocatalytic Nitrate/Nitrite Reduction to NH_3_ in Neutral Electrolyte,” Science Advances 10 (2024): eadm9325, 10.1126/sciadv.adm9325. 38985876 PMC11235175

[19] Z. Chen , Y. Zhao , H. Huang , et al., “Isolated Copper Atoms Boost *NO_3_ Adsorption and Active Hydrogen Retention Over Zinc Oxide for Ammonia Electrosynthesis at Ampere‐Level Current Densities,” Journal of the American Chemical Society 147 (2025): 18737–18746, 10.1021/jacs.5c01863. 40400257

[20] Z. Li , Z. Shi , Y. Ou , et al., “Pulsed Electrocatalysis Driven Efficient Ammonia Synthesis by Facilitating *NOOH Formation and Balancing *H Supply,” Angewandte Chemie International Edition 64 (2025): e202510287, 10.1002/anie.202510287. 40708400

[21] Z. Li , C. Yang , B. Xu , L. Yao , W. Zhu , and Y. Cui , “Electrochemically Nitrate Remediation by Single‐Atom Catalysts: Advances, Mechanisms, and Prospects,” Energy Materials 4 (2024): 400046, 10.20517/energymater.2023.147.

[22] R. Zhang , X. Ma , S. Zhang , Q. Li , Y. Zhao , and C. Zhi , “Alloy Catalysts for Electrochemical Nitrate Reduction to Ammonia,” ChemElectroChem 12 (2025): e202400499, 10.1002/celc.202400499.

[23] Y. Yang , Y. Sun , Y. Wang , et al., “Self‐Triggering a Locally Alkaline Microenvironment of Co_4_Fe_6_ for Highly Efficient Neutral Ammonia Electrosynthesis,” Journal of the American Chemical Society 147 (2025): 8893–8905, 10.1021/jacs.5c00688. 40019172

[24] S. Sun , C. Dai , P. Zhao , et al., “Spin‐Related Cu‐Co Pair to Increase Electrochemical Ammonia Generation on High‐Entropy Oxides,” Nature Communications 15 (2024): 260, 10.1038/s41467-023-44587-z. PMC10766993 38177119

[25] R. Zhao , Q. Yan , L. Yu , et al., “A Bi‐Co Corridor Construction Effectively Improving the Selectivity of Electrocatalytic Nitrate Reduction Toward Ammonia by Nearly 100%,” Advanced Materials 35 (2023): 2306633, 10.1002/adma.202306633. 37736698

[26] K. Yang , S. H. Han , C. Cheng , C. Guo , T. Li , and Y. Yu , “Unveiling the Reaction Mechanism of Nitrate Reduction to Ammonia Over Cobalt‐Based Electrocatalysts,” Journal of the American Chemical Society 146 (2024): 12976–12983, 10.1021/jacs.3c13517. 38567925

[27] Z. N. Zhang , Q. L. Hong , X. H. Wang , H. Huang , S. N. Li , and Y. Chen , “Au Nanowires Decorated Ultrathin Co_3_O_4_ Nanosheets Toward Light‐Enhanced Nitrate Electroreduction,” Small 19 (2023): 2300530, 10.1002/smll.202300530. 36971299

[28] F. Zhou , B. Lv , Y. Zhang , Y. Wu , Y. Wang , and W. Luo , “Carbon‐Constrained CoRu Bimetallic Catalysts Enabling Efficient Electrocatalytic Reduction of Nitrate to Ammonia,” Journal of Power Sources 657 (2025): 238222, 10.1016/j.jpowsour.2025.238222.

[29] X. Zhu , Y. C. Wang , K. Qu , et al., “Modulating Ru‐Co Bond Lengths in Ru_1_Co Single‐atom Alloys Through Crystal Phase Engineering for Electrocatalytic Nitrate‐to‐Ammonia Conversion,” Nature Communications 16 (2025): 5742, 10.1038/s41467-025-61232-z. PMC12214835 40593900

[30] R. Y. Zhou , S. Zheng , X. Liu , et al., “Elevating Nitrate Reduction Through the Mastery of Hierarchical Hydrogen‐Bond Networks,” Journal of the American Chemical Society 147 (2025): 20504–20511, 10.1021/jacs.5c02540. 40481782

[31] P. Muthukumar , Z. Ullah , X. Zhang , et al., “Unlocking a Water Coordination Environment in Co‐Based Metal–Organic Frameworks for Advanced Nitrate‐to‐Ammonia Electroreduction,” Journal of the American Chemical Society 147 (2025): 29949–29960, 10.1021/jacs.5c07066. 40765371 PMC12755198

[32] R. Zhao , Q. Wang , Y. Yao , et al., “Pd Single Atoms Guided Proton Transfer Along an Interfacial Hydrogen Bond Network for Efficient Electrochemical Hydrogenation,” Science Advances 11 (2025): eadu1602, 10.1126/sciadv.adu1602. 40779631 PMC12333693

[33] Q. He , Z. Shi , D. Yu , et al., “Dynamic Reconstruction and Microenvironment Modulation of a Pd‐Doped CuS Electrocatalyst for Nearly Unity‐Efficiency Ammonia Electrosynthesis From Nitrate,” Journal of the American Chemical Society 147 (2025): 43067–43076, 10.1021/jacs.5c16232. 41197119 PMC12817242

[34] X. Xiao , S. Shen , L. Zhang , et al., “Construction of Cobalt Molybdenum Diselenide Three‐Phase Heterojunctions for Electrocatalytic Hydrogen Evolution in Acid Medium,” Chemistry ‐ An Asian Journal 18 (2023): e202201182, 10.1002/asia.202201182. 36465037

[35] D. Escalera‐López , Y. Niu , S. J. Park , et al., “Hydrogen Evolution Enhancement of Ultra‐Low Loading, Size‐Selected Molybdenum Sulfide Nanoclusters by Sulfur Enrichment,” Applied Catalysis B: Environment and Energy 235 (2018): 84–91, 10.1016/j.apcatb.2018.04.068.

[36] S. Qiao , H. Shou , W. Xu , et al., “Regulating and Identifying the Structures of PdAu Alloys With Splendid Oxygen Reduction Activity for Rechargeable Zinc–air Batteries,” Energy & Environmental Science 16 (2023): 5842–5851, 10.1039/D3EE02719H.

[37] O. Q. Carvalho , R. Marks , H. K. K. Nguyen , et al., “Role of Electronic Structure on Nitrate Reduction to Ammonium: A Periodic Journey,” Journal of the American Chemical Society 144 (2022): 14809–14818, 10.1021/jacs.2c05673. 35926171

[38] E. Murphy , B. Sun , M. Rüscher , et al., “Synergizing Fe_2_O_3_ Nanoparticles on Single Atom Fe‐N‐C for Nitrate Reduction to Ammonia at Industrial Current Densities,” Advanced Materials 36 (2024): 2401133, 10.1002/adma.202401133. 38619914

[39] X. Shi , M. Xie , K. Yang , et al., “Synergistic Effect of Ni/Ni(OH)_2_ Core‐Shell Catalyst Boosts Tandem Nitrate Reduction for Ampere‐Level Ammonia Production,” Angewandte Chemie International Edition 63 (2024): e202406750, 10.1002/anie.202406750. 38651747

[40] J. Zhang , T. Quast , B. Eid , et al., “In‐Situ Electrochemical Reconstruction and Modulation of Adsorbed Hydrogen Coverage in Cobalt/Ruthenium‐Based Catalyst Boost Electroreduction of Nitrate to Ammonia,” Nature Communications 15 (2024): 8583, 10.1038/s41467-024-52780-x. PMC11450097 39362855

[41] J. Li , W. Yu , H. Yuan , et al., “Lattice Hydrogen Transfer in Titanium Hydride Enhances Electrocatalytic Nitrate to Ammonia Conversion,” Nature Communications 15 (2024): 9499, 10.1038/s41467-024-53833-x. PMC11532501 39489774

[42] J. Kang , Y. Xiao , L. Li , et al., “Ternary Synergy in Layered Double Hydroxides for Efficient and Stable Nitrate Reduction,” Advanced Functional Materials 35 (2025): 2507619, 10.1002/adfm.202507619.

[43] Y. Wan , Y. Tang , Y. Zuo , et al., “Interfacial Hydrogen‐Bond Modulation of Dynamic Catalysts for Nitrate Electroreduction to Ammonia,” Energy & Environmental Science 18 (2025): 7460–7469, 10.1039/D5EE00597C.

[44] L. Qiao , A. Zhu , D. Liu , et al., “In Situ Reconstructed Cu/β‐Co(OH)_2_ Tandem Catalyst for Enhanced Nitrate Electroreduction to Ammonia in Ampere‐Level,” Advanced Energy Materials 14 (2024): 2402805, 10.1002/aenm.202402805.

[45] S. Zhang , Y. Liu , Y. Ding , et al., “Rational Ligand Design of Conjugated Coordination Polymers for Efficient and Selective Nitrate Electroreduction to Ammonia,” Advanced Materials 37 (2025): 2418681, 10.1002/adma.202418681. 40285545

[46] H. He , H. Yao , L. Sun , Y. Yang , Z. A. Qiao , and B. Liu , “High‐Entropy Effect of Mesoporous Metal Oxides Promotes Tandem Catalysis for Efficient Ammonia Electrosynthesis From Nitrate,” Advanced Materials 37 (2025): e08982, 10.1002/adma.202508982. 40714763

[47] Y. Zuo , M. Sun , T. Li , et al., “Capturing Copper Single Atom in Proton Donor Stimulated O‐End Nitrate Reduction,” Advanced Materials 37 (2025): 2415632, 10.1002/adma.202415632. 39967378 PMC11938000

[48] C. Cai , K. Liu , L. Zhang , et al., “Atomically Local Electric Field Induced Interface Water Reorientation for Alkaline Hydrogen Evolution Reaction,” Angewandte Chemie International Edition 62 (2023): e202300873, 10.1002/anie.202300873. 36883799

[49] J. Guo and P. Chen , “Interplay of Alkali, Transition Metals, Nitrogen, and Hydrogen in Ammonia Synthesis and Decomposition Reactions,” Accounts of Chemical Research 54 (2021): 2434–2444, 10.1021/acs.accounts.1c00076. 33913703

