# Supplementary material for: Palladium‐Doping‐Enabled Interface Synergy for Superb Ampere Level Ammonia Electrosynthesis From Nitrate
Source: Angew Chem Int Ed Engl. 2026 May 18;65(28):e2794664. doi: 10.1002/anie.2794664 (PMC13340510; doi:10.1002/anie.2794664)
Supplement: Supplementary file 1 — Supporting File: anie72729‐sup‐0001‐SuppMat.docx. [file ANIE-65-e2794664-s001.docx]

Supplementary Information

for

**Palladium-doping-enabled Interface Synergy for Superb Ampere Level Ammonia Electrosynthesis from Nitrate**

Qun He^†[a]^, Chuanqiang Wu^†[b]^, Zhangsheng Shi^[a]^, Dongxue Yu^[a]^, Wei Jiang^[c]^, Li Song*^[c]^, and Xin Wang*^[a]^

1. Dr. Q. He, Mr. Z. Shi, Ms. D. Yu, Prof. X. Wang

Department of Chemistry

City University of Hong Kong

Kowloon 999077, China

E-mail: wang.xin@cityu.edu.hk

1. Prof. C. Wu

State Information Materials and Intelligent Sensing Laboratory of Anhui Province, Key Laboratory of Structure and Functional Regulation of Hybrid Materials of Ministry of Education, Institutes of Physical Science and Information Technology

Anhui University

Hefei 230601, China

1. Dr. W. Jiang, Prof. L. Song

National Synchrotron Radiation Laboratory

University of Science and Technology of China

Hefei 230029, China

E-mail: song2012@ustc.edu.cn

**Experimental Section**

**Synthesis of catalysts.** All chemicals were of analytical grade and used without further purification. The typical synthesis procedure for Pd-CoS_2_ was as follows: 1.0 mmol CoCl_2_·6H_2_O and 0.012 mmol K_2_PdCl_4_ were dissolved in 4.5 mL of ethanol to form a homogeneous solution. Then, 1.3 mL of (±)-propylene oxide was added dropwise under stirring. The resulting precursor was collected by centrifugation and vacuum-dried at 60 °C. Subsequently, 50 mg of the dried precursor was placed in a quartz boat and covered with 1.0 g of sublimed sulfur. The boat was then annealed at 400°C for 3 hours in an argon atmosphere with a heating rate of 2°C min^-1^. After cooling, the product was thoroughly ground and washed sequentially with carbon disulfide, deionized water, and ethanol (three times each). Finally, the sample was vacuum-dried again at 60 °C to obtain Pd-CoS_2_. The synthesis of CoS_2_ and Pd-doped CoS_2_ with various Pd contents followed a similar process to that of Pd_-_CoS_2_, with the exception of the addition of K_2_PdCl_4_ or the usage amount change of K_2_PdCl_4_ to 0.004, 0.008, and 0.018 mmol.

**Materials characterization.** The structural characterization of all samples began with XRD performed on a Rigaku SmartLab X-ray diffractometer. Morphological and structural analyses were further conducted using TEM and HRTEM on a JEM-2100F field emission electron microscopy operated at 200 kV. Atomic-resolution imaging was carried out via HAADF-STEM on a JEOL JEM-ARF200F instrument (200 kV) equipped with a spherical aberration corrector, coupled with elemental mapping. XPS measurements were conducted on a Thermo ESCALAB 250 spectrometer using a monochromatic Al K_α_ source (1486.6 eV). ¹H NMR spectra were acquired on a Bruker AVANCE III 400 MHz spectrometer. The Co L_2,3_-edge XANES spectra were measured at the XMCD beamline of the Hefei Light Source (HLS), while the Co K-edge and Pd K-edge X-ray absorption fine structure (XAFS) spectra were collected at the 1W1B beamline of the Beijing Synchrotron Radiation Facility (BSRF) and the 14W1 beamline of the Shanghai Synchrotron Radiation Facility (SSRF), respectively.

**In-situ Raman experiments.** In-situ Raman spectra were acquired on a Horiba XploRA Plus confocal Raman microscope equipped with a 1200 grooves/mm grating. A 638 nm laser with maximum output power of 30 mW was used as the excitation source. The spectrometer was calibrated using a silicon wafer standard (peak at 520 cm^-1^), and spectral were recorded with a CCD detector (1024 × 256 pixels). For the Raman measurements presented in Figures 1e and 1f, the pristine catalysts were used. The working electrode was first subjected to chronoamperometry at specified potentials for 15 minutes, after which the spectra were acquired. For the Raman experiments in Figures 3d and 3e, the catalysts were electrochemically activated by 100 CV cycles from +0.5 to -0.8 V vs. RHE at a scan rate of 100 mV s^-1^ to reach a stable state. All electrochemical measurements were carried out in an argon-saturated 1.0 M KOH electrolyte containing 0.5 M KNO_3_.

**In-situ DEMS experiments.** The in-situ DEMS measurements were performed with a LingLu QAS100 system (Linglu, China) using a three-electrode electrochemical cell. The cell consisted of a catalyst-coated working electrode, a Pt wire counter electrode, and an Ag/AgCl reference electrode. The electrolyte used was 1.0 M KOH containing 0.5 M KNO_3_. Prior to measurement, the as-prepared catalysts were activated by CV. After the signal stabilized, chronoamperometry was carried out at selected potentials. Real-time mass signals corresponding to H_2_ (2), NH_2_ (16), NH_3_ (17), NO (30), NH_2_OH (33), and NO_2_ (46) were monitored during the reaction.

**Electrochemical measurements.** The electrochemical measurements were carried out using a MULTI AUTOLAB M204 electrochemical workstation equipped with a standard three-electrode system in an H-cell configuration. The system consists of a catalyst loaded carbon paper working electrode (0.3 cm^2^), a carbon rod counter electrode, and a Hg/HgO reference electrode (calibrated against the RHE). All experiments were conducted in an argon-saturated electrolyte containing 1.0 M KOH and 0.5 M KNO_3_. Unless otherwise stated, all potentials reported in this work are referenced to the RHE and current densities are normalized to the geometric surface area of the electrode. The working electrode was prepared as follows: 2 mg of the catalyst sample was dispersed in a mixture of 0.40 mL deionized water, 0.55 mL isopropyl alcohol, and 0.05 mL of 5% Nafion solution. The mixture was sonicated for 1 hour to form a homogeneous ink. Then, 100 μL of the ink was drop-casted onto the carbon paper substrate and dried at room temperature. Prior to electrochemical testing, the catalysts were activated by 100 cycles of CV between +0.5 and -0.8 V vs. RHE at a scan rate of 100 mV s^-1^ to reach a stable state. Subsequently, CV was performed at a scan rate of 5 mV s^-1^ to analyze the electrochemical behavior. Chronoamperometry was conducted at potentials ranging from -0.7 to -1.3 V vs. RHE to evaluate catalytic performance. The reaction products were quantified using UV-Vis spectrophotometry and gas chromatography to determine selectivity. Catalyst stability was assessed via chronoamperometry at -1.1 V vs. RHE. Mass activity was calculated based on catalyst loading amounts.

**Theoretical calculations and AIMD simulations.** Spin-polarized calculations based on the density functional theory (DFT) methods were performed by Ab initio Simulation Package (VASP) with the projector augmented wave (PAW) method.^[1]^ The generalized gradient approximation (GGA) of Perdew-Burke-Ernzerhof (PBE) function was utilized to describe the exchange-correlation interactions.^[2]^ A kinetic energy cutoff of 500 eV was used for all calculations. The convergence threshold was set as 10^-5^ eV for energy and 0.01 eV/Å for force. The smearing width for gaussian smearing was set as 0.10 eV. All surfaces based on a 4-layer 4 × 4 supercell of Co (111) termination were modeled. The bottom two layers were fixed as bulk regions, and everything else was allowed to relax. A vacuum layer of at least 20 Å was included along the z direction to avoid the interaction between adjacent images. A k-point mesh of 3 × 3 × 1 was used for both structural relaxation and self-consistent calculation. The zero damping DFT-D3 method of Grimme was applied for dispersion correction.^[3]^ VESTA was adopted to show the structures, and the VASP calculation data was dealt with the post-processing VASPKIT package.^[4]^ The transition state searches were conducted by combining the climbing image-nudged elastic band (CI-NEB) and dimer methods.^[5]^ Vibrational frequencies were analyzed to confirm the transition state with only one imaginary frequency. The Gibbs free energy changes were evaluated according to ΔG=ΔE+ΔZPE-TΔS, where ΔE is the DFT total energy difference, ΔZPE is the zero-point energy correction, and TΔS accounts for entropic contributions. Constrained ab initio molecular dynamics (AIMD) simulations with a slow-growth sampling approach were conducted to evaluate the kinetic process of water dissociation, along with the usage of a single k-point. A supercell consisting of 64 Co atoms, 30 water molecules, where one Co site was substituted by a Pd atom afterwards, was constructed to simulate the interfacial solvent/catalyst systems. The canonical ensemble condition (NVT) was imposed by a Nose-Hoover thermostat at a constant temperature of 300 K. The total simulation time was 20 ps to ensure the equilibration of interfacial systems and the timestep was set to 1fs. The post-processing for the spatial distribution of solvent molecules along the z direction was completed by VMD software.^[6]^

**Supplementary Figures and Table**





**Figure S1.** XRD patterns of Co_2_(OH)_3_Cl and Co_2_(OH)_3_Cl-Pd.


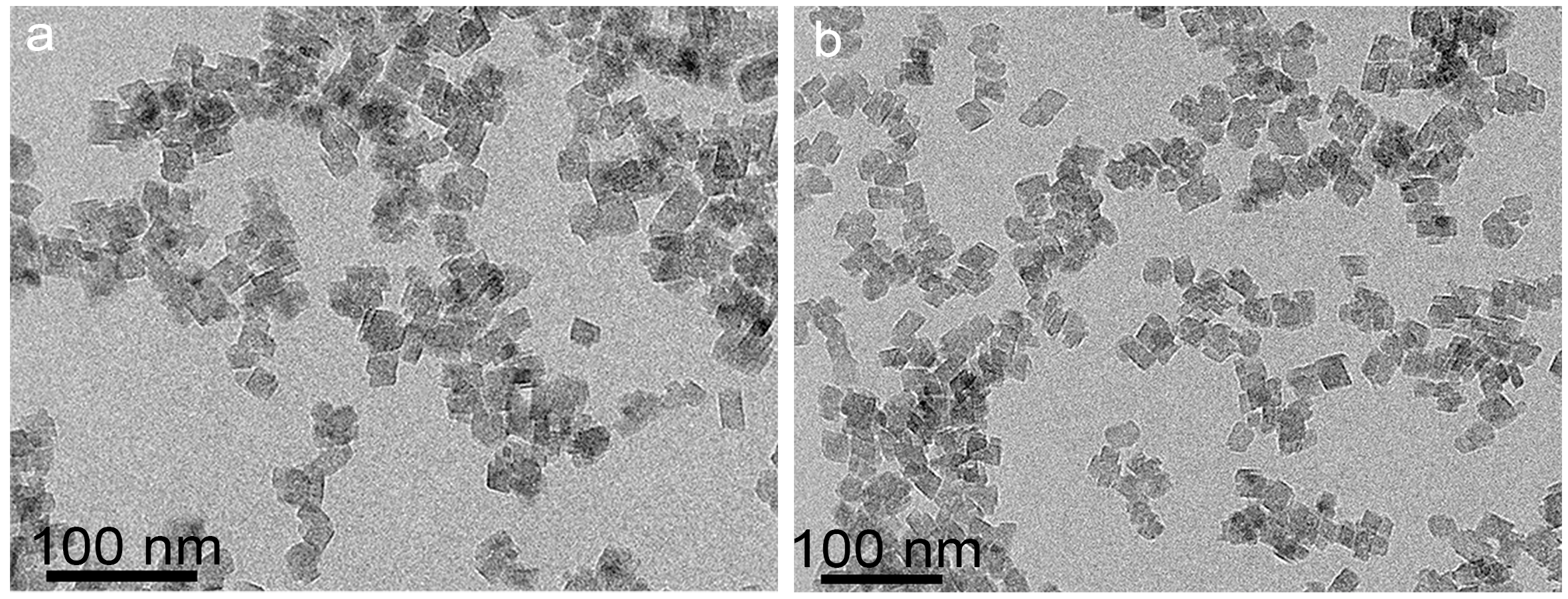


**Figure S2.** TEM images of (a) Co_2_(OH)_3_Cl and (b) Co_2_(OH)_3_Cl-Pd.


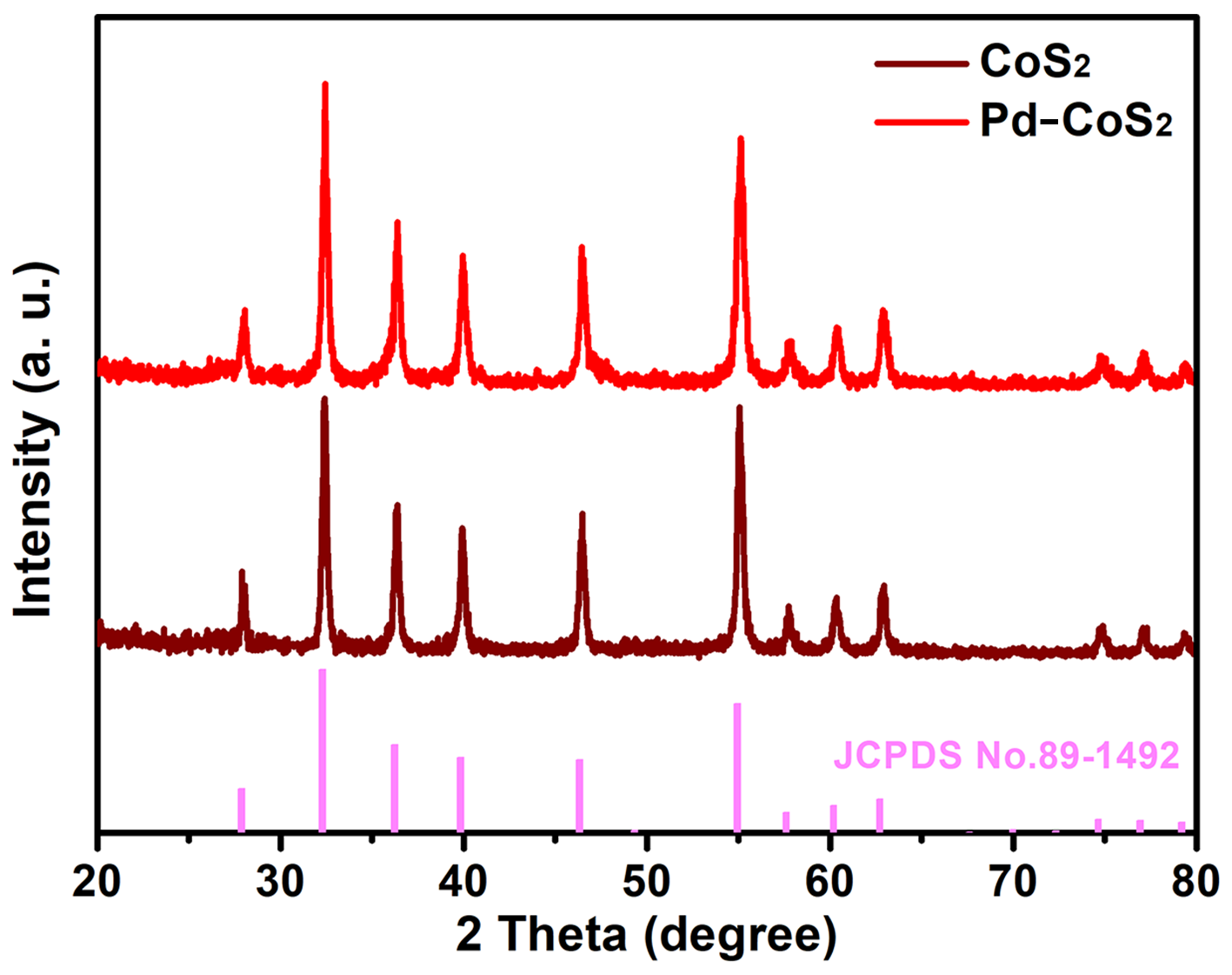


**Figure S3.** XRD patterns of CoS_2_ and Pd-CoS_2_.


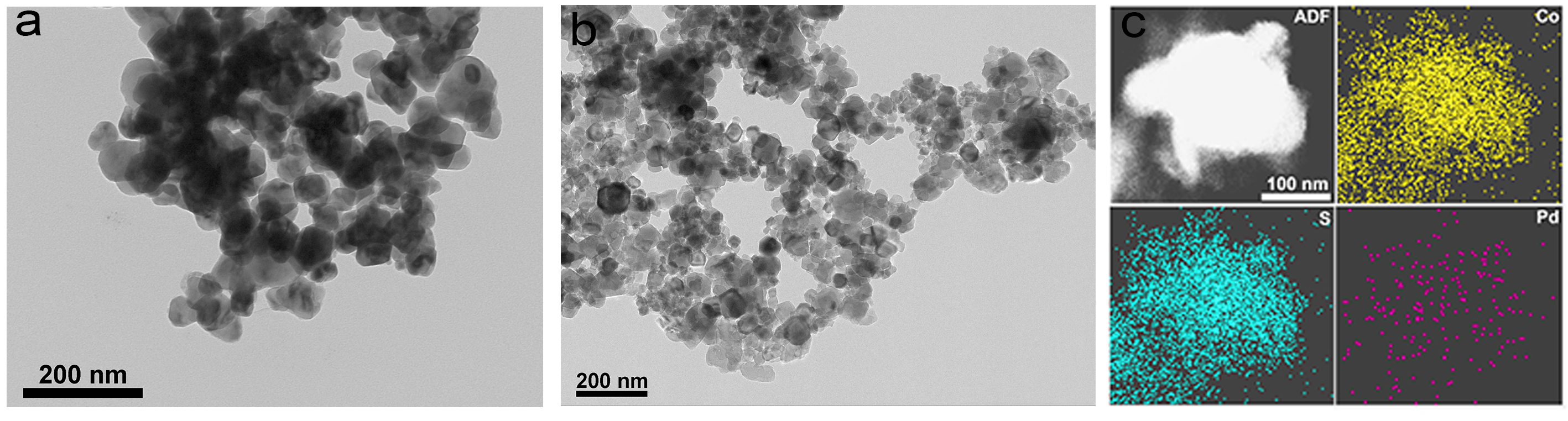


**Figure S4.** TEM images of (a) CoS_2_ and (b) Pd-CoS_2_. (c) Elemental mappings of Co, S, and Pd in Pd-CoS_2_.


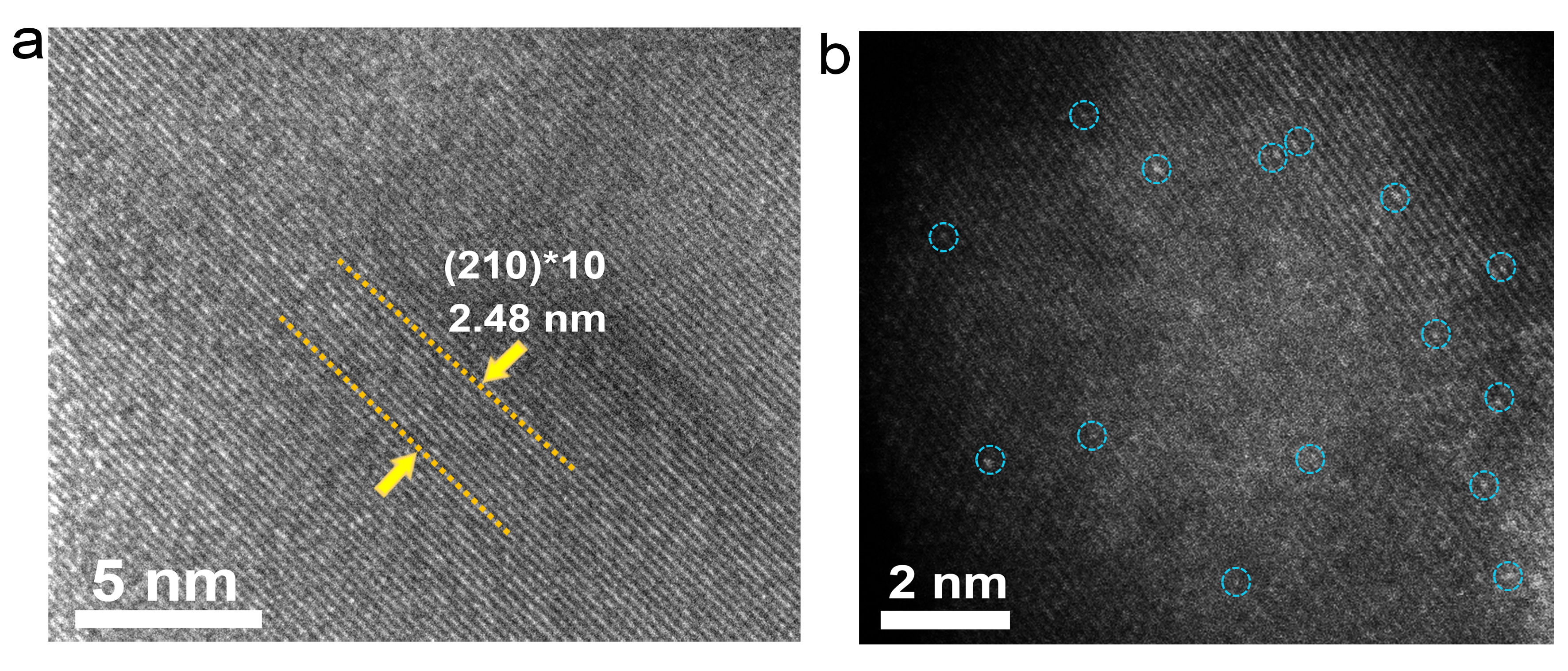


**Figure S5.** (a) HRTEM and (b) HAADF-STEM images of Pd-CoS_2_.


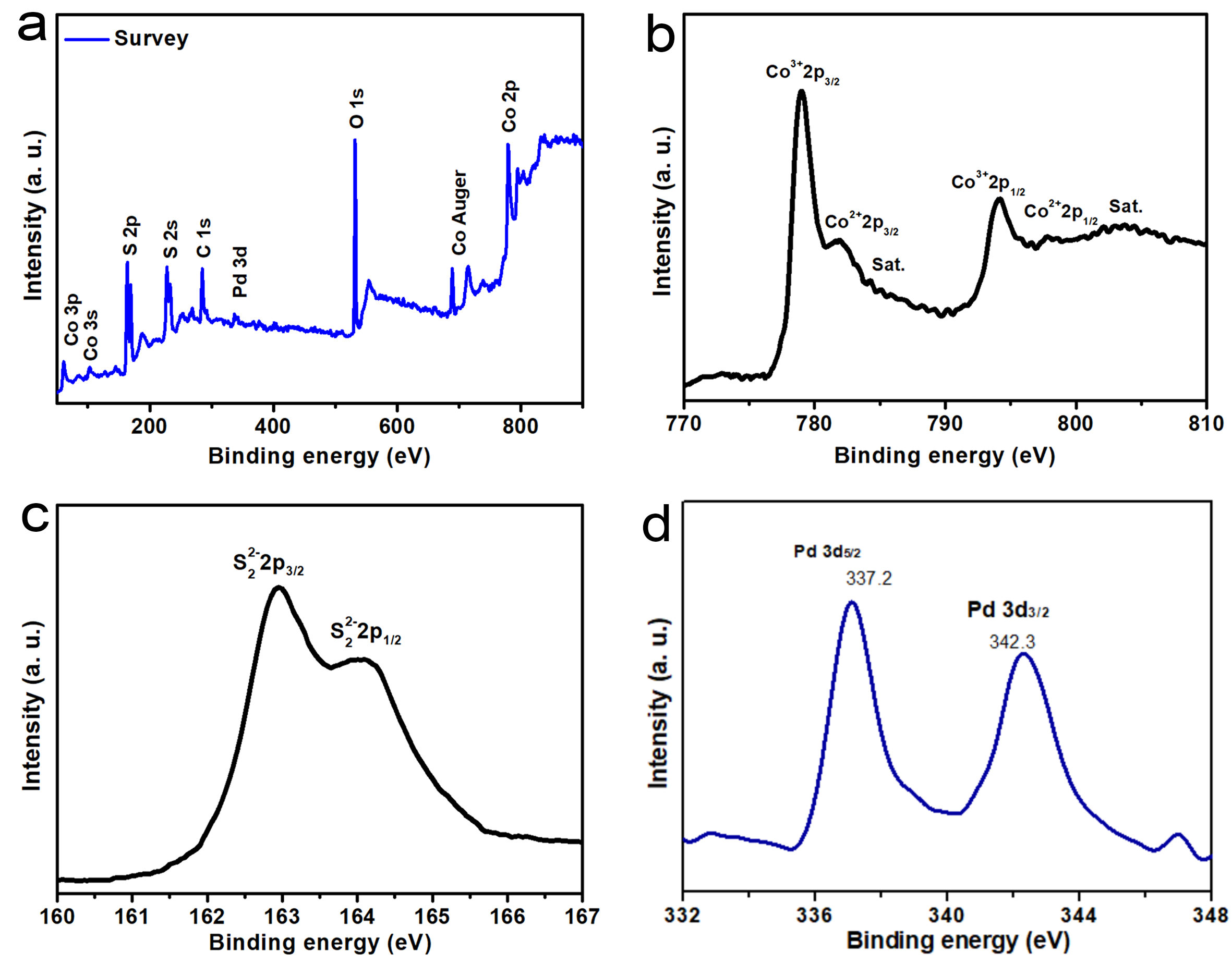


**Figure S6.** XPS (a) survey, high-resolution (b) Co 2p, (c) S 2p, and (d) Pd 3d spectra of Pd-CoS_2_.


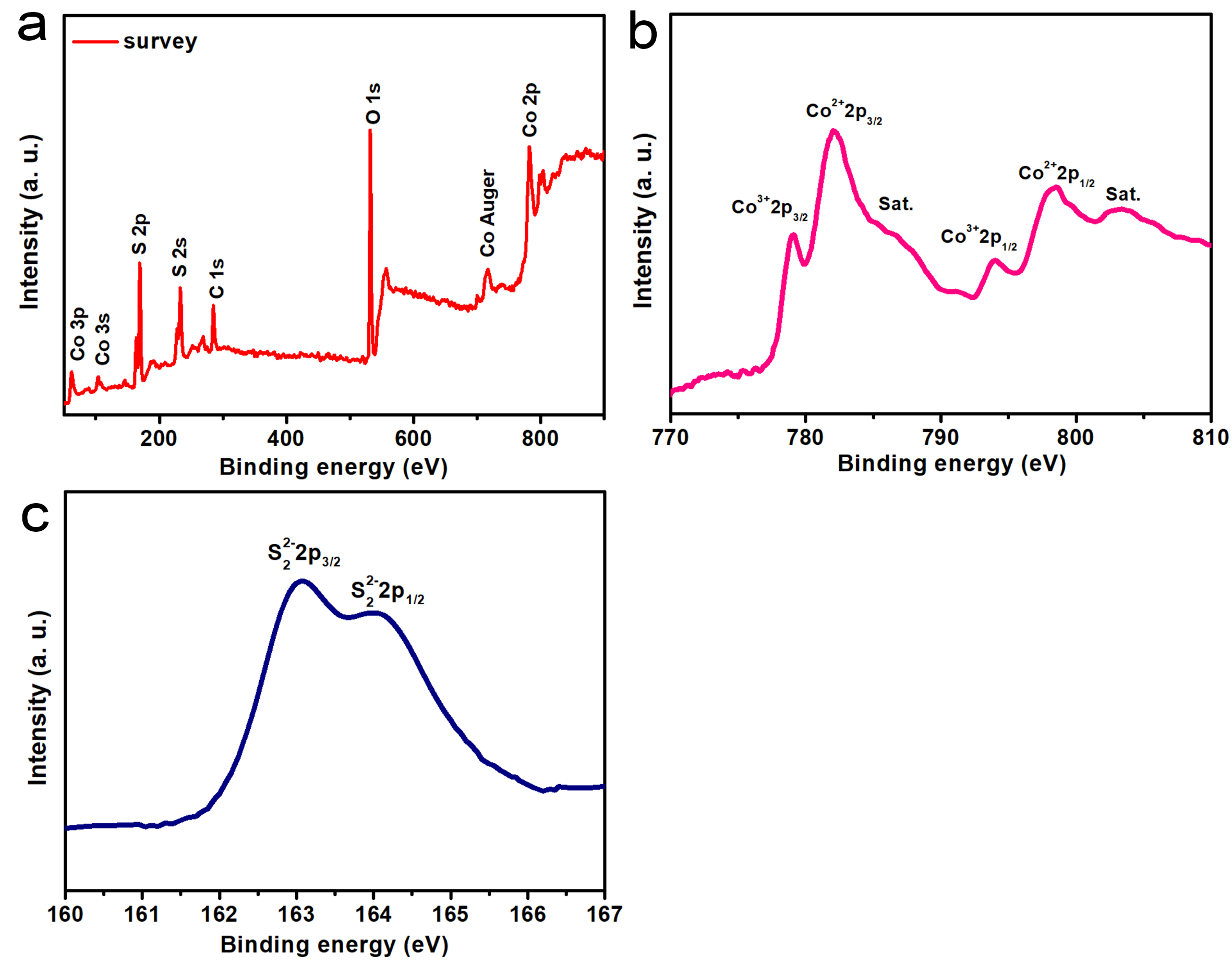


**Figure S7.** XPS (a) survey, high-resolution (b) Co 2p, and (c) S 2p spectra of CoS_2_.


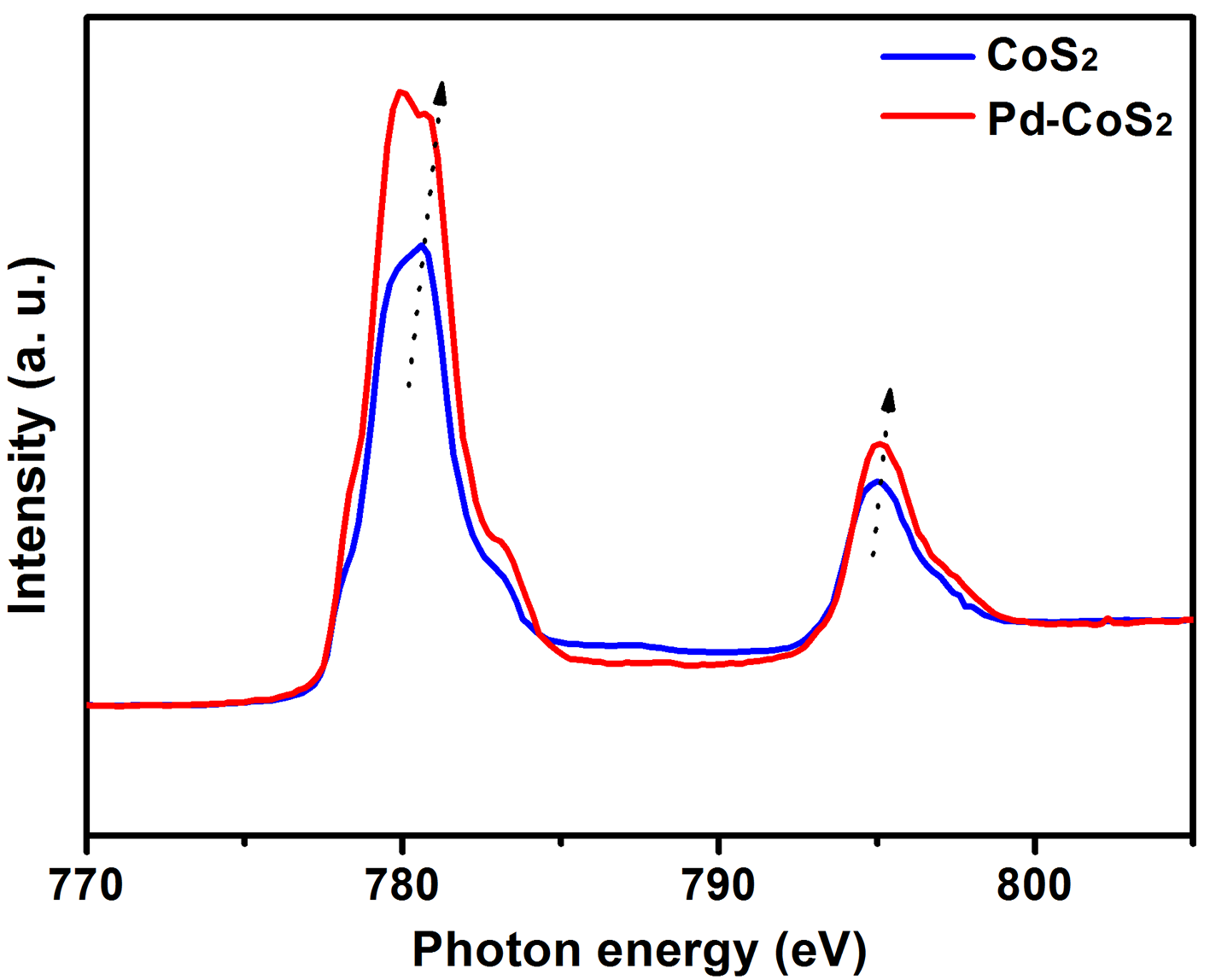


**Figure S8.** Co L_2,3_-edge XANES spectra of CoS_2_ and Pd-CoS_2_.


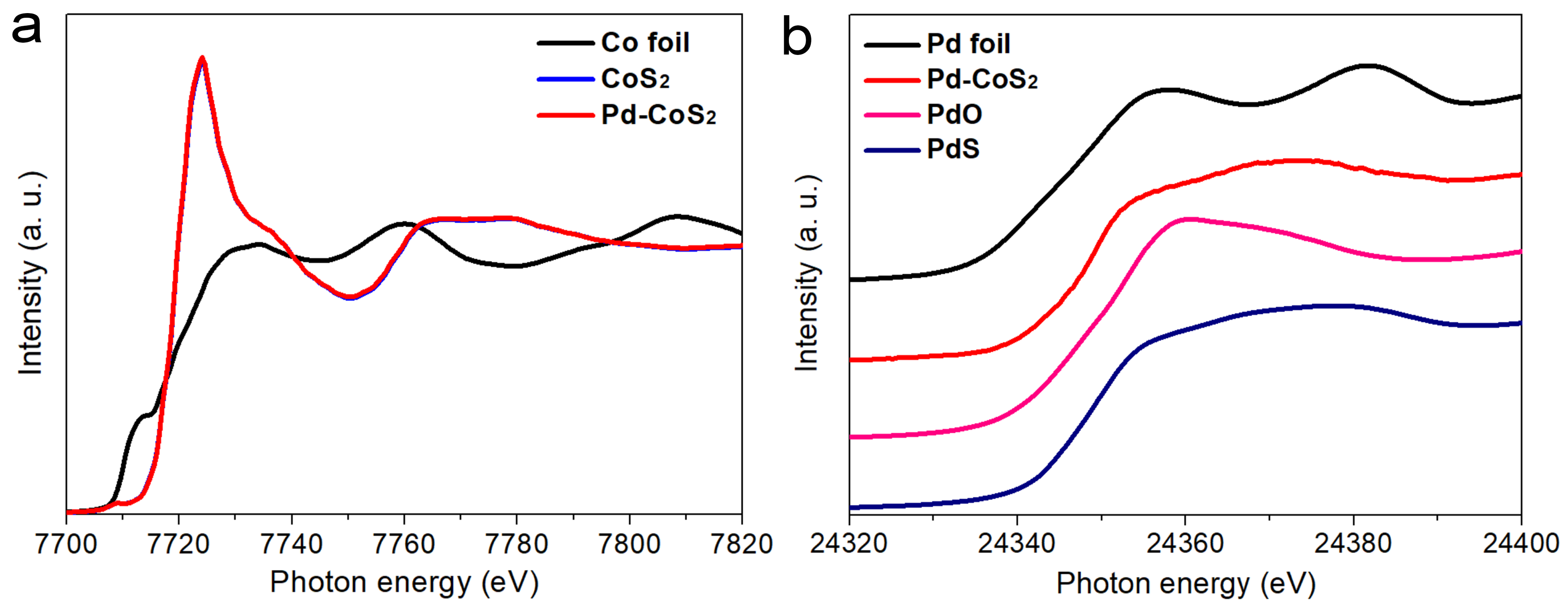


**Figure S9.** (a) Co K-edge XANES spectra of Co foil, Pd-CoS_2_, and CoS_2_. (b) Pd K-edge XANES spectra of Pd foil, Pd-CoS_2_, PdO, and PdS.


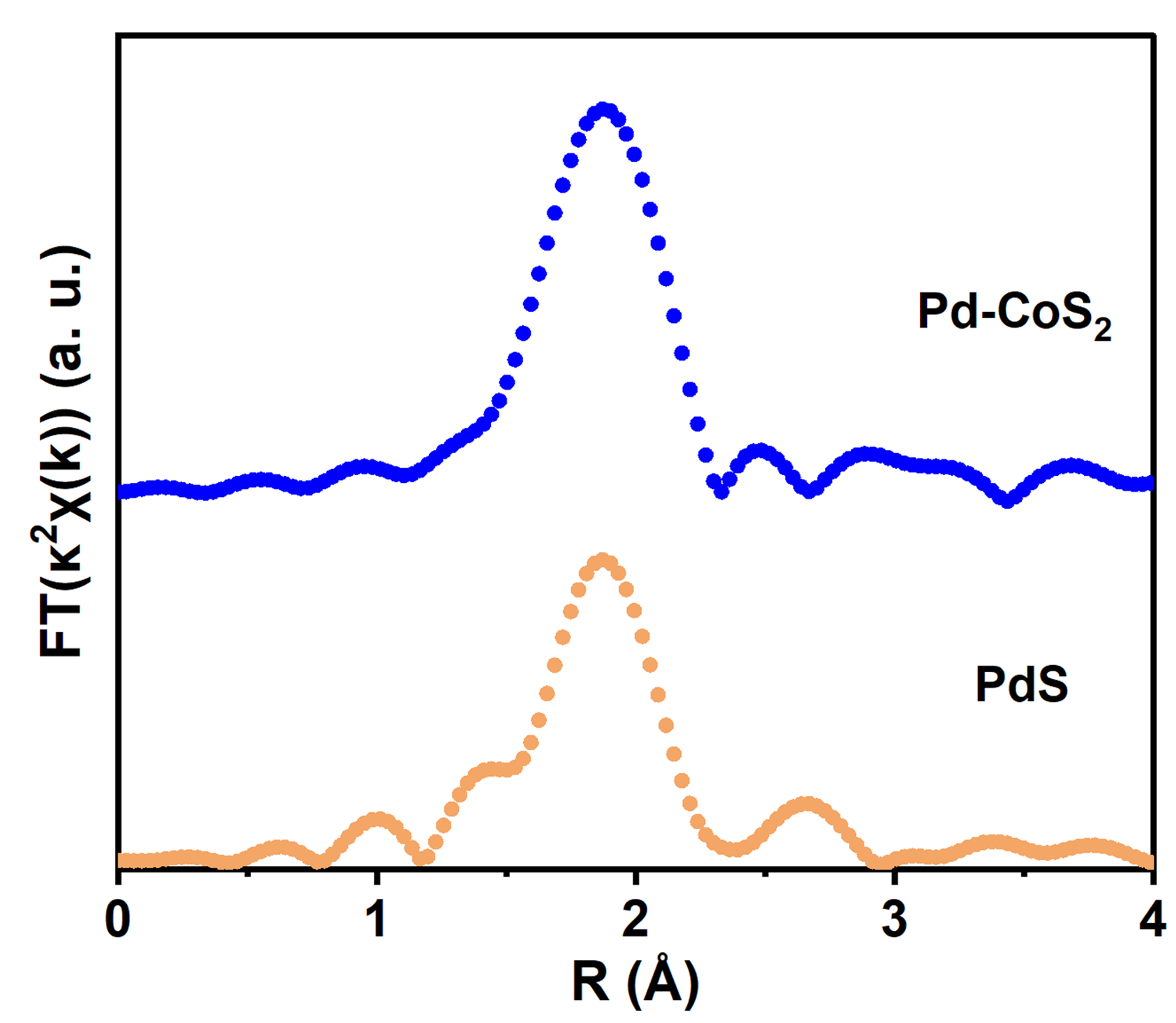


**Figure S10.** Pd K-edge FT-EXAFS spectra of Pd-CoS_2_ and PdS.


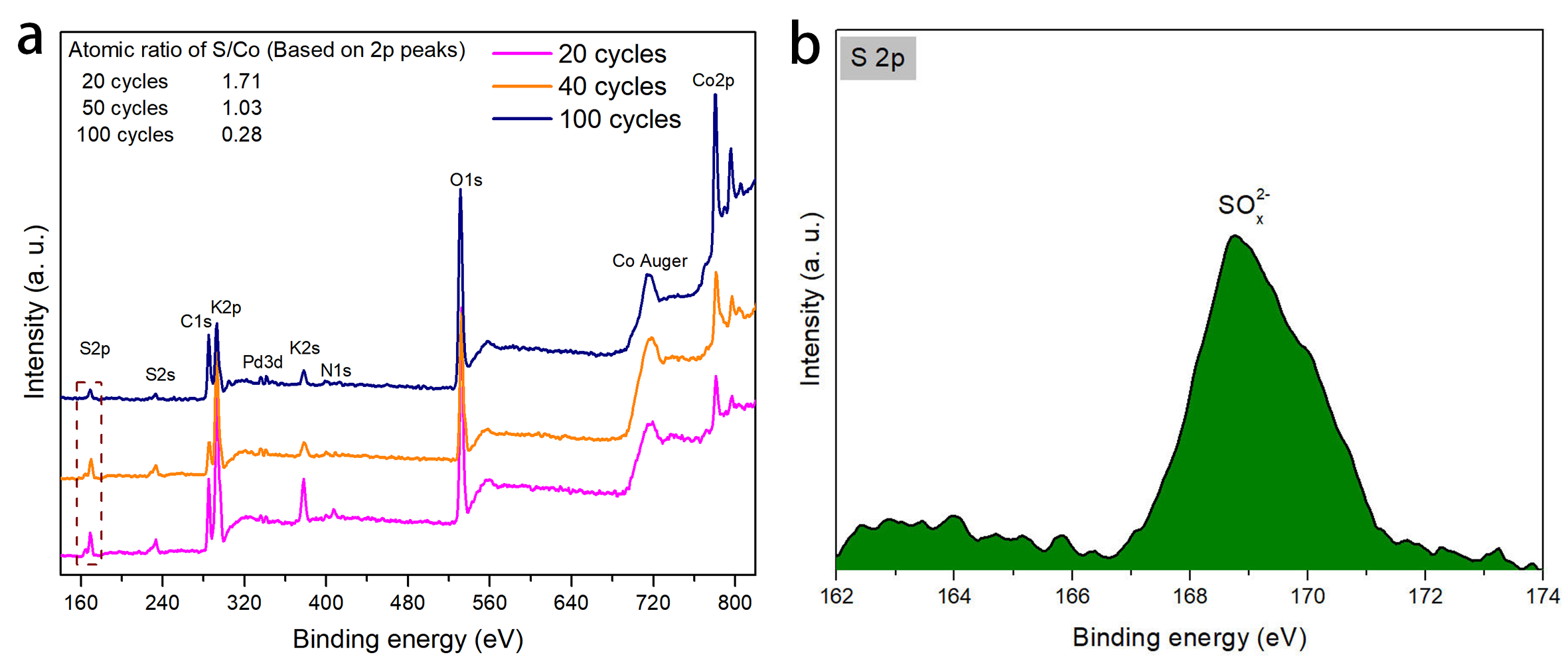


**Figure S11.** (a) XPS survey spectra of Pd-CoS_2_ after different CV cycles. (b) high-resolution S 2p spectrum after 100 cycles.

**Figure S12.** XRD pattern of Pd-CoS_2_ after long-term stability test. The catalyst was washed with water and stored under vacuum prior to measurement.


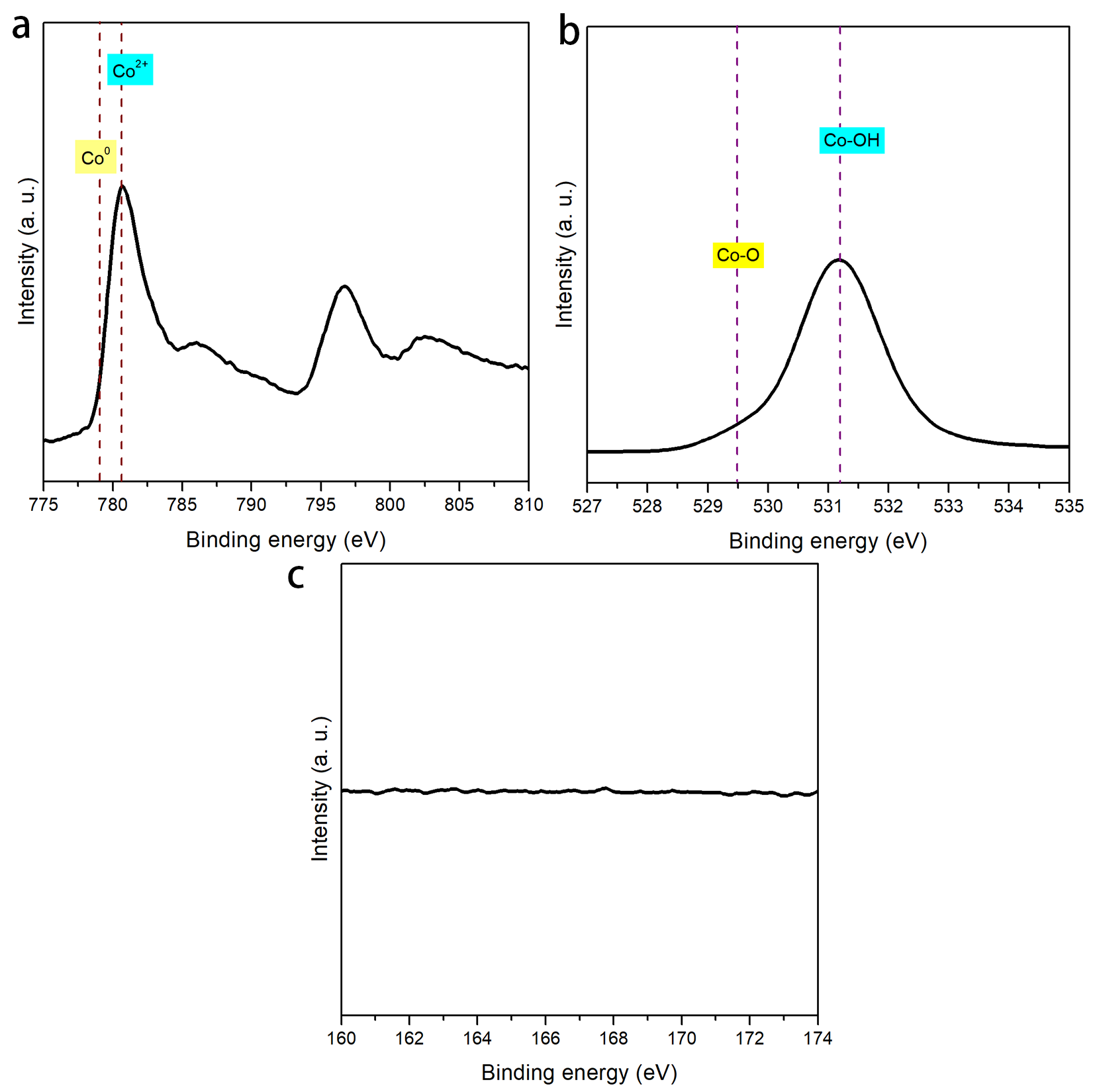


**Figure S13.** High-resolution XPS of (a) Co 2p, (b) O 1s, and (c) S 2p of post-reaction Pd-CoS_2_. The catalyst was washed with water and stored under vacuum prior to measurement.


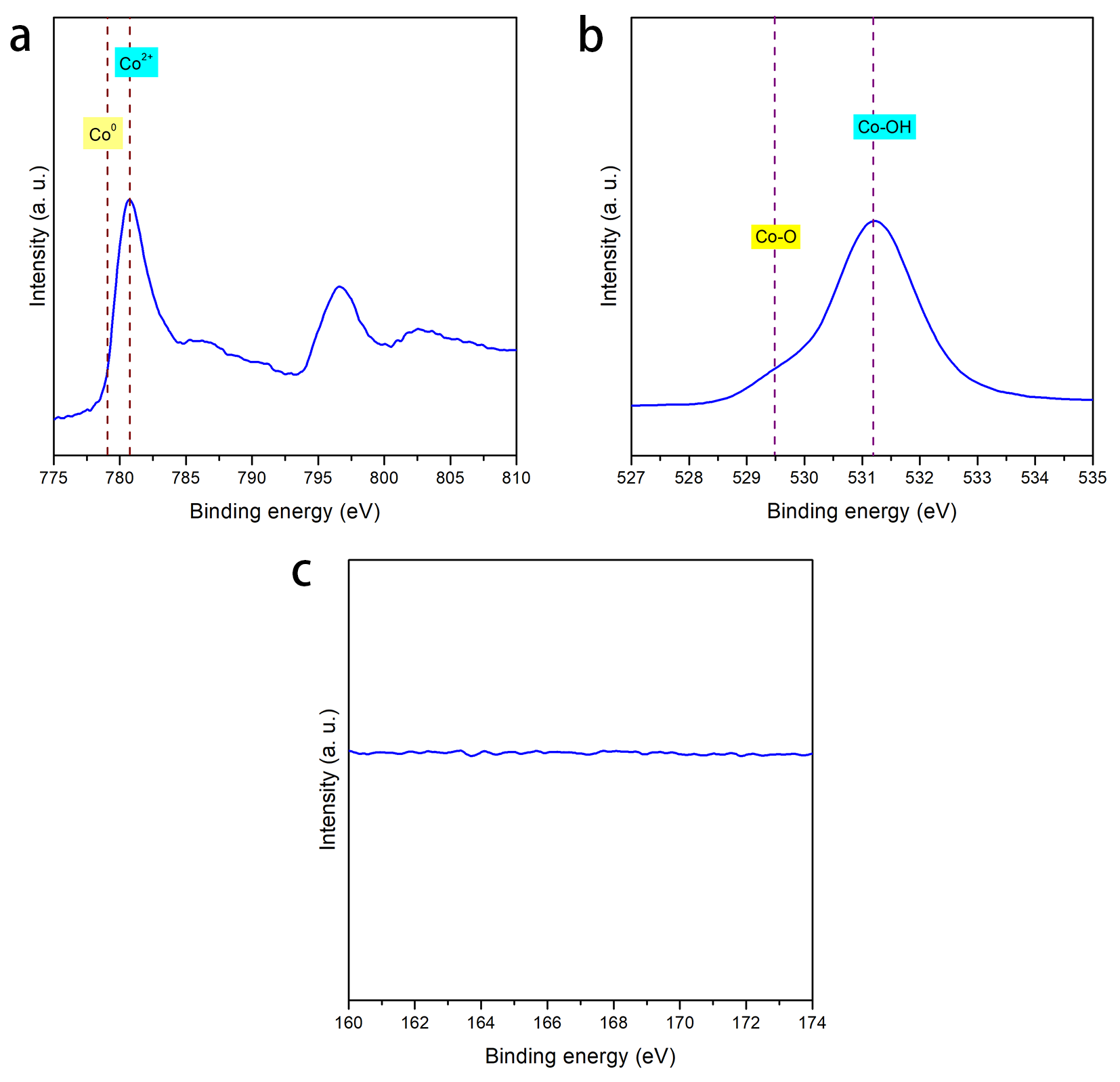


**Figure S14.** High-resolution XPS of (a) Co 2p, (b) O1s, and (c) S 2p of post-reaction CoS_2_. The catalyst was washed with water and stored under vacuum prior to measurement.


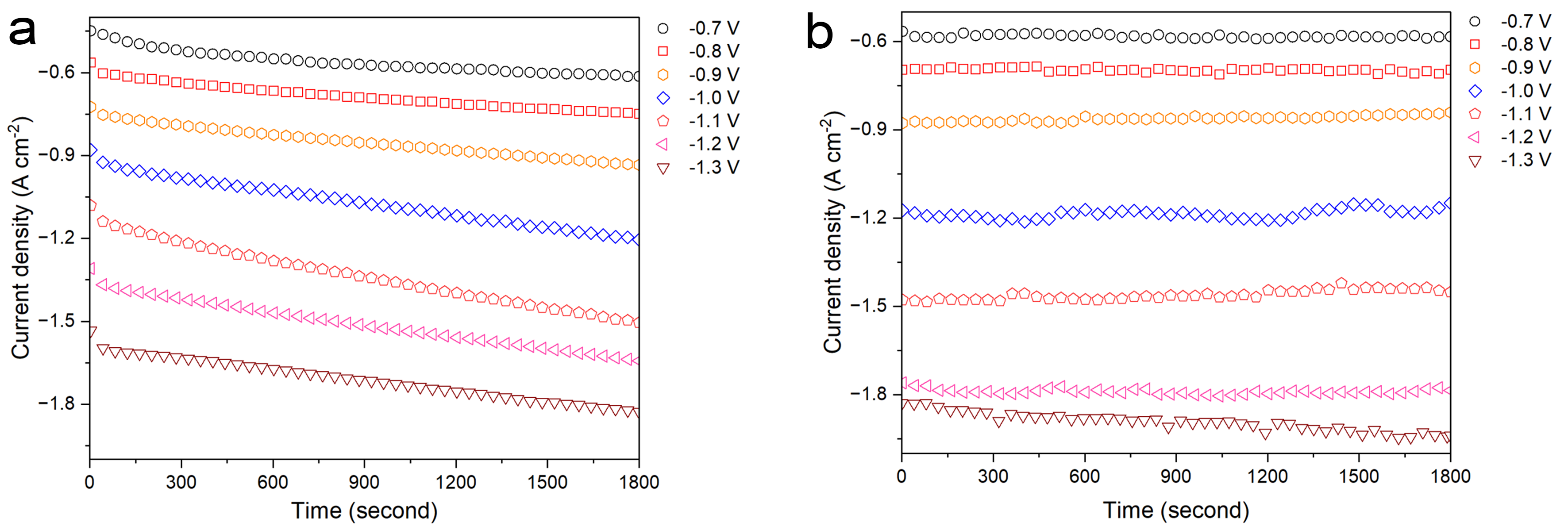


**Figure S15.** Chronoamperometry curves for activated (a) CoS_2_ and (b) Pd-CoS_2_ in 1.0 M KOH with 0.5 M KNO_3_.


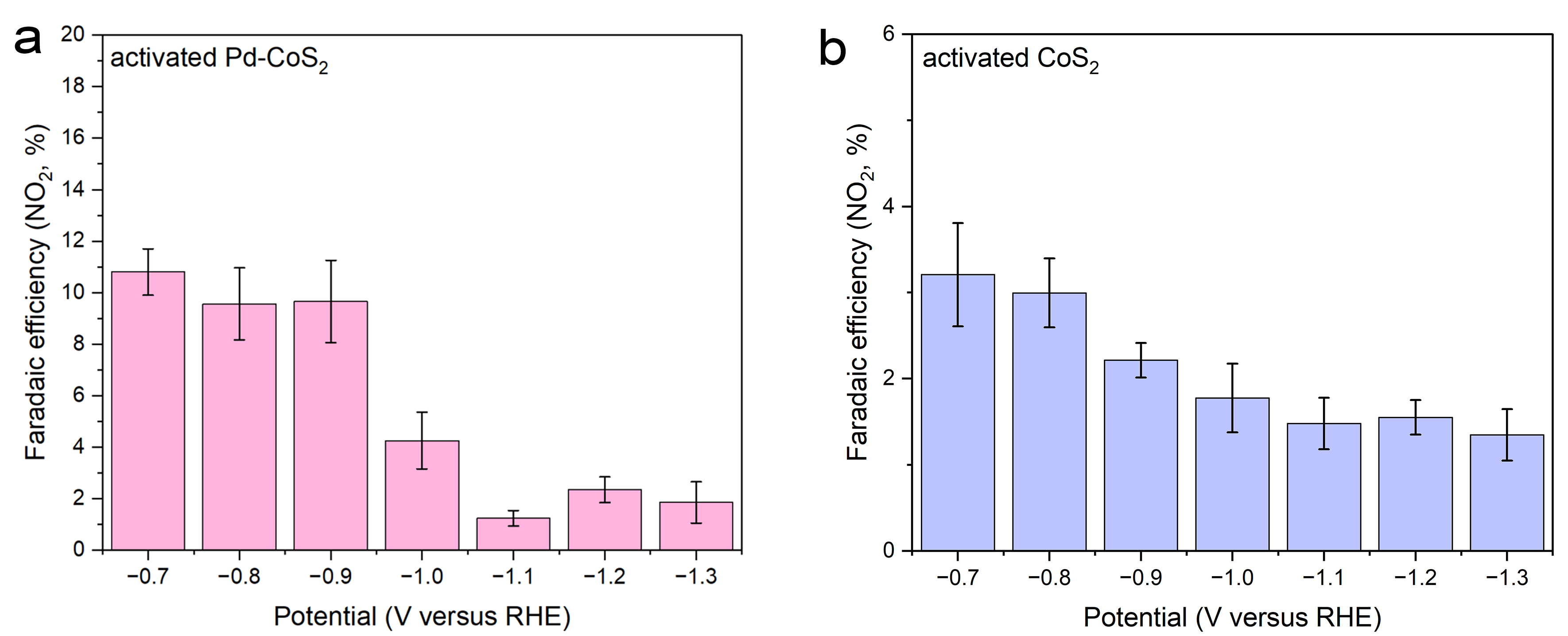


**Figure S16.** NO_2_^-^ Faradaic efficiency of activated (a) Pd-CoS_2_ and (b) CoS_2_.


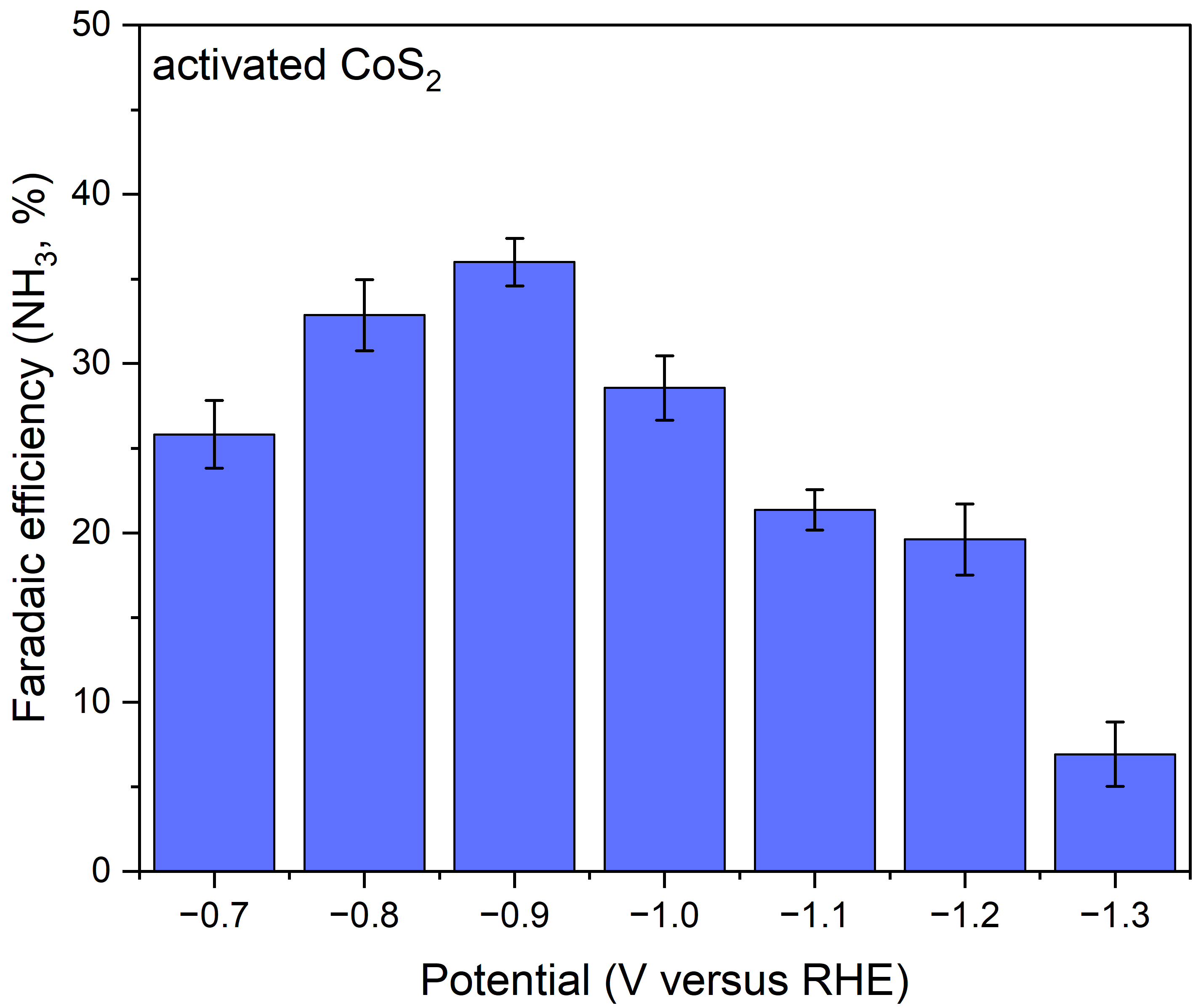


**Figure S17.** Faradaic efficiency for ammonia production on activated CoS_2_ catalyst across applied potentials.


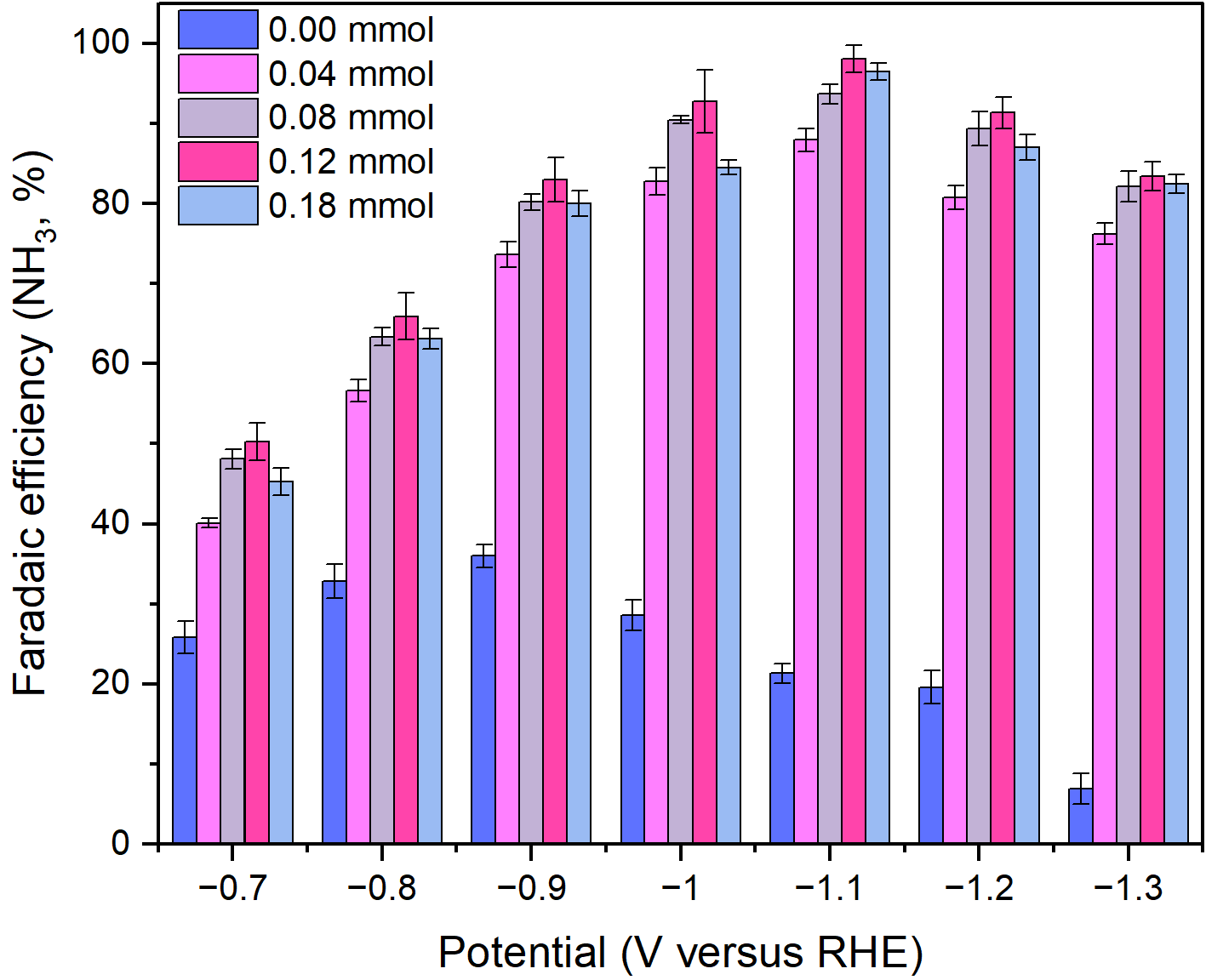


**Figure S18.** Faradaic efficiency comparison for ammonia production on activated CoS_2_ with different Pd usages across applied potentials.


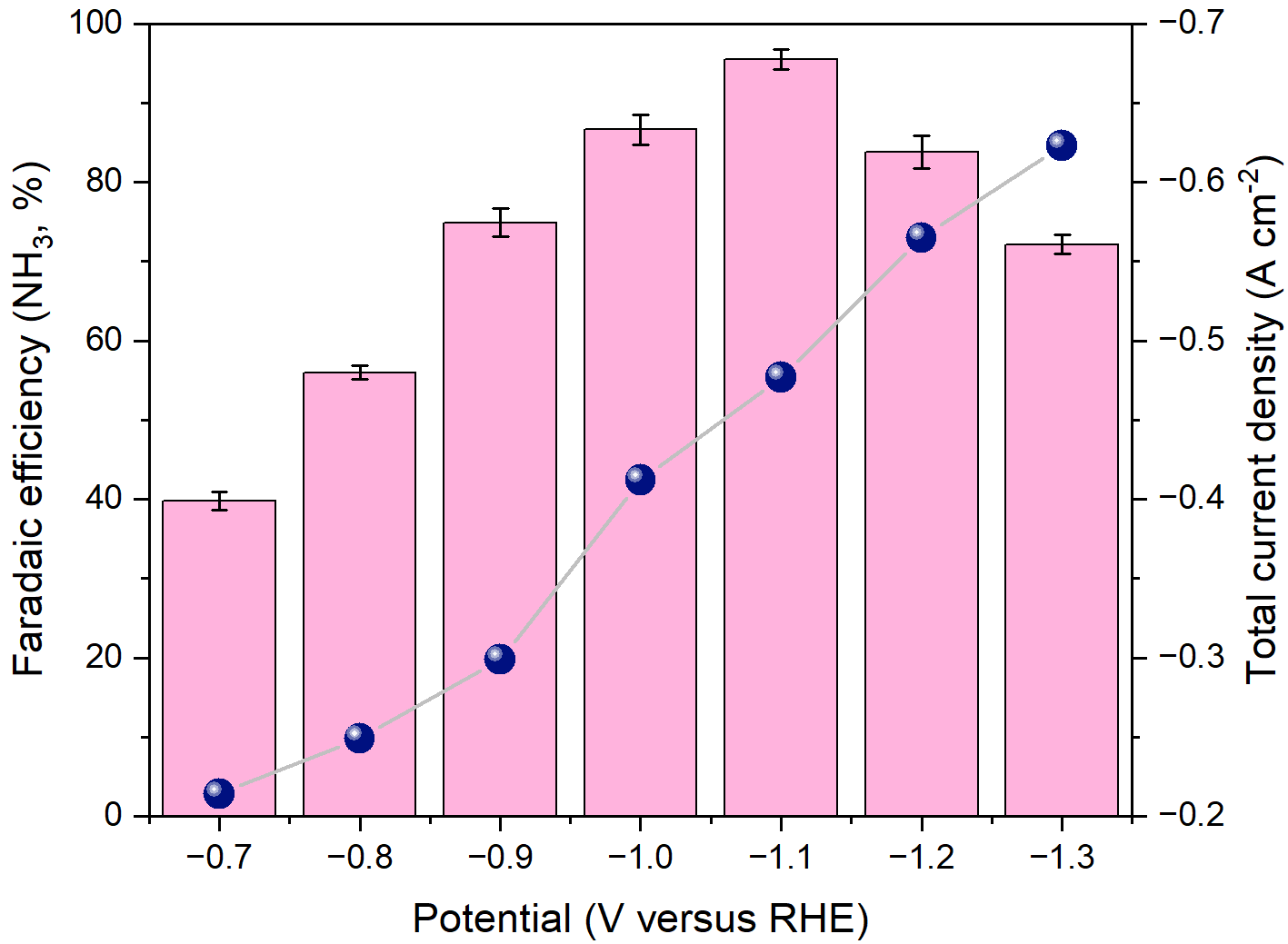


**Figure S19.** Faradaic efficiency and total current density of activated Pd-CoS_2_ in 1.0 M KOH containing 0.1 M KNO_3_.


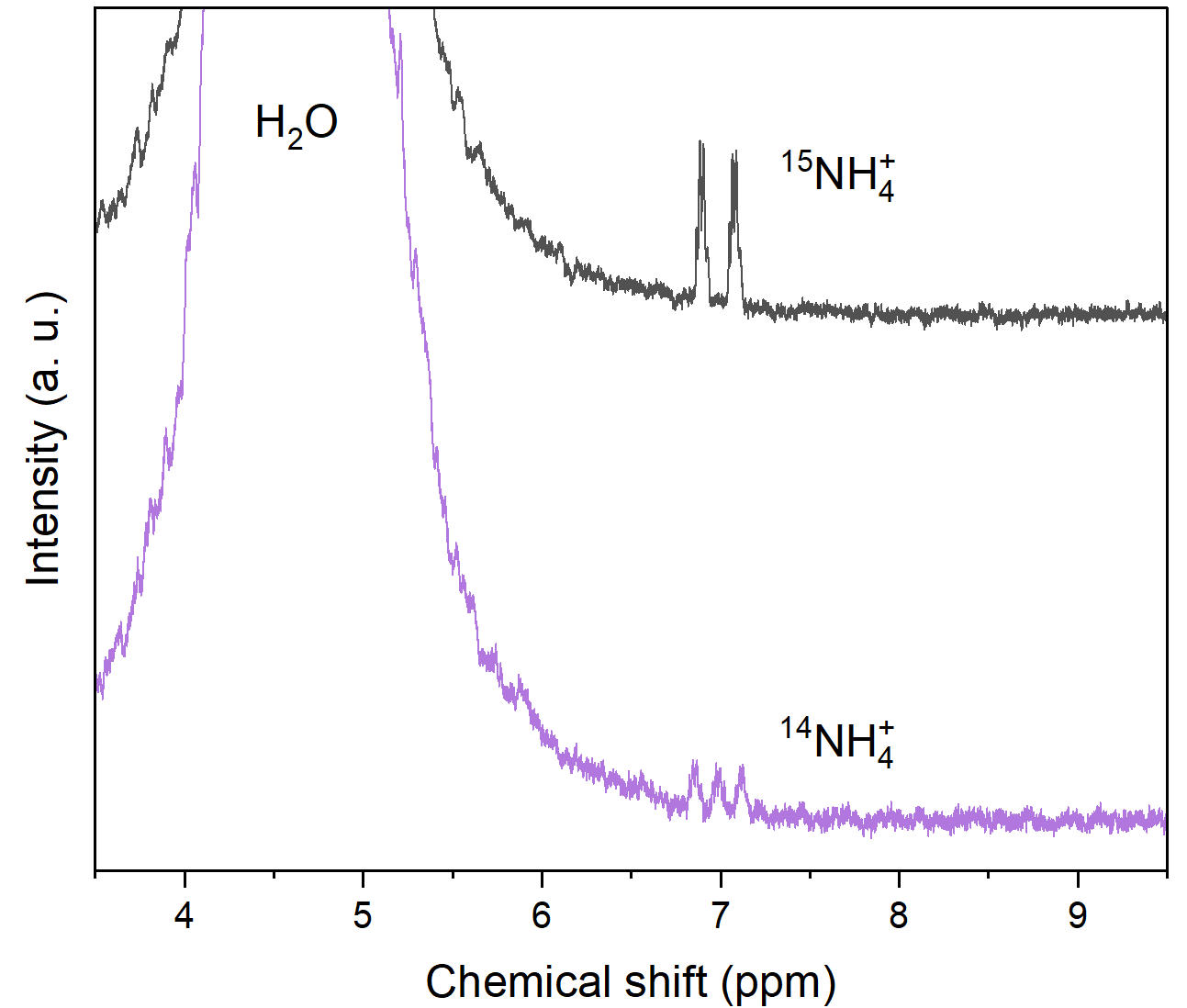


**Figure S20.** ^1^H NMR data comparison with ^14^NO_3_^-^ and ^15^NO_3_^-^ as the reactant, respectively.


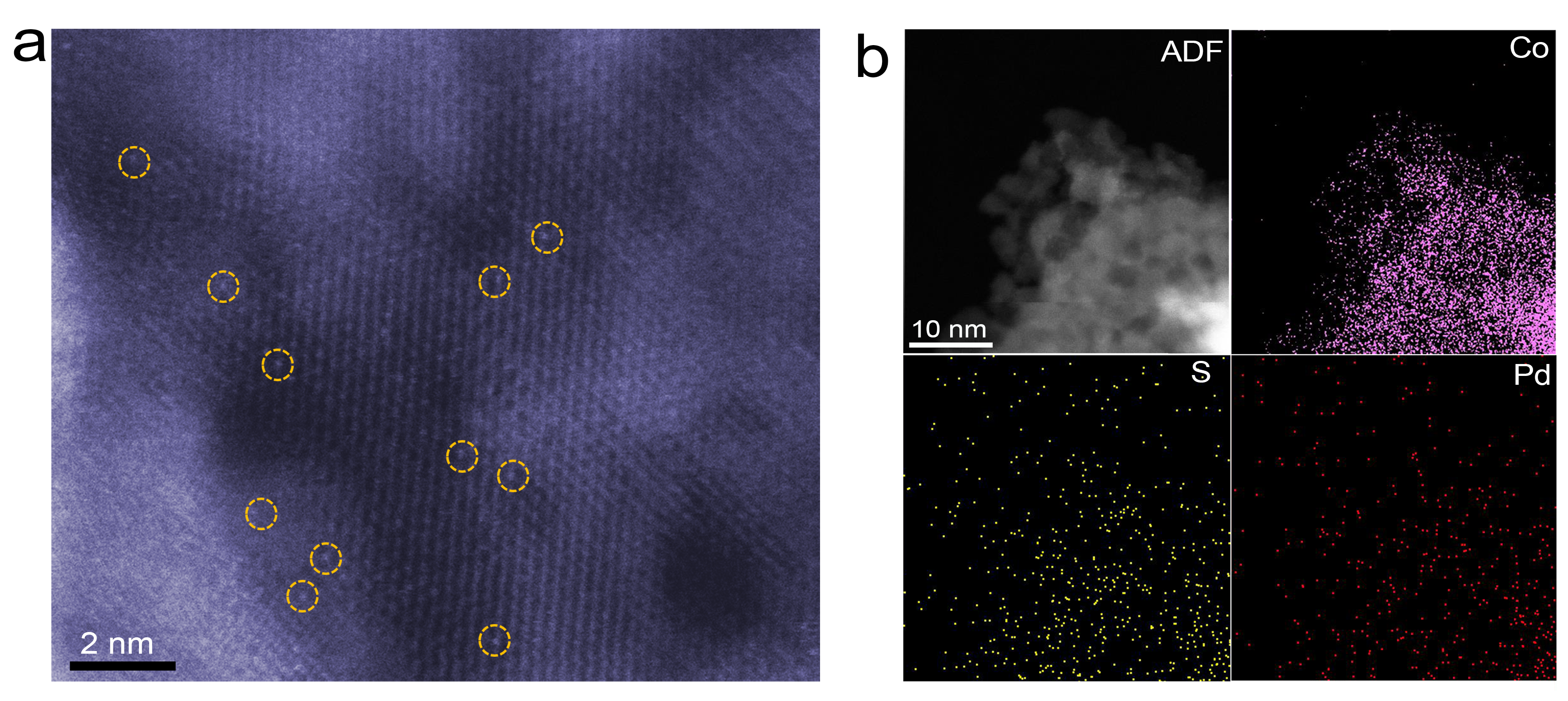


**Figure S21.** Atomic-resolution HAADF-STEM image (a) and elemental mapping (b) of Pd-CoS_2_ after long-term test.


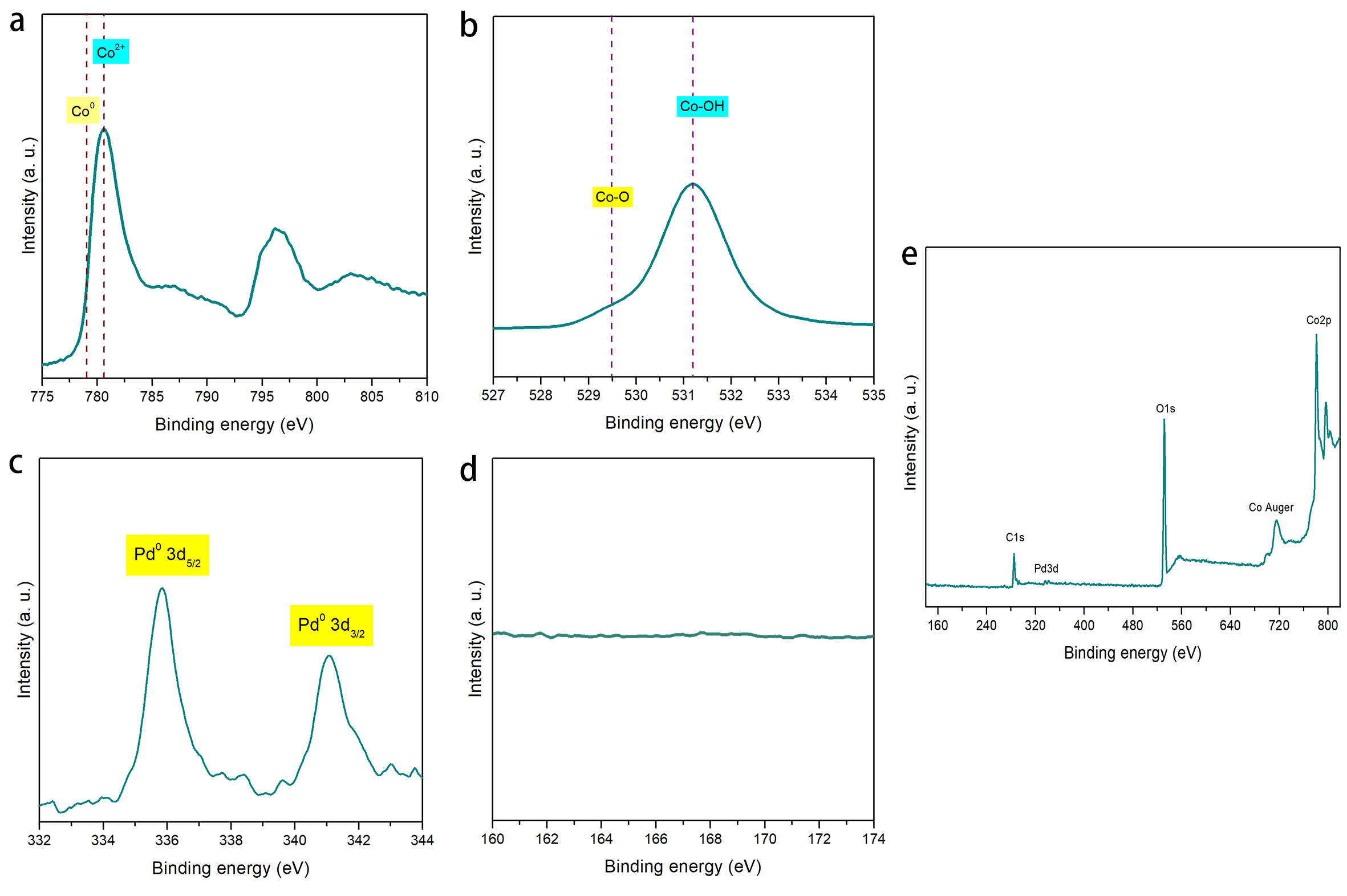


**Figure S22.** High-resolution XPS spectra of (a) Co 2p, (b) O 1s, (c) Pd 3d, and (d) S 2p for Pd-CoS_2_ after long-term stability test. (e) XPS survey. The catalyst was washed with water and stored under vacuum prior to measurement.


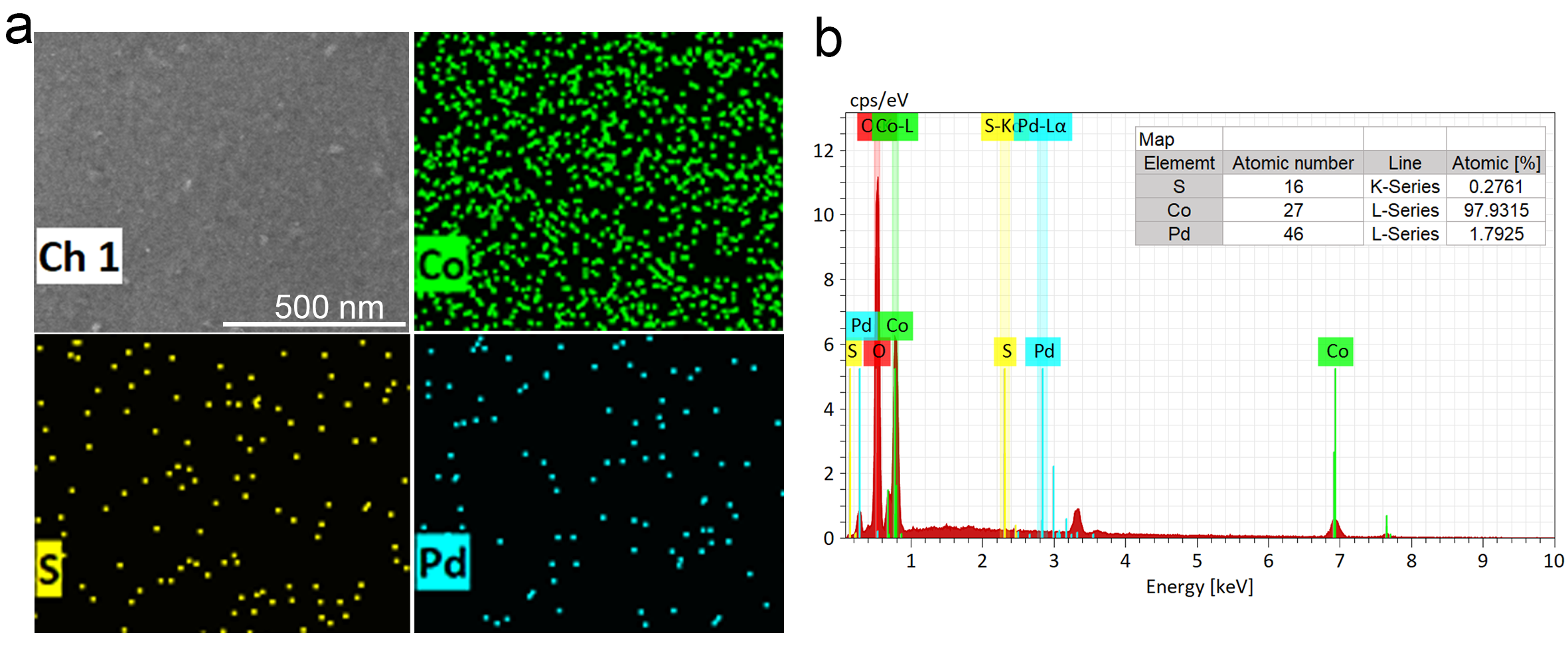


**Figure S23.** Elemental mapping analysis of Pd-CoS_2_ after long-term stability test. Notably, the catalyst was washed with water and stored under vacuum prior to measurement.


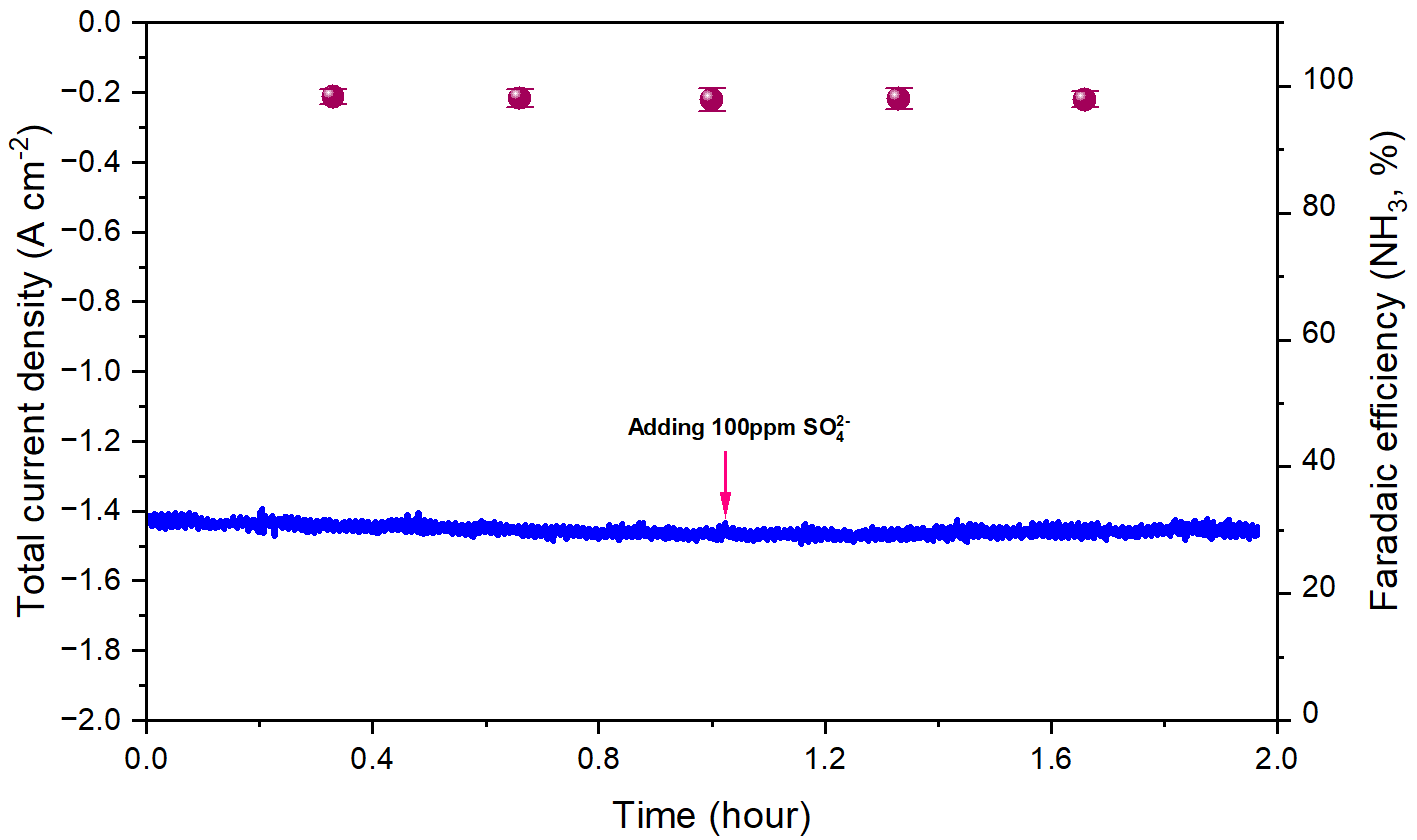


**Figure S24.** Sulfate ion addition experiments for activated Pd-CoS_2_ measured at -1.1 V vs. RHE.


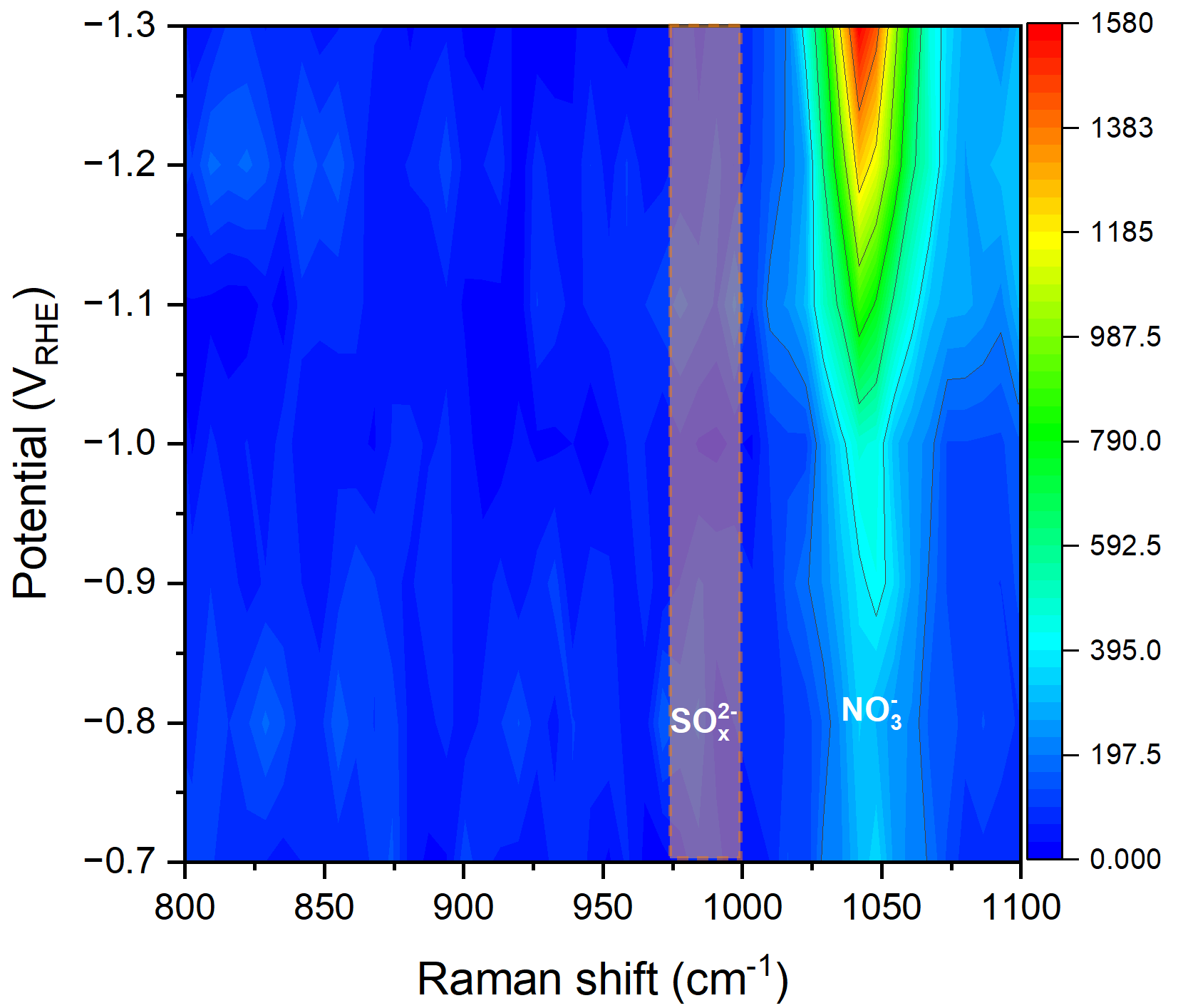


**Figure S25.** In-situ Raman spectra of Pd-CoS_2_ under applied potentials.


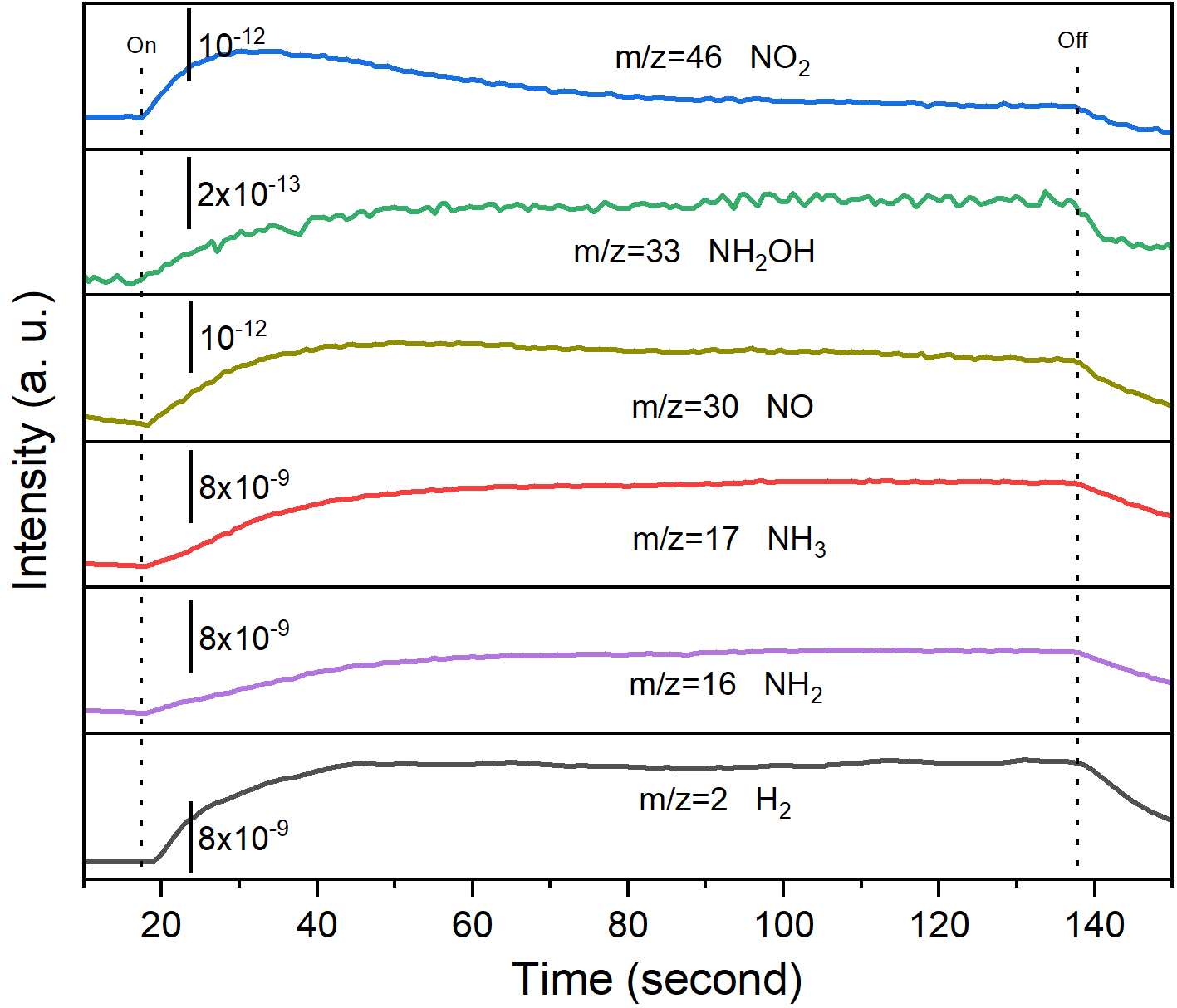


**Figure S****26.** Online DEMS of activated CoS_2_.


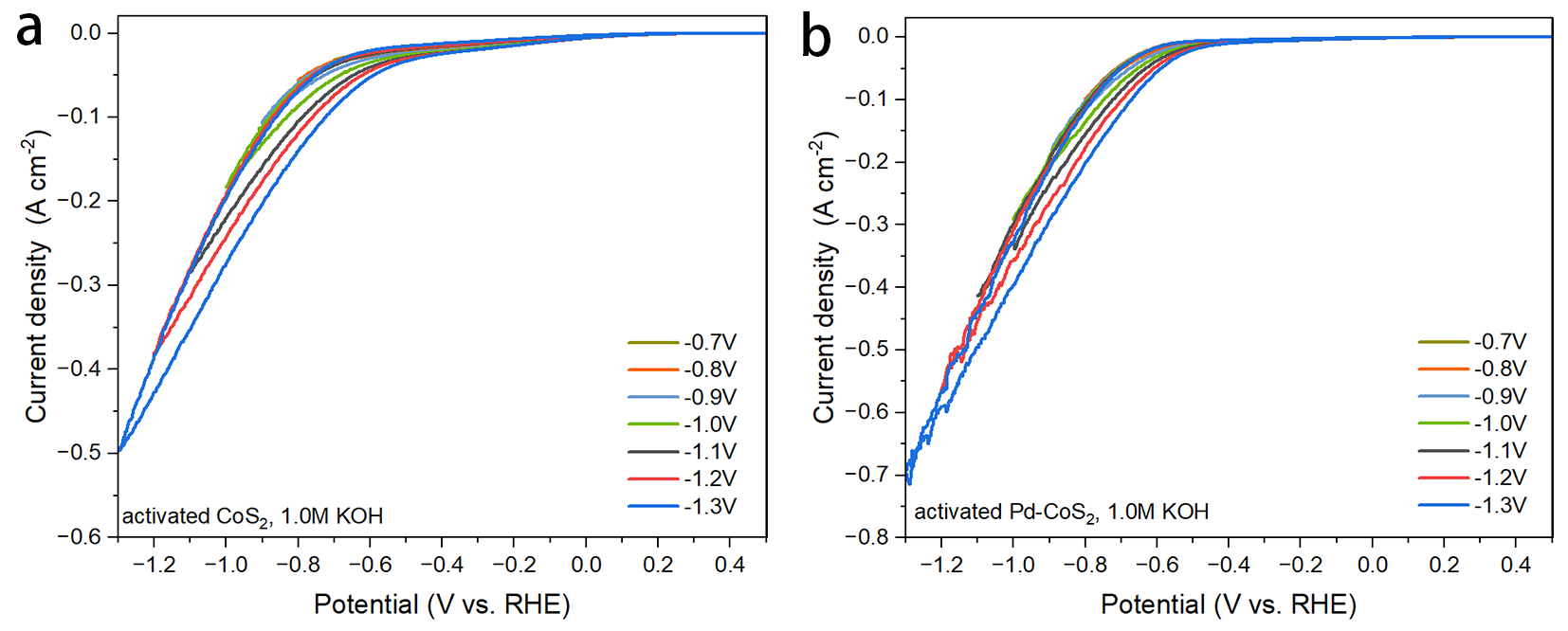


**Figure S27.** CV curves of activated (a) CoS_2_ and (b) Pd-CoS_2_ in 1.0 M KOH under different cathodic limits.


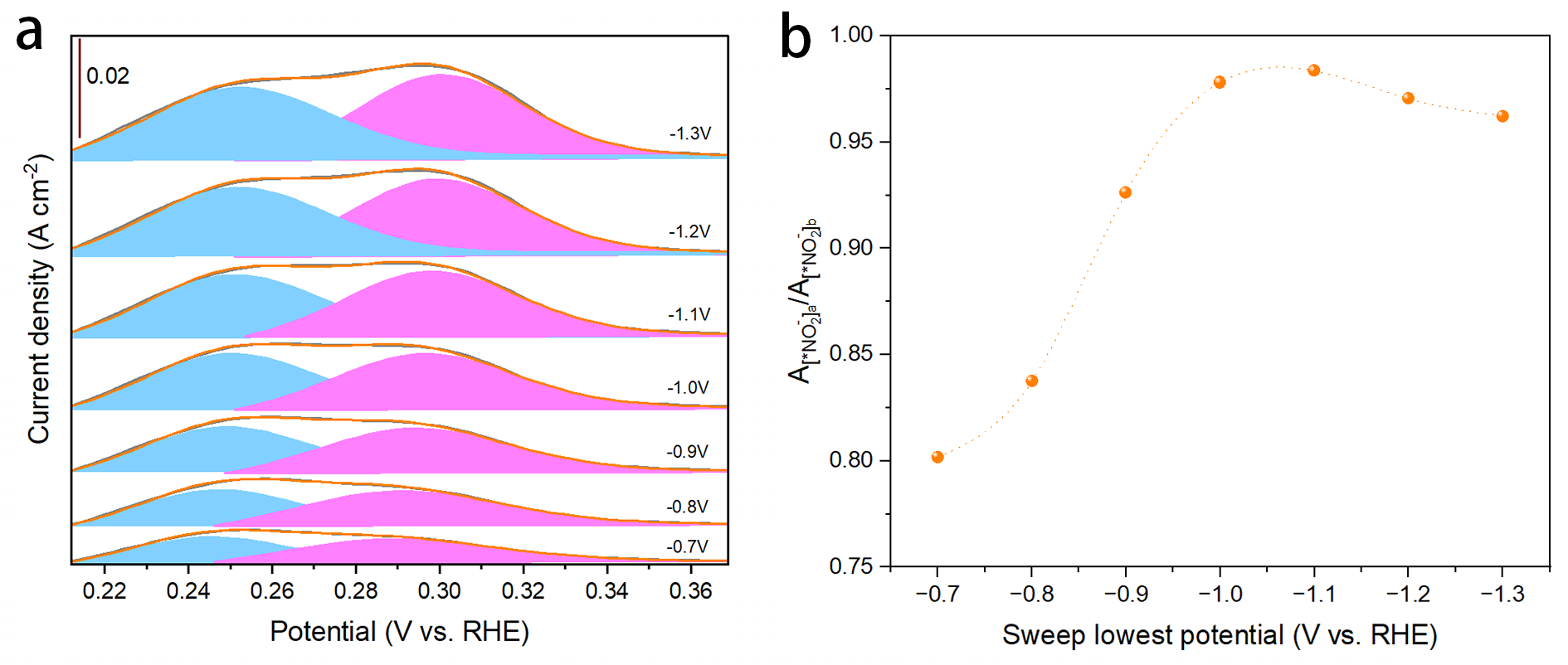


**Figure S28.** (a) Peak fitting and (b) estimated peak area ratios of ([*NO_2_^-^]_a_) to ([*NO_2_^-^]_b_) in Figure 3b.


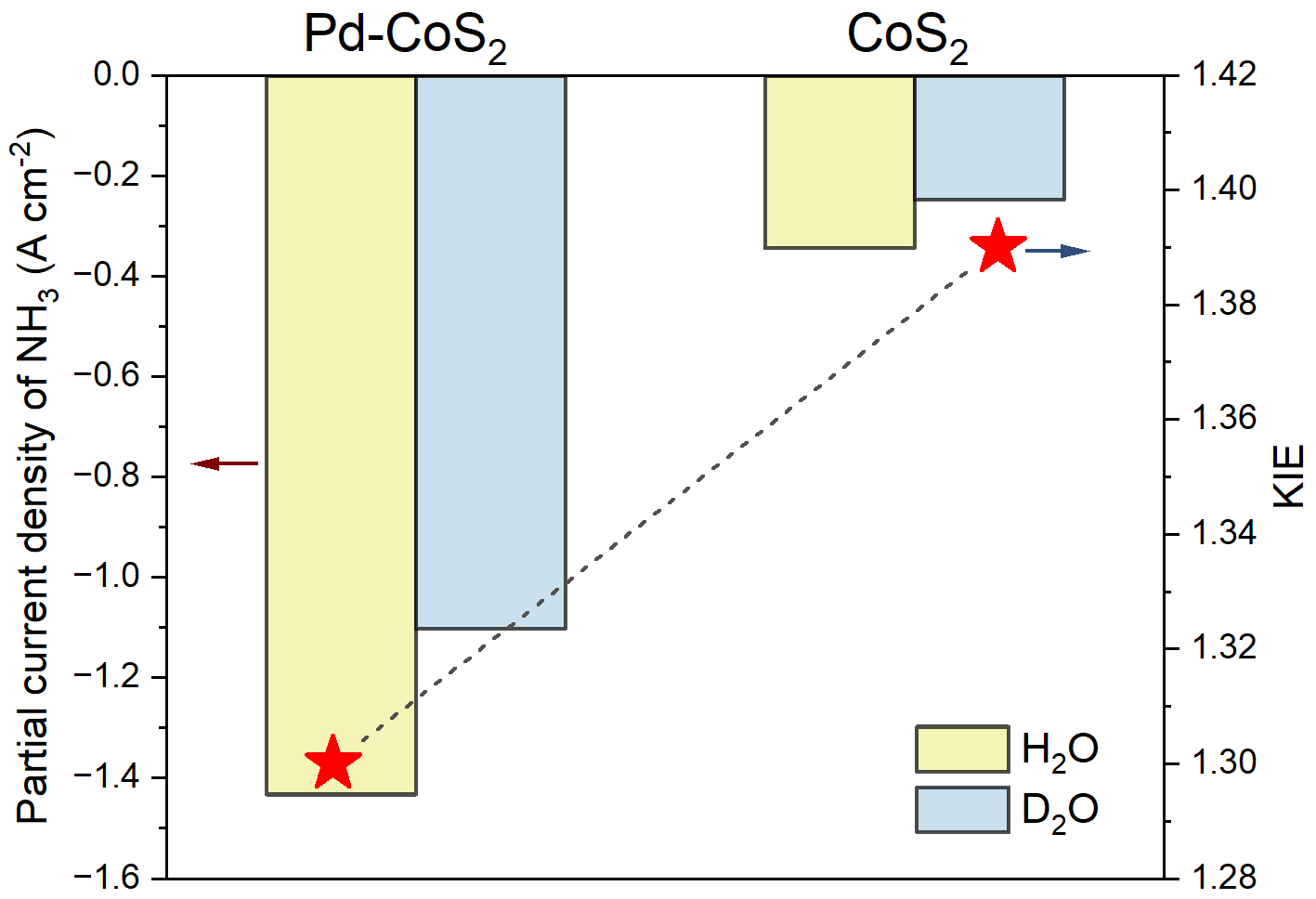


**Figure S29.** Calculated KIE values of activated Pd-CoS_2_ and CoS_2_ in the H_2_O-based and D_2_O-based electrolyte containing 1.0 M KOH with 0.5 M KNO_3_.


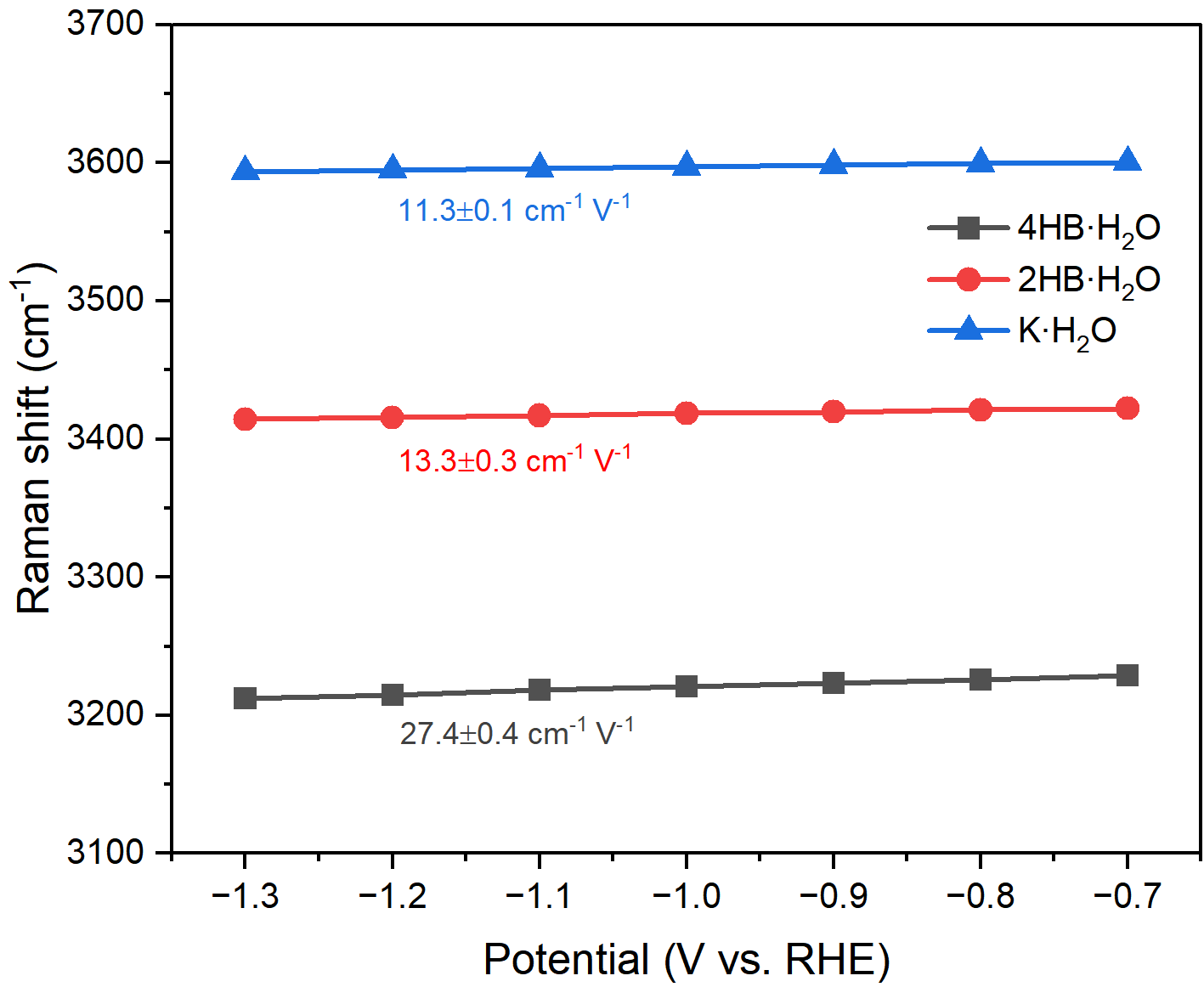


**Figure S30.** Potential-dependent Stark effect shift analysis of activated CoS_2_ from Figure 3e.


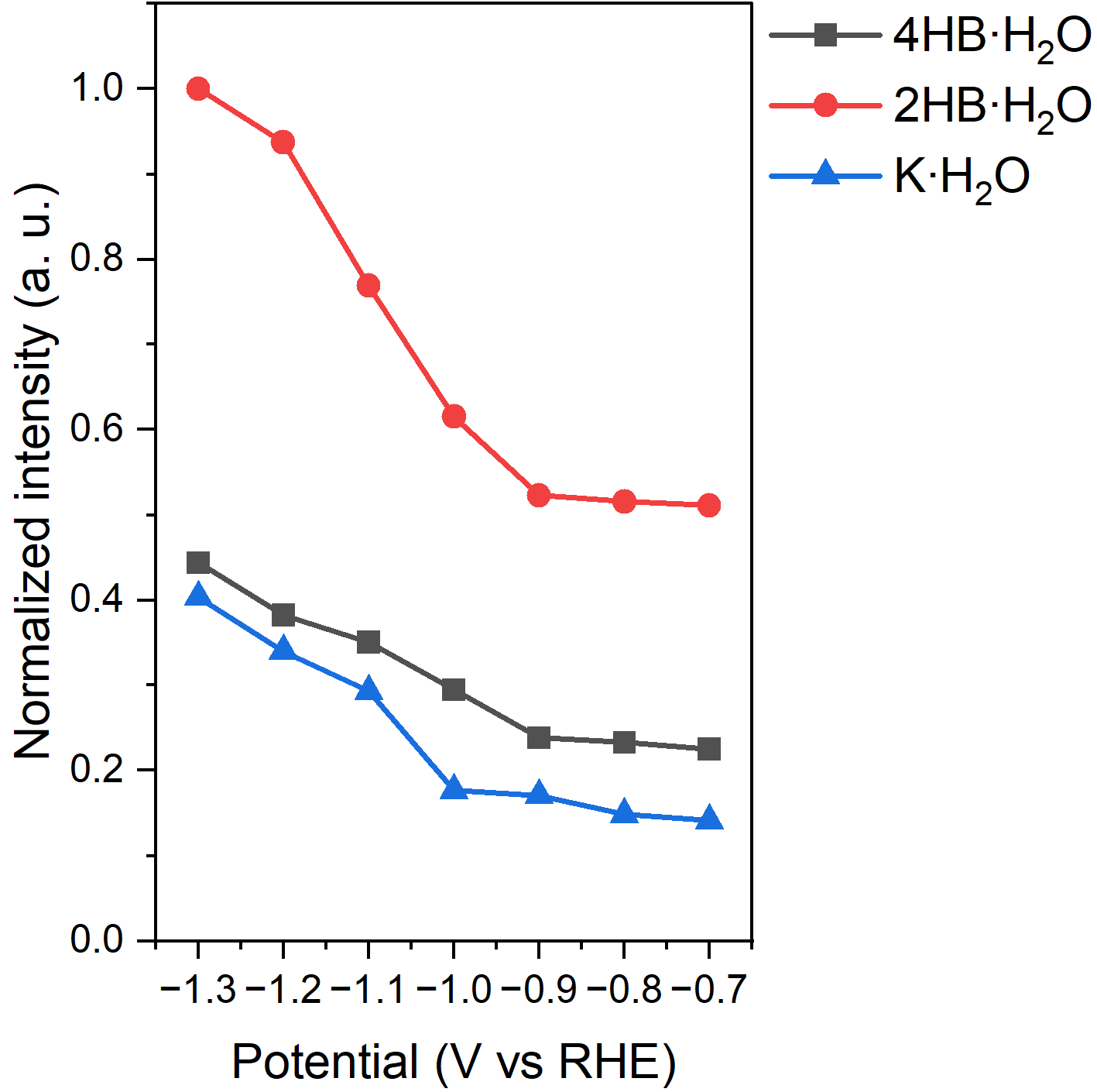


**Figure S31.** Normalized intensity of different water molecules for activated CoS_2_.


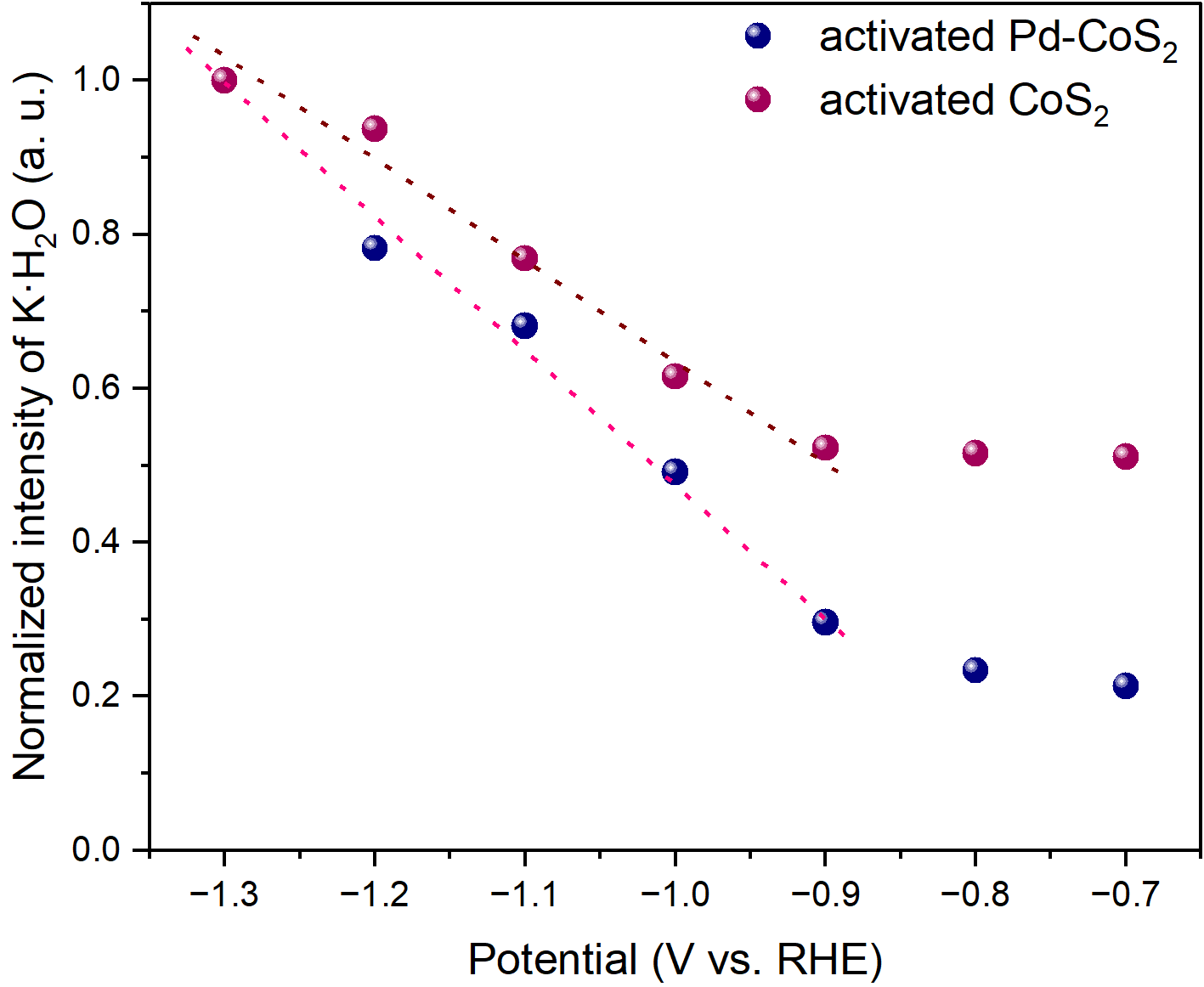


**Figure S32.** Comparison of potential-dependent normalized intensity change of K·H_2_O water molecules for activated CoS_2_ and Pd-CoS_2_.


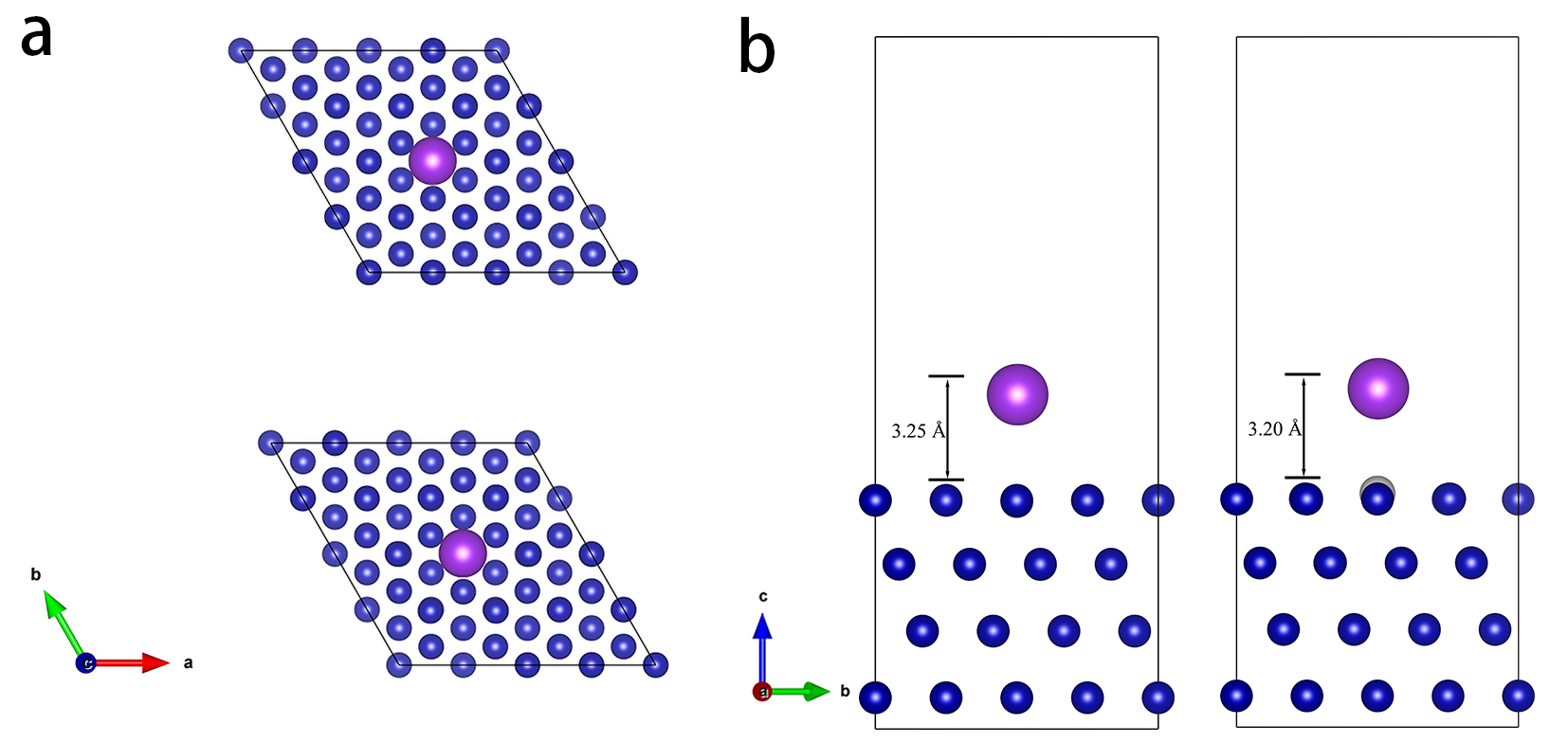


**Figure S33.** Theoretical analysis of the introduction of Pd altering the distance of K^+^ to the electrode surface. a: top views of Co (up) and Pd-doped Co (down); b: side views of Co (left) and Pd-doped Co (right).


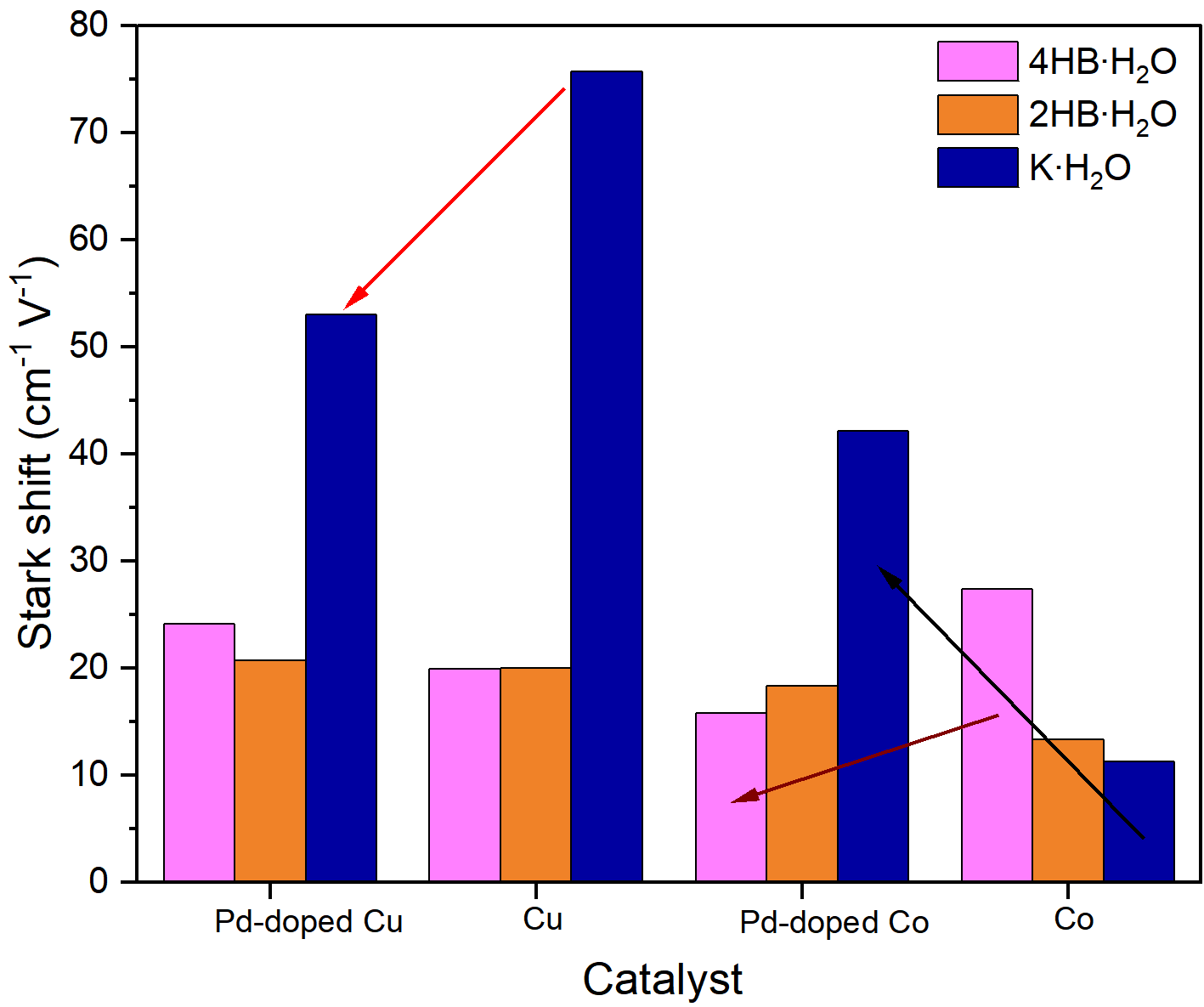


**Figure S34.** Stark shift comparison for various catalysts.


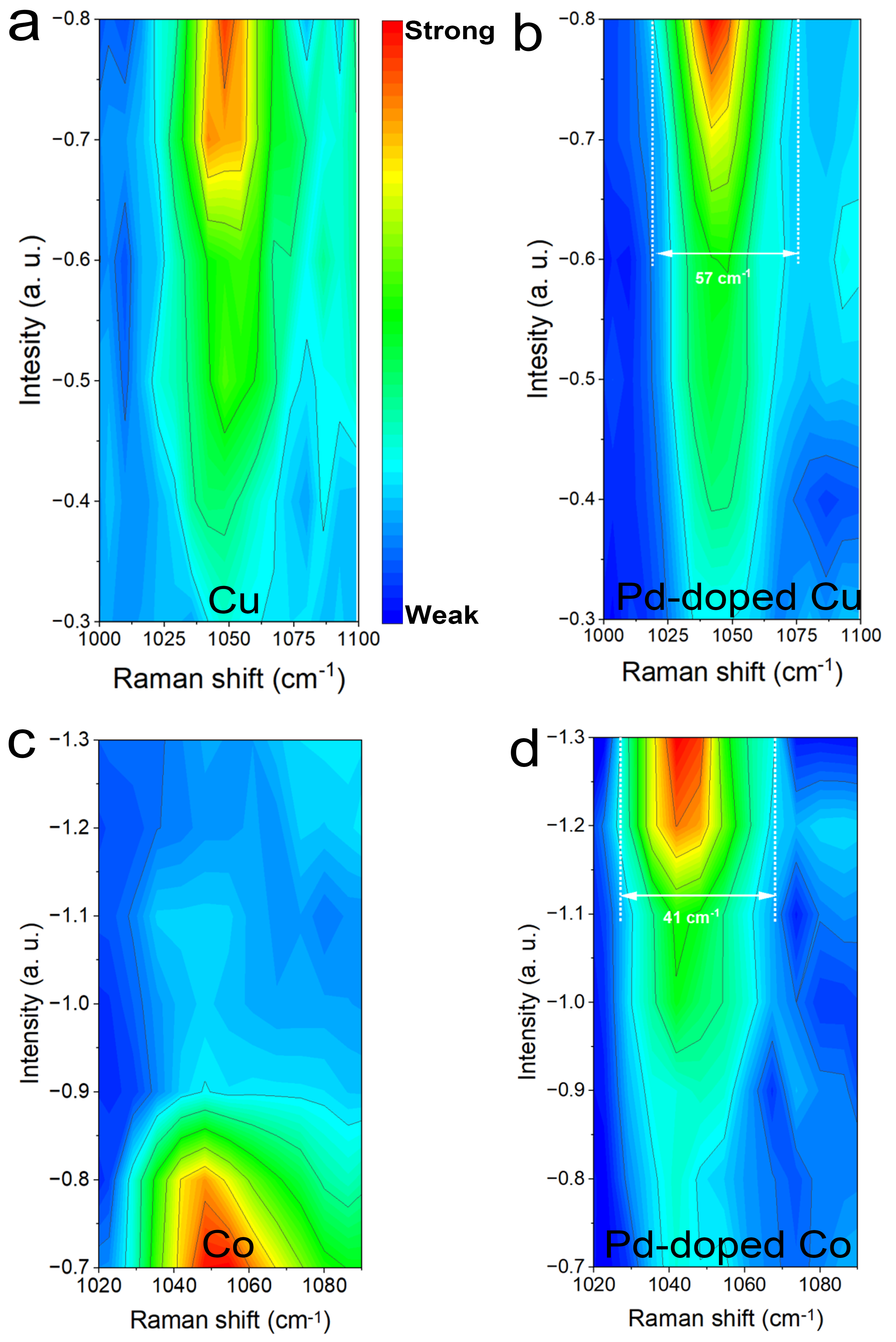


**Figure S35.** In-situ Raman spectra to monitor *NO_3_^-^ on Cu-based (a, b) and Co-based (c, d) catalysts.


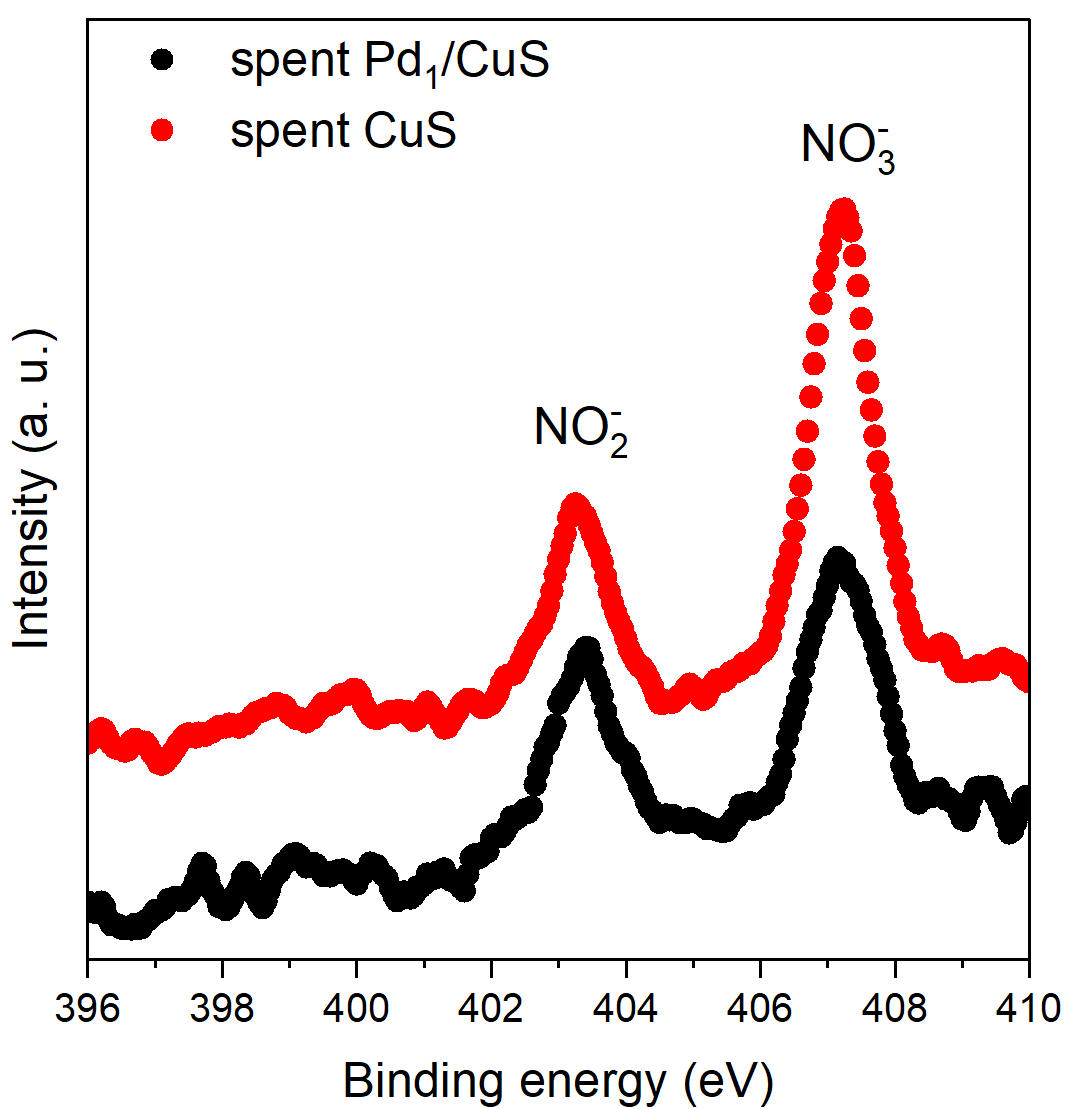


**Figure S36.** High-resolution N 1s XPS spectra of spent CuS and Pd_1_/CuS.


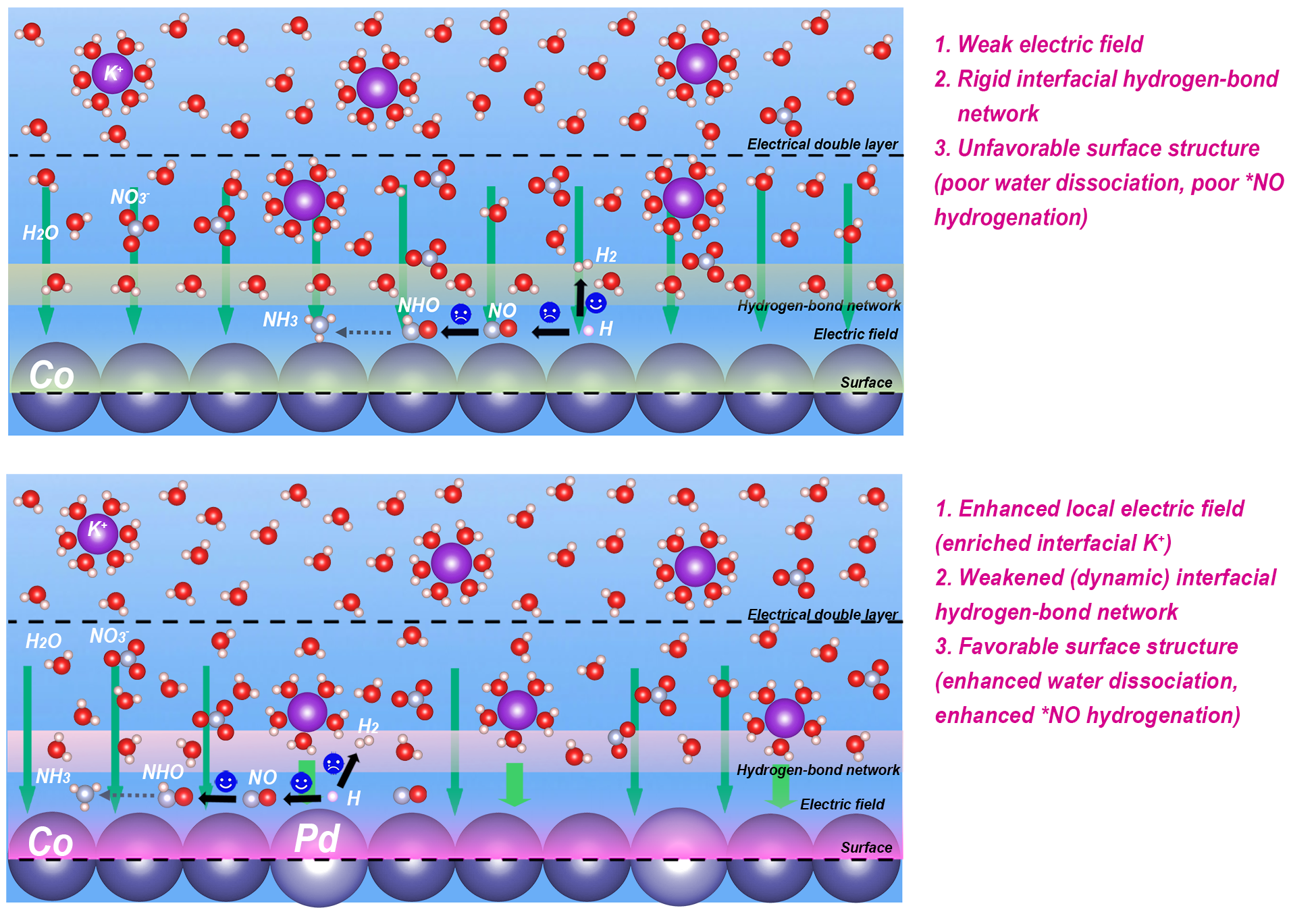


**Figure S37.** Schematic illustration of the role of Pd incorporation in the Cu catalyst.


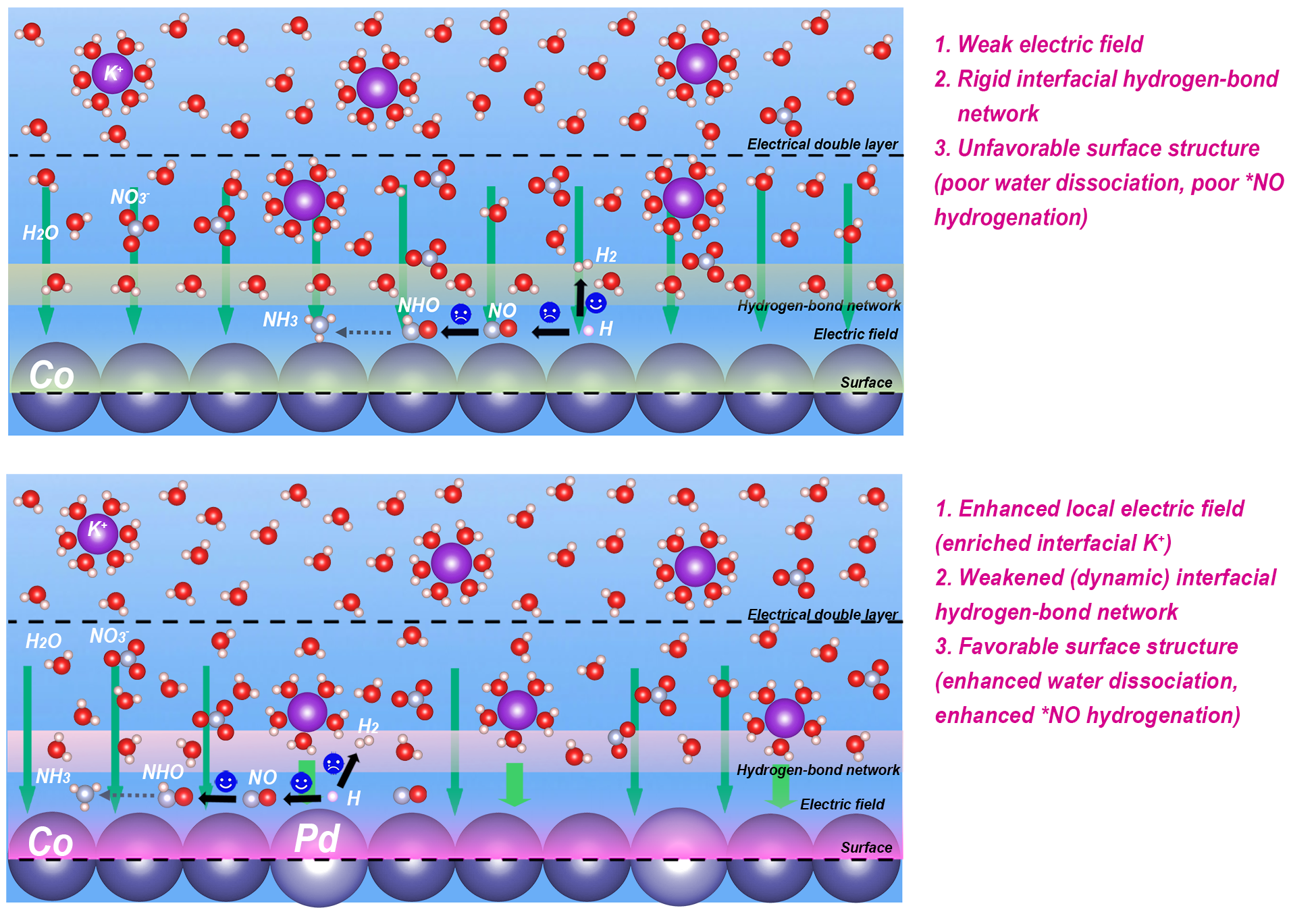


**Figure S38.** Schematic illustration of the role of Pd incorporation in the Co catalyst.


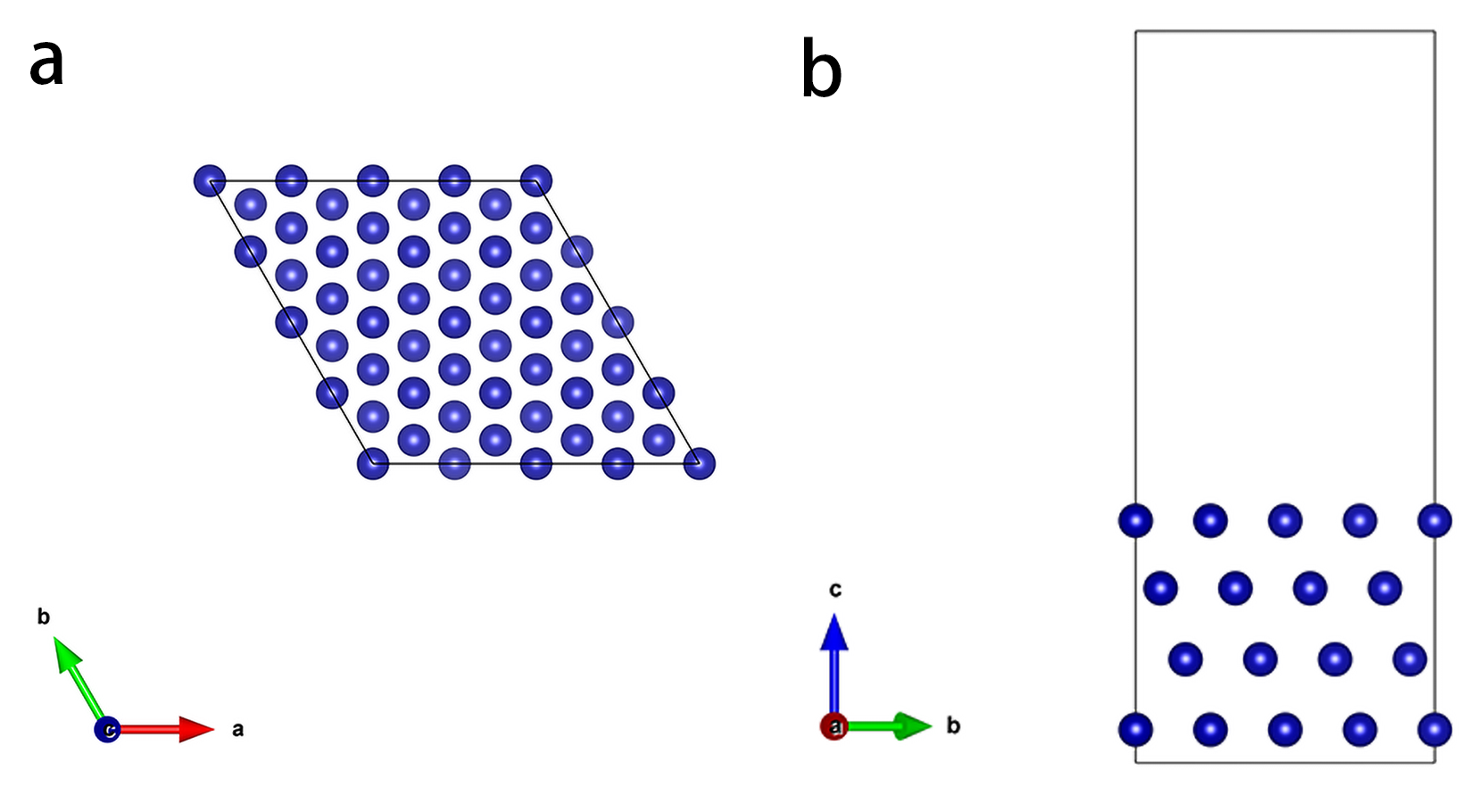


**Figure S39.** Modeled surface facet. (a) Top view, (b) side view.


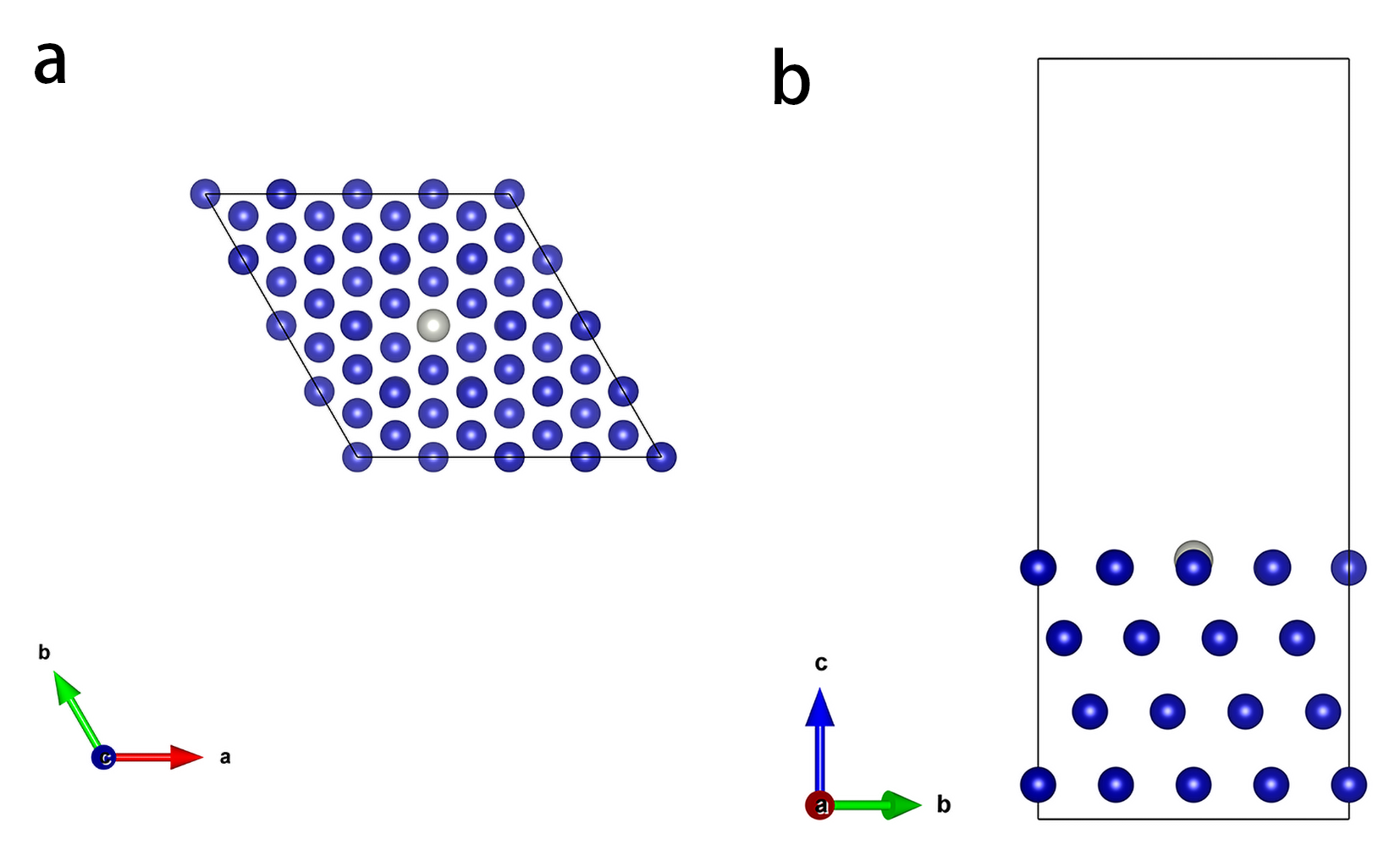


**Figure S40.** Pd site configuration and loading. (a) Top view, (b) side view.


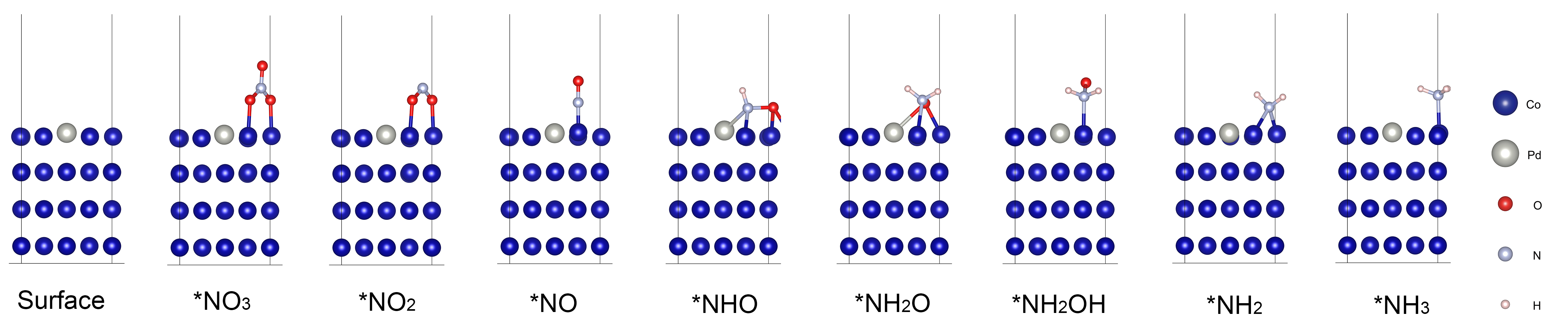


**Figure S41.** DFT optimized geometries of intermediates on Pd-doped Co surface.


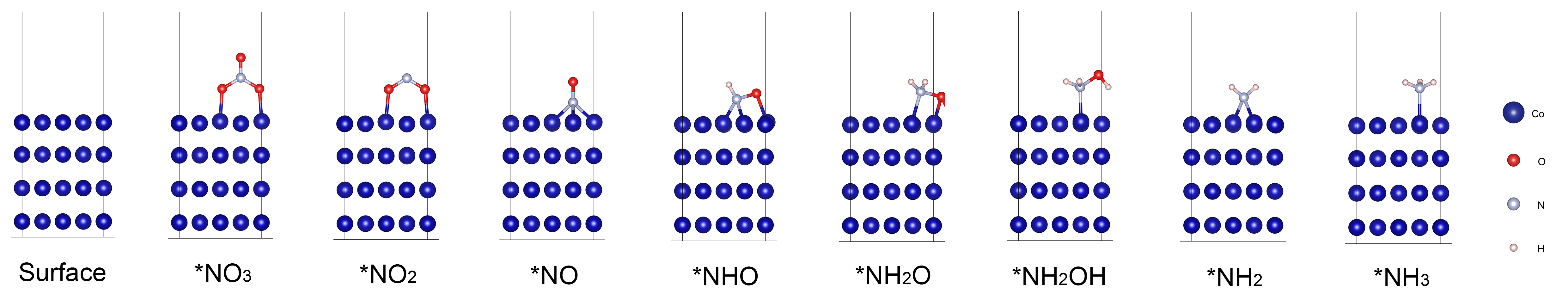


**Figure S42.** DFT optimized geometries of intermediates on metallic Co surface.


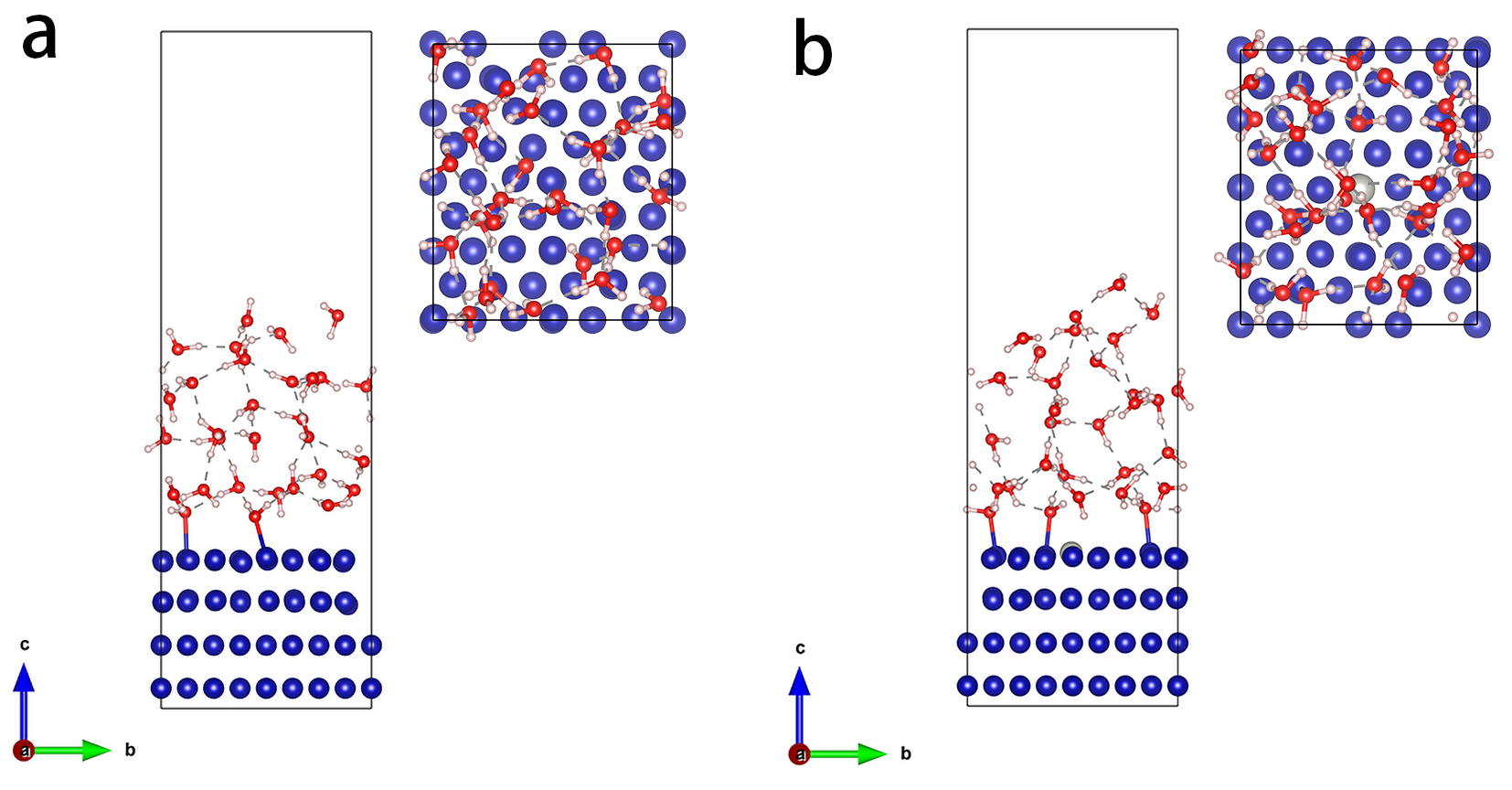


**Figure S43.** The solvation/electric-field models of (a) Co and (b) Pd-doped Co.


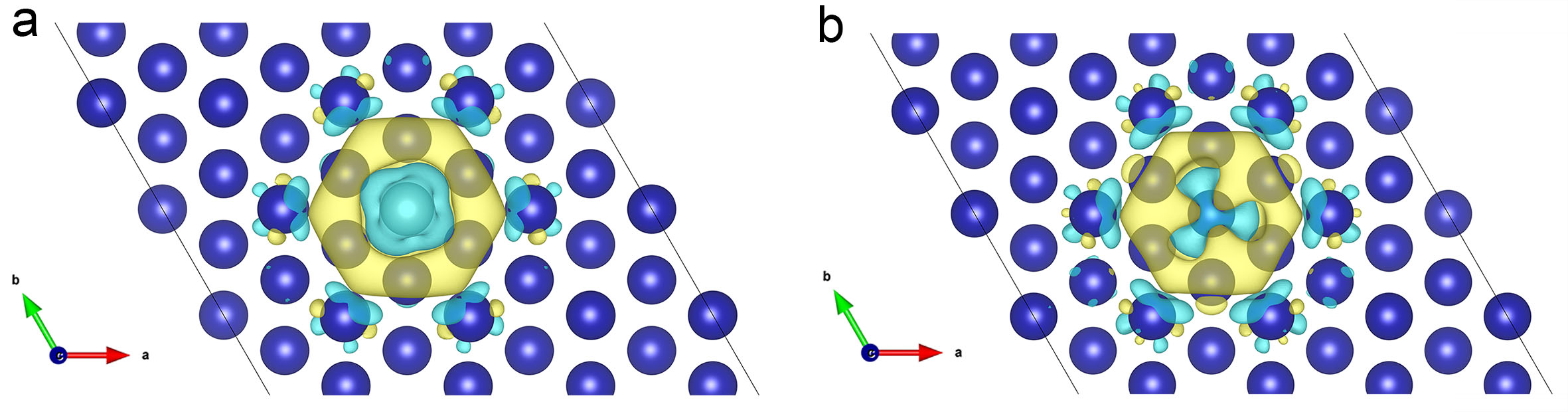


**Figure S44.** Differential charge density analyses of (a) Pd-doped Co and (b) Co structures. Color definition: yellow: electron accumulation, cyan: electron depletion.


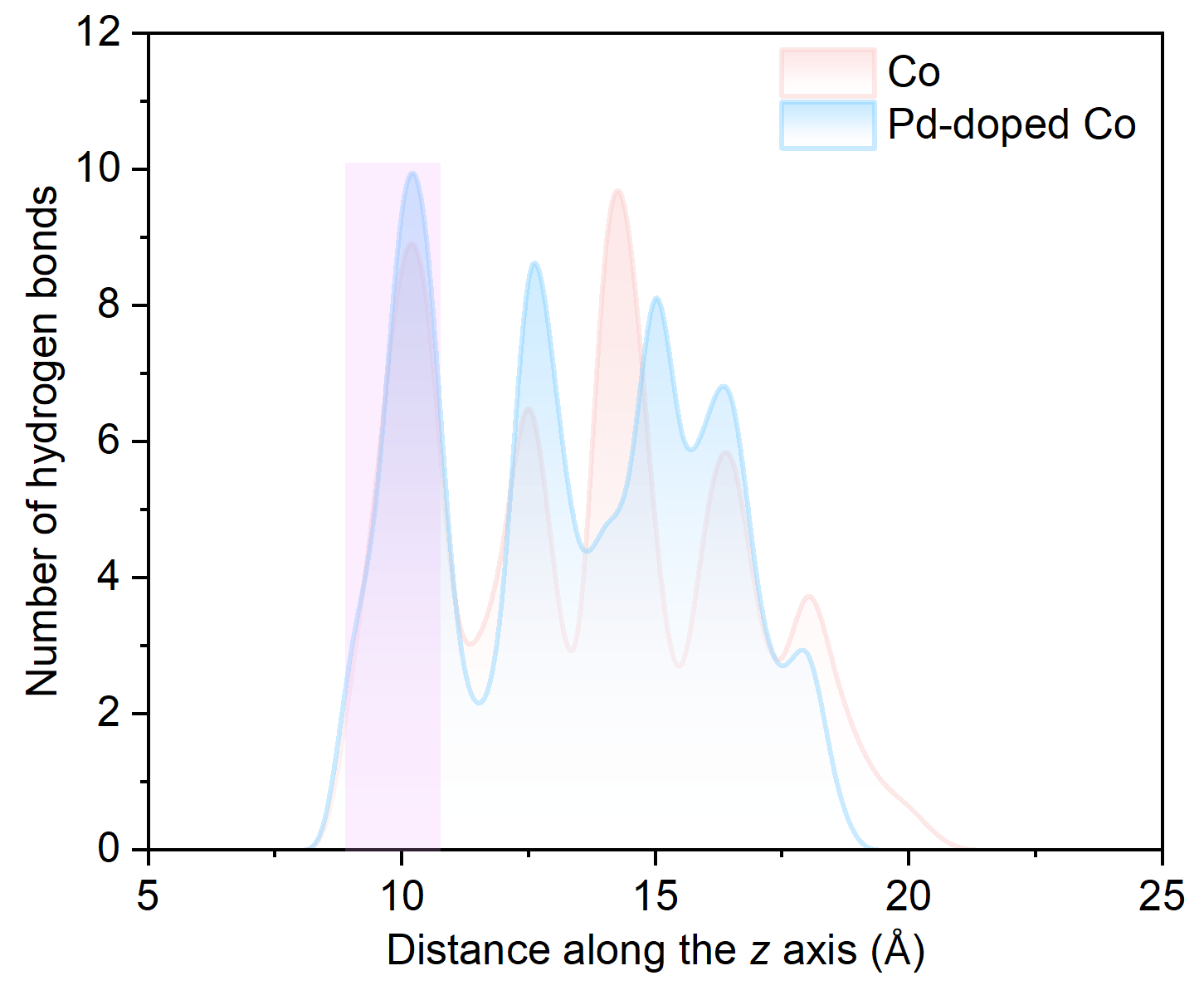


**Figure S45.** The number of hydrogen bond along the z direction normal on the catalyst surface for Co and Pd-doped Co, respectively, derived from AIMD simulations.

**Table S1.** ICP result of Pd-CoS_2_.

| Sample | Element | Mass concentration (mg L^-1^) | Molar concentration  (mmol L^-1^) | Molar ratio  (%) |
| --- | --- | --- | --- | --- |
| Pd-CoS_2_ | Co | 92.604 | 1.571 | 0. 75 |
|  | Pd | 1.259 | 0.012 |  |

**Supplementary References**

1. a) G. Kresse, J. Furthmüller, *Phys. Rev. B* **1996**, *54*, 11169–11186; b) G. Kresse, J. Furthmüller, *Comput. Mater. Sci.* **1996**, *6*, 15–50; c) G. Kresse, D. Joubert, *Phys. Rev. B* **1999**, *59*, 1758–1775.
2. J. P. Perdew, K. Burke, M. Ernzerhof, *Phys. Rev. Lett.* **1996**, *77*, 3865–3868.
3. S. Grimme, J. Antony, S. Ehrlich, H. Krieg, *J. Chem. Phys.* **2010**, *132*, 154104.
4. a) K. Momma, F. Izumi, *J. Appl. Crystallogr.* **2011**, *44*, 1272–1276; b) V. Wang, N. Xu, J.-C. Liu, G. Tang, W.-T. Geng, *Comput. Phys. Commun.* **2021**, *267*, 108033.
5. G. Henkelman, H. Jónsson, *J. Chem. Phys.* **2000**, *113*, 9978–9985.
6. W. Humphrey, A. Dalke, K. Schulten, *J. Mol. Graph.* **1996**, *14*, 33–38.
